# Identifying Candidate Genetic Markers of CDV Cross-Species Pathogenicity in African Lions

**DOI:** 10.3390/pathogens9110872

**Published:** 2020-10-23

**Authors:** Julie K. Weckworth, Brian W. Davis, Melody E. Roelke-Parker, Rebecca P. Wilkes, Craig Packer, Ernest Eblate, Michael K. Schwartz, L. Scott Mills

**Affiliations:** 1 Wildlife Biology Program, Department of Ecosystem and Conservation Sciences, W. A. Franke College of Forestry and Conservation, University of Montana, Missoula, MT 59812, USA; michael.k.schwartz@usda.gov; 2 United States Department of Agriculture, Forest Service, National Genomics Center for Wildlife and Fish Conservation, Rocky Mountain Research Station, Missoula, MT 59801, USA; 3 Department of Veterinary Integrative Biosciences, Texas A&M University College of Veterinary Medicine, College Station, TX 77843, USA; bdavis@cvm.tamu.edu; 4 Leidos Biomedical Research, Frederick National Laboratory for Cancer Research, Frederick, MD 21702, USA; melody.roelke-parker@nih.gov; 5 Department of Comparative Pathobiology, Animal Disease Diagnostic Laboratory, Purdue University College of Veterinary Medicine, West Lafayette, IN 47907, USA; rwilkes@purdue.edu; 6 Department of Ecology, Evolution and Behavior, University of Minnesota, St. Paul, MN 55108, USA; packer@umn.edu; 7 Tanzania Wildlife Research Institute, Arusha, Tanzania; eblate.ernest@gmail.com; 8 Fisheries, Wildlife, and Conservation Biology Program, Department of Forestry and Environmental Resources, North Carolina State University, Raleigh, NC 27695, USA; scott.mills@mso.umt.edu

**Keywords:** canine distemper virus, African lion, cross-species pathogenicity, multi-host pathogen, evolutionary genetics, viral genomics, spillover

## Abstract

Canine distemper virus (CDV) is a multi-host pathogen with variable clinical outcomes of infection across and within species. We used whole-genome sequencing (WGS) to search for viral markers correlated with clinical distemper in African lions. To identify candidate markers, we first documented single-nucleotide polymorphisms (SNPs) differentiating CDV strains associated with different clinical outcomes in lions in East Africa. We then conducted evolutionary analyses on WGS from all global CDV lineages to identify loci subject to selection. SNPs that both differentiated East African strains and were under selection were mapped to a phylogenetic tree representing global CDV diversity to assess if candidate markers correlated with documented outbreaks of clinical distemper in lions (*n* = 3). Of 54 SNPs differentiating East African strains, ten were under positive or episodic diversifying selection and 20 occurred in the clinical strain despite strong purifying selection at those loci. Candidate markers were in functional domains of the RNP complex (*n* = 19), the matrix protein (*n* = 4), on CDV glycoproteins (*n* = 5), and on the V protein (*n* = 1). We found mutations at two loci in common between sequences from three CDV outbreaks of clinical distemper in African lions; one in the signaling lymphocytic activation molecule receptor (SLAM)-binding region of the hemagglutinin protein and another in the catalytic center of phosphodiester bond formation on the large polymerase protein. These results suggest convergent evolution at these sites may have a functional role in clinical distemper outbreaks in African lions and uncover potential novel barriers to pathogenicity in this species.

## 1. Introduction

Canine distemper virus (CDV) is a notoriously promiscuous multi-host pathogen that is typically associated with domestic dogs but capable of infecting taxa from at least twenty-two families across five orders [1]. CDV is the etiological agent of distemper disease, which can cause high morbidity and mortality and is among the most infectious diseases of mammals [2]. However, the clinical outcome of CDV spillover into alternative host species varies from no overt clinical signs (e.g., domestic cat, *Felis catus* [3]) to up to 100% mortality (domestic ferret, *Mustela putorius furo* [4]). Furthermore, within an alternative host species CDV infection outcomes may vary dramatically from no apparent impacts on individual or population health, to severe disease and mortality (African lions, *Panthera leo* [5] African wild dogs, *Lycaon pictus* [6,7]). What explains the differences in cross-species pathogenicity, i.e., the ability of CDV infection to cause distemper following spillover in a novel host, is not well understood.

Canine distemper in a susceptible host occurs as a result of two successive infection stages. The first stage involves virus invasion of host lymphatic cells which is achieved when the CDV glycoprotein hemagglutinin (HA) recognizes and binds the host signaling lymphocytic activation molecule receptor (SLAM). Subsequently, CDV is amplified and spreads systemically to secondary lymphoid organs and massively suppresses the immune system [8]. Though the host is immunosuppressed and transiently febrile, it does not exhibit overt clinical signs of distemper (e.g., respiratory, enteric, and epidermal signs) [9]. In the second stage of infection the virus spreads to epithelial cells lining organs throughout the body of the host using nectin-4 receptors for cell entry [10]. Tissue damage and inflammation associated with epithelial cell infection leads to the characteristic clinical signs of distemper and viral shedding [10]. Importantly, immune cell infection (stage 1) is necessary but not sufficient to cause distemper and/or onward transmission. Epithelial cell infection (stage 2) is necessary for clinical disease and onward transmission but must be preceded by massive immune cell infection (stage 1).

As the initial site of host invasion, the biochemical compatibility between viruses and host cell receptors is often a major determinant of the susceptibility of a host to infection [11]. However, essential viral processes post-entry can limit the ability of a virus to cause clinical disease in a host. Within-host barriers to pathogenicity following cell entry can include the ability to evade host immune defenses (vesicular stomatitis virus [12]; rinderpest virus [13]), the ability to make viral proteins and replicate in the cell (influenza A [14]; deformed wing virus, [15]), intracellular trafficking (adeno-associated virus [16]), and the ability to exit the cell and spread to new hosts (influenza A [17]; H5N1 avian influenza virus [18]). Intrinsic differences between host species affecting any of these processes, in either or both lymphatic and epithelial cells, may need to be overcome to induce a disease state, i.e., distemper, in a novel host. 

The role of viral evolution in driving CDV emergence in alternative hosts has been reported in observational studies and investigated experimentally. These studies find that specific amino acid changes at sites under selection in the SLAM-binding region of the viral HA are associated with distemper emergence in non-dog hosts [19,20]. While multiple independent reports support this correlation, others do not find statistical support for this association in alternative host taxa, namely felids [21]. Thus, though identified HA mutations may optimize infectivity in a new host, they are not necessary and sufficient for CDV emergence in novel hosts. 

African lions (*Panthera leo*) are susceptible to CDV infection; however, the pathogenicity of CDV infection in lions is apparently variable. For example, serological evidence suggests that CDV infection in captive African lion populations without manifestation of distemper is not uncommon [22,23]. Similarly, CDV seropositivity has been reported in wild lion populations without observed clinical signs of distemper or mortality [5,24,25,26,27]. By contrast, other CDV outbreaks in captive and wild lion populations have caused acute clinical signs of distemper and high distemper-specific mortality (captive [28,29]; wild [30,31,32]). Variability in CDV pathogenicity in lions was observed even within a single population exposed to recurrent CDV outbreaks over a 30-year period [5]. Specifically, more than 30% of the Serengeti lion population died or disappeared during one CDV outbreak in 1993/1994 [30], while serological evidence suggested that the Serengeti lion population sustained CDV exposure without overt distemper or mortality on at least 4 other occasions [5].

Co-infection with other pathogens can play a significant role in morbidity and mortality in CDV-infected hosts, given the massive toll that infection has on the immune system. Indeed, co-infection with Babesia was associated with higher mortality in lions during the 1993/1994 outbreak in the Serengeti lion population and in a second high-mortality CDV outbreak in the nearby Ngorongoro Crater in 2001 [5]. Significantly higher loads of Babesia were documented in lions during these outbreaks versus periods when CDV outbreaks did not coincide with mortality in lions, and in 1993/1994 Babesia load correlated with higher mortality among prides [5]. Interestingly no clinical signs or pathology indicative of distemper were observed in the Ngorongoro lions, despite high CDV titers indicating recent exposure [5]. While co-infection can explain differences in demographic outcomes of CDV infection, i.e., mortality, what drives differences in clinical presentation of distemper disease, i.e., pathogenicity, is not currently well understood.

In the present work we identify viral genotypes correlated with clinical phenotypes of CDV in African lions, to explore the hypothesis that viral evolution facilitates cross-species pathogenicity in this species. Whole CDV genome sequences were generated representing clinical and apparently subclinical CDV strains in lions in East Africa. We report 28 candidate markers, single-nucleotide polymorphisms (SNPs) that differentiate clinical and subclinical strains in lions at loci experiencing selection pressure. Mutations at two candidate markers were found in common between three independent CDV outbreaks that were characterized by distemper clinical signs and distemper-associated mortality in African lions; one in the SLAM-binding region of the HA and another in a highly conserved region of the RNA-dependent RNA polymerase critical for polymerization during viral transcription and genome replication. Our results suggest a role for viral evolution in CDV pathogenicity in a novel host and reveal new insights into potential mechanisms for surmounting host barriers in this virus.

## 2. Results

### 2.1. Sequences

A total of 21 CDV near-whole-genome sequences (WGS) (Table S1) and a single partial CDV H gene sequence (717 bp; GenBank Accession number MT943490) were generated, representing CDV strains associated with clinical and subclinical outcomes in African lions. Additionally, all available CDV WGS at the time of this study (*n* = 98) were sourced from public archives for subsequent analyses (Table S2). The dataset was pruned to include only one WGS from an infected individual.

### 2.2. SNPs Differentiating Clinical and Subclinical CDV Strains in East Africa

To identify loci that could be associated with CDV pathogenicity in lions we identified single-nucleotide polymorphisms (SNPs) differentiating strains in circulation in East Africa associated with distemper in lions (clinical) from those that were not associated with overt clinical signs (subclinical). The clinical group comprised 17 sequences during a single outbreak during which lions exhibited distemper clinical signs and high mortality (1993–1994). The subclinical group comprised eight sequences collected over three periods of peak CDV infection in lions that occurred in Laikipia, Kenya in 2000 and in the Serengeti ecoregion 2006–2007 and 2011, during which no CDV clinical signs or increased mortality was observed in lions.

In total, SNPs at 49 loci in the CDV genome differentiating clinical and subclinical strains were identified based on a multiple sequence alignment (Table 1). SNPs between the strains occurred across all six structural genes: N (*n* = 6), P (*n* = 6), M (*n* = 4), F (*n* = 7), H (*n* = 6), and L (*n* = 17), and in intergenic untranslated regions (UTRs) (*n* = 3). Three mutations occurred in the overlapping open reading frames (ORF) of P, V, and C. Thus, taking translation into account 54 total SNPs were observed. Nonsynonymous substitutions accounted for 22 (40.7%) of the SNPs, 28 (51.8%) were synonymous, and three occurred in UTRs (5.6%). The 54 SNPs observed between clinical and subclinical strains reveal loci that may have potential clinical relevance. If mutation at a locus improves CDV fitness in a given host, then we expect those sites to experience detectable selection pressures.

### 2.3. Evolutionary Processes Acting on CDV Genomes

All available CDV WGS spanning CDV geographic and host distribution, including those sequenced for this study and publicly available WGS, were analyzed to evaluate the potential role of recombination in shaping genetic diversity and to characterize selection pressures acting on individual loci across the CDV genome. Here we identify candidate markers of CDV pathogenicity in lions as loci that differentiated clinical and subclinical strains in East Africa and at which positive, negative, or episodic diversifying selection was detected.

Recombination analyses detected 16 unique putative recombination events in 11 sequences (Table 2) from 119 CDV WGS analyzed. Nine of these putatively recombinant sequences have been described elsewhere [33,34,35,36]. Five recombination events were detected in a single raccoon dog (*Nyctereutes procyonoides*, Canidae) genome sampled in China in 2013 (GenBank Accession KJ994343). No putative recombinant CDV strains were associated with pathogenicity in lions. Putative recombinant strains were removed from selection analyses.

Site-by-site selection analyses of 108 concatenated CDV protein coding sequences (CDS) representing all gene products using single-likelihood ancestor counting (SLAC), fixed effects likelihood (FEL), and mixed effects model of evolution (MEME) methods on the Datamonkey Adaptive Evolution Server (https://www.datamonkey.org/), identified 118 sites with pervasive positive or episodic diversifying selection (*p* ≤ 0.1) (Table S3), while 1,405 sites showed evidence of purifying (negative) selection operating across the phylogeny (*p* ≤ 0.1) with SLAC and FEL (Table S4).

Of the 54 loci differentiating clinical and subclinical strains in East Africa, 29 (53.7%) were subject to selection (Table 3) according to the site-by-site selection analyses of all available whole CDV CDS. Pervasive positive or episodic diversifying selection accounted for ten of these sites (34.5%) occurring on the N gene (residues 451 and 466), the P and V genes (residue 280), the M gene (residues 9), the H gene (residues 519 and 549), and the L gene (residues 93, 133 and 1402). Twenty sites (71.4%) were classified by SLAC and FEL algorithms as experiencing negative selection, i.e., an excess of synonymous substitutions was detected. These occurred on all CDV gene products, except for C. Both negative and episodic diversifying selection were detected at a single site (L93). At residue 1619 on the large polymerase (L) gene, the clinical and subclinical strains differed because the subclinical strain had a unique mutation, while all other sequences had a conserved nucleotide at that site. Thus, excluding site L1619 and removing the duplicate L93 detection, we identified 28 candidate markers of CDV pathogenicity in African lions.

### 2.4. Phylogenetic Analyses and Distribution of Mutations at Candidate Markers

**Figure 1:**
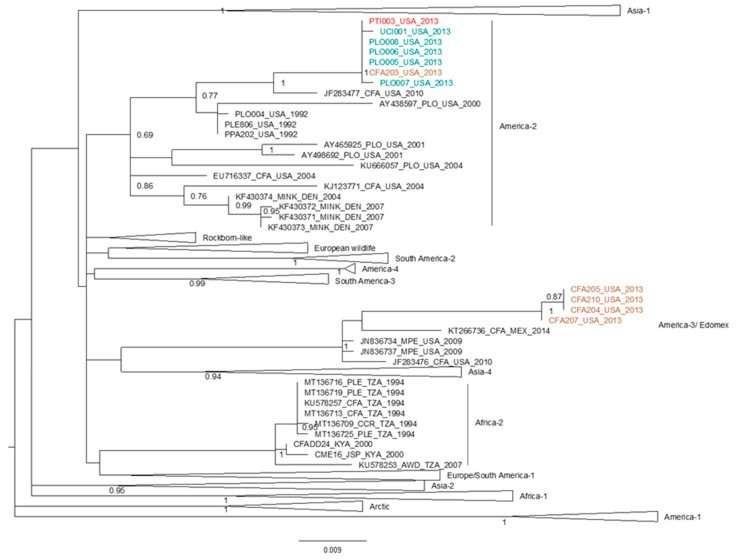
Maximum likelihood (ML) phylogenetic tree of partial CDV H gene sequences (717 bp) shows the relationship of a CDV sequence from a tiger (in red) sampled at captive facility in North America in 2013 where African lions displayed clinical signs of distemper, with respect to other CDV strains from across the global distribution. Domestic dogs and wild carnivores sampled contemporaneously in the region of the facility are labeled in brown and green, respectively. Bootstrap support is indicated at nodes (BS = 1000). Global CDV lineages are indicated to the right of clades.

Phylogenetic analysis of partial H gene sequences (717 bp) revealed that two genetically distant CDV lineages were circulating in or near Dallas, Texas in 2013, when a clinical outbreak of distemper impacted African lions at a big cat rescue facility (Figure 1). A clade formed by CDV sequences from a tiger, five wild carnivores, and one domestic dog are closely related to sequences identified as America-5 which is a subclade of the America-2 strain, which infected big cats in North America in 1991–1992. CDV sequences from four domestic dogs sampled contemporaneously in the Dallas area (Texas) are distinct from the strain associated with the outbreak in big cats, and cluster with America-3 sequences, which are widespread in North and Central America [37]. The partial CDV-H sequence from the tiger was identical to wild mesocarnivore sequences suggesting that the outbreak at the big cat facility originated in wild carnivores. Given this subgenomic sequence identity, WGS from these mesocarnivores were used as a proxy for the CDV sequence causing distemper in African lions in Texas in 2013 in the whole-genome phylogenetic analysis.

**Figure 2:**
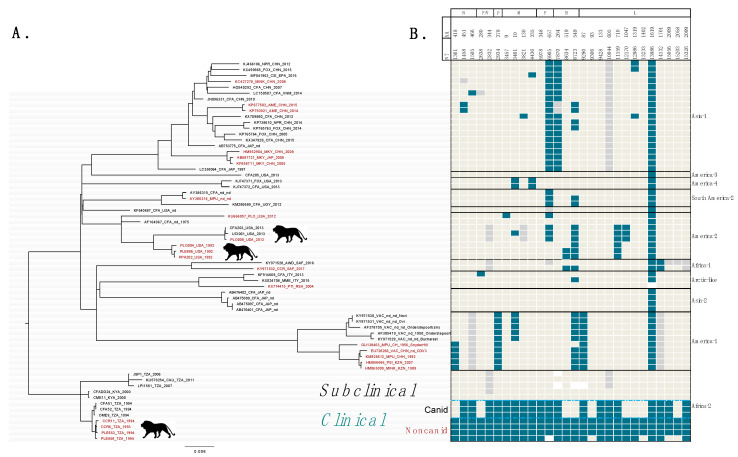
Phylogenetic relationships among globally distributed canine distemper virus sequences and distribution of candidate markers of CDV pathogenicity in African lions. (**A**) Maximum likelihood (ML) phylogenetic tree of near-whole CDV genomes (15,584 bp) generated in this study and publicly available sequences collected globally. ML tree is rooted on the Africa-2 clade. Black circles at tree tips indicate sequences generated for this study. Font color of sequences, black, red, or grey indicates canid, non-canid, or vaccine origin, respectively. Clades implicated in distemper clinical signs in African lions indicated by lion icon. Bootstrap support of 500 replicates shown at nodes (**B**) SNPs at candidate loci are mapped to tips of the phylogeny representing globally distributed CDV strains. The genomic position of SNPs at candidate markers are shown in top row. Font color indicates whether overall selection at site is episodic diversifying (orange) or purifying (black). Teal blocks indicate identity with the African lion clinical strain circa Serengeti 1993–1994, taupe blocks = conserved allele, grey = alternative allele, white = no data.

Phylogenetic analysis of 65 CDV WGS indicated that 10 previously recognized global clades were represented in our data (Figure 2A). Sequences from the three outbreaks causing clinical distemper in African lions fall into 2 global lineages, Africa-2 and America-2. All sequences from East Africa occur in Africa-2. Both North American outbreaks in captive African lions, in 1992 and 2013, are of the America-2 global lineage and are 95% similar in the H gene, though they occurred 21 years apart.

Mapping SNPs at candidate markers to the tips of the CDV WGS phylogeny representing global diversity revealed that (1) nine of 28 loci segregating clinical and subclinical East African strains also segregated canids and non-canids during the clinical East African outbreak (including P/V280 which accounts for one site on two genes), (2) six candidate genetic markers (excluding site 13,386) are shared between the 1994 Serengeti outbreak and a strain causing clinical distemper infection in lions and other big cats in North America in 2013, and (3) mutations at two candidate loci were found in all documented outbreaks of distemper in lions for which there is WGS data (Figure 2B).

In East Africa, by definition, the consensus sequence of clinical strains differs from subclinical strains at all 28 candidate markers. However, nine of the 28 candidate markers differentiating clinical and subclinical East African strains also segregated the canid and non-canid sequences within the 1993–1994 outbreak, with three exceptions involving a single lion. Lion PLE658 shares the same SNP at 3 sites on the L gene as the sequences found in canids during the outbreak, shares SNPs at 23 candidate markers with other lions and non-canids during the outbreak, and shares one SNP with the subclinical genotype found in East African (no data is available for one candidate site from PLE-658) (Figure 2B).

Mutations at six candidate genetic markers (excluding site 13,386) are shared between the clinical CDV strain (of Africa-2) and a strain causing clinical distemper infection in lions and other big cats in North America in 2013. A third outbreak in African lions and other big cats in 1992–1993 shares two of these. CDV strains from both clinical outbreaks in lions outside of Africa belong to the America-2 global lineage. Thus, in the search for a genetic signature of pathogenicity, we found at least two and up to six loci associated with independent, clinical outbreaks in lions. Furthermore, candidate markers of pathogenicity in African lions identified in this study were also observed in other novel host species and vaccine strains across the phylogeny.

## 3. Discussion

In this study we sought to identify candidate viral markers associated with pathogenicity of CDV in African lions to gain a better understanding of potential barriers to clinical spillover in this species. Fifty-four SNPs differentiated genomes from CDV strains in circulation in East Africa during CDV infection peaks that were not apparently pathogenic in lions from genomes of CDV sequences which were pathogenic in lions. Of these, ten SNPs were at loci experiencing pervasive positive or episodic diversifying selection, and twenty occurred at loci despite the strong purifying selection acting on these sites. Most of these mutations mapped to functional domains of the RNP (polymerase) complex and matrix protein, implicating the processes of transcription and replication, and viral budding as potential barriers to clinical CDV spillover in lions. We investigated whether the mutations correlated with pathogenicity in lions in East Africa were involved in other CDV outbreaks affecting African lions in captive populations in North America. We found mutations at two loci in common between all available sequences from CDV outbreaks of clinical distemper in African lions—one in the SLAM-binding region of the hemagglutinin protein (Y549H) and a synonymous mutation in the catalytic center of phosphodiester bond formation on the large protein (710). These results suggest convergent evolution at these sites may have occurred allowing CDV to surmount species barriers, and potentially uncovered a novel mechanism of CDV pathogenicity in African lions.

### 3.1. Candidate Loci Associated with CDV Pathogenicity in Lions in East Africa

Our results support the hypothesis that viral genetic factors are associated with CDV pathogenicity in African lions. We found that 28 SNPs at loci under selection were associated with clinical strains of CDV in African lion populations in East Africa. During the catastrophic 1994 distemper outbreak in lions (samples from which comprise the clinical group), 13 mutations separated sequences from canid and non-canid species [38]. Mutations at nine of these 13 sites overlap the 28 candidate markers differentiating clinical and subclinical strains in East Africa. This finding is consistent with previous work suggesting that mutations within 1994 are explained by adaptive evolution to infection of non-canids at these sites [39] and supports the role of viral genetic factors in explaining clinical spillover in lions in East Africa. 

Although mutations at candidate markers identified here are correlated with clinical outcome of CDV in East Africa, it is not possible to determine whether these mutations have a functional role in clinical infection in lions with our observational data. One lion, PLE658, with an intermediate haplotype between canid and non-canid strains sampled during the 1993–1994 Serengeti outbreak, provides anecdotal evidence to this end. Although the sequence is ancestral to all other non-canid sequences, as determined by previous analyses of 21 time-stamped CDV sequences from non-canids and canids sampled between December 1993 and December 1994, this sequence was sampled from a lion found dead in the center of the study area during the middle of the outbreak [38]. Furthermore, histopathology suggestive of CDV infection was found in lymphatic tissue (spleen) while within normal limits in the lymph nodes and liver tissues and the absence of obvious lung lesions were noted on field necropsy. While it is impossible to retrospectively determine whether this individual had clinical distemper or not given the scant evidence, no pathology was found to support a clinical diagnosis. If this lion were subclinical it would implicate specific mutations on the large polymerase protein (L-93, L-1402, L-2009 and L-2058) as particularly important for determining clinical infection in lions during this outbreak. Regardless of clinical presentation, this intermediate haplotype was not detected elsewhere in space or time during the outbreak, suggesting that either it was not widespread or not easily detected. 

### 3.2. SNPs at Candidate Loci Occur in Multiple, Independent Distemper Outbreaks in Lions

Our data identified candidate loci associated with clinical outcome in African lions within a single CDV lineage (Africa-2) and provided a basis for testing the hypothesis that a common mutational signature (i.e., genotype) explains the CDV pathogenicity in African lion populations (i.e., phenotype) infected with other CDV lineages. Assessing the distribution of mutations at the 28 identified candidate loci over all available WGS revealed mutations at two sites associated with all outbreaks of clinical distemper in African lions in our dataset. These are a tyrosine to a histidine at residue 549 (Y549H) on the hemagglutinin protein and a synonymous mutation at residue 710 on the large protein.

Our finding of Y549H in African lions is consistent with that of McCarthy et al. (2007), who found positive selection and an association of histidine at this site in non-dog hosts [19]. Multiple subsequent reports have supported this pattern, including Nikolin et al. [20] which revisited the question of the H-549 association using a larger sample size and narrowed the association to non-canid hosts, i.e., wild canid hosts were more likely to bear a tyrosine like their domestic counterparts. Furthermore, CDV-H proteins from a canid strain experimentally mutated to bear a histidine at 549 significantly improved syncytia formation over the non-mutated protein bearing tyrosine in Vero cell lines expressing lion and cat SLAM cell receptors, supporting the hypothesis that this mutation plays a functional role in CDV host tropism [40]. Interestingly, a similar in vitro study found a possible additive interaction between 549H and another amino acid substitution in the SLAM-binding region, arginine to isoleucine at residue 519, on syncytia formation in Vero cells expressing lion SLAM [39]. Our analysis detected episodic diversifying selection at R519I in non-canid hosts in two of the three outbreaks of clinical distemper examined (Serengeti 1993-1994 and North America 1992, consistent with previous findings [40]) as well as in an infected spotted hyena in South Africa in 2017. Importantly, in 1992 both sequences from big cats had 519I, while the only raccoon tested contemporaneously had 519R. Unfortunately, we were unable to determine if the virus infecting big cats in 2013 had this mutation because we failed to generate a WGS from a felid at this time. The wild carnivores sampled contemporaneously in 2013 did not have the mutation, and arginine is well conserved at this site across geographic and host ranges.

Despite the observed associations of the Y549H mutation with non-dog hosts, previous work showed no clear association of histidine at 549 with sequences obtained from Felid species [21]. Our results suggest that from the Family Felidae, Y549H is at least correlated with the presentation of clinical distemper in African lions, though the sample size of three outbreaks is small. The discrepancy between finding Y549H in some but not all non-canid hosts might be explained by the dominant cross-species pathways of transmission during an outbreak. The residue at site 549 in a sequence may be driven by whichever species is fueling an outbreak, i.e., the reservoir. Thus, if the reservoir is a canid species, then tyrosine might be expected; if the reservoir is not a canid, then histidine might be expected. To that end, tigers infected in Japan, China and Russia, where the reservoir or source might be expected to be a raccoon dog (Canidae) were 549Y [23,41,42], while tigers infected in both North American outbreaks putatively fueled by raccoons (Procyonidae) had 549H [28].

The mutation at residue 710 occurs in the 223-amino acid, conserved region (CR) of the L protein, CRIII. This functional domain is host to the catalytic center of phosphodiester bond formation, i.e., where polymerization occurs during transcription and replication [43]. Although this mutation does not confer an amino acid substitution, positive selection at third codon positions is well documented in viruses and other taxa [44,45,46]. That this mutation is shared by all pathogenic strains in lions, and it is conserved in all other available CDV sequences from all hosts and global lineages, suggests convergent evolution and supports the hypothesis that this mutation confers a fitness advantage to CDV in African lions. 

Our phylogenetic results indicate that the 1992 and 2013 North American outbreaks were associated with the same global lineage, America-2. Thus, mutations at common sites between these two outbreaks may be identical by descent, rather than by parallel evolution. Nevertheless, that the same global lineage was responsible for two of three well-documented outbreaks in lions supports the hypothesis that viral genetic factors are associated with lethal CDV spillover in lions. Furthermore, this also suggests that America-2 could have an intrinsic ability to cause lethal infection in big cats.

The observation of two mutations in common across all lion outbreaks may be conservative. The 1994 East Africa and 2013 North America outbreaks shared mutations at six loci, while the 1992 North America strain shared two of these. Sequences from 1994 and 2013 were sequenced from clinical samples, whereas the sequences from 1992 were isolated and passaged in various types of canine cells and canine cell lines. Thus, any mutations that conferred an advantage in lion cells may have reverted or been lost in adaptation to cell culture on canine cells.

In addition to America-2, adaptive mutations found in the clinical East African group were shared with CDV sequences in other lineages, the majority of mutations at shared sites (78.8%) were found in non-dog host species and/or vaccines. CDV vaccines were historically made by repeat passaging of a clinical isolate in alternative host species in vivo and in alternative host cells in culture until the isolate acquired so many mutations that it was attenuated in domestic dogs. Thus, the distribution of mutations in common with the clinical strain in a diversity of species (including vaccines) suggests that some of the mutations at candidate loci in our analysis may not be specific to lion infections, but rather related to the adaptation to alternative host species more generally. This is consistent with the thesis of Nikolin et al. [40] that CDV in non-dog hosts acquire “generalist” mutations. Sequences from global lineages in our dataset comprised only of domestic dogs, specifically America-3 and Asia-2, do not share any SNPs correlated with clinical outcome in lions at sites that are under selective pressure.

### 3.3. Mutations at Candidate Loci Occur Primarily in the RNP Complex and Matrix Protein

While mutations at the candidate loci identified were not found to be in common across all outbreaks in lions, these loci may have functional relevance influencing infection outcomes. Experimentally testing the relevance of candidate loci identified here was outside of the scope of this study. However, the functional domains of CDV and other related Paramyxoviruses are well characterized through previous studies that manipulate viral sequence and observe the resulting change in phenotypes.

**Figure 3:**
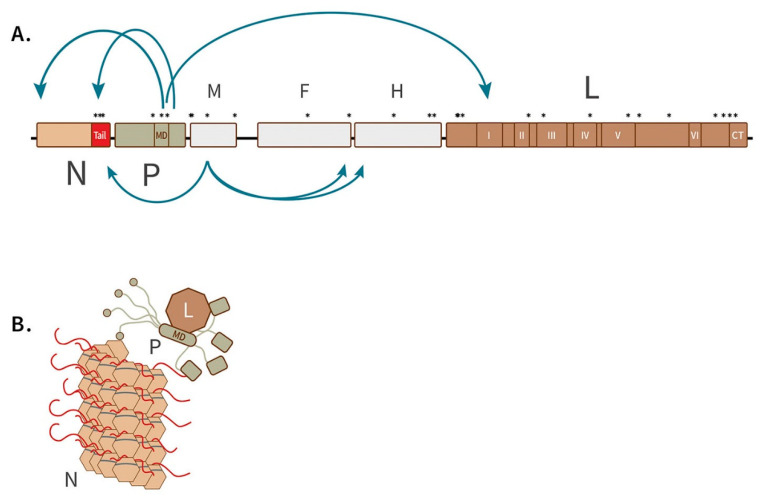
The genome and encoded structural proteins of the RNP complex of canine distemper virus. (**A**). Schematic of CDV genome organization showing location of candidate markers of pathogenicity in African lions (asterisks). Each of six structural genes (N, P, M, F, H, and L) represented by a box is connected by a black line indicating intergenic regions of the genome. N, P, and L are subdivided by smaller labeled boxes showing the locations of highly conserved regions with known functions. Arrows indicate long-range interactions of the encoded proteins necessary to carry out the functions of the virus in the host cell. Host cell co-factors not shown. (**B**). Model of the RNP complex binding to the nucleocapsid which encapsidates the viral RNA (dark grey ribbon) initiating polymerization for transcription or replication. Colors correspond to genome in A. Figure adapted from [47].

The majority of the 28 candidate loci correlated with pathogenicity in African lions were located in functional domains of the RNP complex essential for efficient viral transcription and replication (*n* = 19), and on the matrix protein critical for viral spread (*n* = 4). Mutations in these functional domains suggest that these processes may present barriers to clinical spillover in African lions. All mutations identified in this study are mapped in relation to functional domains on a model of the CDV genome in Figure 3A. 

The RNP is comprised of three viral proteins, the N, P and L (illustrated in Figure 3B—adapted from [47]) and host cell cofactors (not shown). The RNP is important because it synthesizes viral mRNA and makes copies of the viral RNA genome. The nucleocapsid (N) encapsidates the viral RNA and protects it from degradation and detection by the host innate immune system. The large protein (L) contains the catalytic functions of the RNA-dependent RNA polymerase (RdRp) necessary for mRNA transcription and viral replication. The phosphoprotein is an essential cofactor in polymerase activity, responsible for chaperoning the polymerase (L) to the nucleocapsid (N), positioning it at the 3’ promoter of the encapsidated viral RNA, and preventing it from falling off during polymerization [48].

On N, three candidate loci in our data occurred in the 125-aa N-tail domain (red in Figure 3A,B). Research on Paramyxoviruses suggests that this short amino acid chain protruding from the nucleocapsid is involved in (1) binding the P, which, in turn, recruits the L for transcription and replication, (2) binding the matrix protein for translocation of the RdRp to the cell surface during egress [49], (3) binding a host cell receptor that leads to suppression of immune cell proliferation in the host [50], and (4) facilitating access to the RNA genome for RdRp, and (5) regulating RdRp activity and as such determining pathogenicity [51,52]. Mutations in the N-tail thus may affect three vital processes: efficient transcription/replication, the massive suppression of the host immune system, and onward spread.

Three candidate loci occur on the phosphoprotein, P. Two mutations on the P gene fall in the P multimerization domain PMD, a short domain (of 66 residues) that research suggests both binds L and is pivotal in its correct positioning for polymerization initiation [53,54]. Thus, these mutations may optimize recruitment of the polymerase for transcription/replication in a novel host. The remaining P mutation, P-280, also occurs in the V protein open reading frame, which is not a part of the RNP. On the V gene, mutation V-280 occurs immediately following a highly conserved, essential chain of 47 amino acids that interrupts host innate immunity. A V-deficient CDV caused limited viremia, transient leukopenia, and mild symptoms in experimentally infected ferrets [55].

On the L protein, six conserved domains (common to all Morbilliviruses) are associated with enzymatic activity of mRNA transcription and virus replication, e.g., catalyzing polymerization and mRNA capping, methylation, and polyadenylation (essential for translation and evading host innate immunity) [56]. In addition, there are intrinsically disordered sites having no secondary structure and a connector domain [57]. Six of eleven mutations associated with the clinical strain occur in discrete and specific enzymatic domains on the L protein (Table 3). These occur in CRII, CRIII, CRIV, CRV, and the C-terminus. The others occur in connector and linker domains and could be involved in secondary structure and proper RNA folding facilitating interaction between the functional domains [58]. Mutations on the L gene may optimize the processes of mRNA transcription and viral replication, and indirectly contribute to host immune evasion, which requires that capped, polyadenylated mRNAs be produced.

Interestingly, the gene with the second most mutations associated with the clinical strain was the matrix protein (M). The M protein is essential for viral assembly and egress from the infected host cell, i.e., onward transmission. M interacts with the cytoplasmic tails of the virus glycoproteins, F and H, the N-tail of N, and host cellular actin filaments to assemble virus particles at the cell surface [59]. The role of matrix protein is especially important in polarized epithelial cells of Measles virus, where it directs the viral assembly and egress from the apical side, ensuring onward spread of the virus by releasing it into the respiratory, urinary, and gastrointestinal tract [60]. The matrix protein can regulate cell fusion, which is an important process in the delicate balancing act between productive cell infection and host immune evasion. Four mutations were found on M that differentiated the clinical and subclinical strains in East Africa. However, no M mutations occurred between canid and non-canid strains within the clinical group. 

Previous work showed that, in vaccine strains of CDV, six mutations occur on the M gene as compared to the virulent wildtype, interrupting its function [61]. Recombinant viruses replacing the vaccine M into a wildtype strain resulted in complete attenuation in vivo. These findings demonstrate that a virus with a defective M (as found in vaccines) can replicate in host cells, initiating the host immune response and leading to immunity. Thus, if a wildtype CDV M protein cannot interact with the cell cytoskeleton or other host cofactors of a novel host effectively, then a host exposed to wildtype virus may seroconvert without clinical infection. 

Adaptive mutations on the two glycoproteins of CDV (F and H) associated with clinical spillover of CDV in African lions in East Africa were fewer than expected, with two mutations on the fusion gene (F) and three on the hemagglutinin (H). The H protein is responsible for recognizing and binding the host cell, and works in concert with proximal F proteins to fuse the cell and viral membranes. Because this process is required to initiate infection, and the SLAM binding region has variable affinity to SLAM receptors from different host species, genetic diversity in the SLAM binding region has been considered the primary determinant of CDV host tropism. Indeed, SLAM binding defines susceptibility and host tropism; however, it does not determine host pathogenicity. For example, CDV recognizes and binds domestic pig and cat SLAM, and replicates in SLAM-positive cells, but does not cause clinical disease [62,63]. Considering the widespread occurrence of CDV seroconversion in taxa ranging from elephants to deer and all families of carnivores [1], the ability to bind SLAM does not seem to be the limiting factor in cross-species pathogenicity.

## 4. Conclusions

Identifying genetic markers of clinical outcome in African lions is a useful predictive tool and can improve surveillance when (1) the mutations are necessary and sufficient to cause disease, and (2) are present in the susceptible host community before spillover occurs, i.e., “off-the-shelf” [64]. Specifically, the risks of CDV infection in a population of conservation concern might be determined by screening sympatric, putative reservoir populations, e.g., domestic dogs, for CDV variants bearing the genetic signature of increased pathogenicity in lions. Our data do not address whether these conditions are met by the candidate loci we describe here. However, convergent evolution at two or more sites strongly suggests a functional role in clinical infection in lions. Thus, surveillance for these two mutations at a minimum in circulating CDV strains could beused as an early warning tool in proximity to populations of high conservation value. To this end, our analysis revealed that two well-documented outbreaks affecting African lions were caused by the America-2 global lineage. This lineage could be screened for and considered a risk to sensitive African lion populations.

The presence of a common genetic signature requisite for clinical spillover in African lions might be precluded if there exist multiple ways, i.e., mutations, to achieve the same phenotypic effect. This may be that different mutations or combinations of mutations in a certain functional domain will have the same effect on the process, or that mutations affecting different processes are sufficient to produce the same phenotype in vivo, i.e., a clinical outcome in lions. Identifying sites in the CDV genome under selective pressure in clinical outbreaks of CDV has an intrinsic value in highlighting viral processes that may pose a barrier to CDV spillover in lions and other species, even if a diagnostic signature is elusive.

The search for genetic signatures predicting clinical outcomes of disease exposure is the holy grail of those interested in predicting and preventing poor individual and population level outcomes of infection. To adequately test whether a pathogen genotype predicts phenotype in a given host necessitates experimental validation through mutagenesis and challenge experiments [64]. In this study, we use observational data and whole-genome sequences to look for candidate sites associated with canine distemper virus clinical outcomes in African lions and other novel hosts. The candidate loci identified here are critical for developing hypotheses to motivate the necessary microbiological experiments to better understand the evolutionary dynamics of cross-species pathogenicity in CDV.

## 5. Materials and Methods

### 5.1. Study Specimens

To assess whether adaptive changes in the CDV genome are associated with pathogenicity in lions, samples were acquired from animals involved in three independent CDV outbreaks causing observable symptoms of distemper in African lions. These included two outbreaks in captive populations of exotic big cats in North America in 1992 [28] and 2013 [65]. CDV WGS from a third outbreak causing distemper in the wild, well-observed lion population in the Serengeti Ecoregion in 1993–1994 [30] were published previously [38]. 

In 1992, 35 of 74 big cats at the Wildlife Waystation (California) showed clinical signs of distemper including respiratory, gastrointestinal, and neurological symptoms. Of these, 17 died (48% mortality in big cats given distemper onset), including seven African lions. Virus isolates on canine blood lymphocytes from one African lion, one common leopard (*Panthera pardus*), and one free-ranging raccoon (*Procyon lotor*) were stored at −80 °C and acquired for this study. Virus isolation methods are described in [28].

In May and June of 2013, 21 captive big cats at a facility in Texas developed distemper disease, including five of eight resident lions [66]. In total, 12 big cats experiencing distemper symptoms died or were euthanized, including one African lion. Clinical signs in African lions included anorexia, loose stools (*n* = 4), and seizures and death (*n* = 1). Samples from lions at the facility were not available, though a CDV-positive urine sample (by RT-qPCR) from a recovered tiger (*Panthera tigris*) from the same facility was obtained. Wild and domestic carnivores were sampled from the area contemporaneously. Tissue samples from wild rabies-suspect, rabies-negative mesocarnivores were acquired from the Texas Department of State Health Services including raccoons (*n* = 4) and a gray fox (*Urocyon cinereoargenteus*, *n* = 1) collected between April and May 2013. CDV-positive cDNA was obtained from IDEXX Laboratories, Inc. from domestic dogs *(Canis lupus familiaris*, *n* = 5) that were sampled between November 2012 and December 2013 by Dallas Animal Services.

Serological studies in the Serengeti ecoregion and in nearby Laikipia, Kenya, indicate CDV infection peaks in lions and other carnivores with no observed distemper disease or distemper-specific mortality in African lions [5,26]. Samples from domestic and wild carnivores collected in the Serengeti and Laikipia during these peaks were included in this study. Samples from a jackal (unknown species, *n* = 1) and African wild dog (*Lycaon pictus*, *n* = 1) were collected in the Serengeti ecoregion in 2006 and 2007, respectively. Black-backed jackals (*Canis mesomelas*, *n* = 3) and domestic dogs (*n* = 2) were sampled at a ranch in Laikipia, Kenya in 2000 [26]. One CDV outbreak in the Serengeti ecoregion did result in distemper and distemper-associated mortality leading to a > 30% population decline. CDV WGS from multiple hosts (*n* = 21) were published previously and include domestic dogs, bat-eared foxes (*Otocyon megalotis*), spotted hyenas (*Crocuta crocuta*), and African lions.

### 5.2. Sequencing

#### 5.2.1. Whole-Genome Sequencing

Amplicon-based deep sequencing was used to generate near whole-genome sequences following previously described methods [38]. All sequences generated in this study were deposited to GenBank (GenBank Accession numbers Table S1).

#### 5.2.2. RT-PCR and Sanger Sequencing

A partial H gene sequence (717 bp) was generated from a tiger that initially suffered loss of appetite, loose stools, and seizures, apparently recovered over 10 months, then suddenly and rapidly declined with undiagnosed neurologic symptoms and died. Viral RNA was extracted from a urine specimen from this tiger using the QIAamp Viral RNA Mini Kit (Germantown, MD, USA) according to manufacturer’s instructions. A one-step RT-PCR reaction using previously published primers was performed using SSIII One-Step RT-PCR with Platinum Taq (Carlsbad, CA, USA). The RT-PCR reaction conditions were as follows: 10 min at 55 °C, followed by 2 min at 94 °C, then 40 cycles of 30 s at 94 °C, and 30 s at 48 °C, and 2 min at 68 °C. Each reaction used 2-μL total RNA extract in a 50-μL volume reaction. Oligonucleotide concentrations were used according to the manufacturer’s instructions. Sequences were generated using the Applied Biosystems Automated 3730xl DNA Analyzer with BigDyeTerminator chemistry.

### 5.3. Identifying Loci Differentiating Clinical and Subclinical CDV Strains in East Africa

To identify loci that may be associated with the clinical outcome of CDV in African lions, we first identified mutations differentiating the circulating CDV strains in East Africa with apparently different clinical outcomes in lions between 1992 and 2011. If CDV evolution has a functional role in cross-species pathogenicity, then sequences collected during an outbreak with observable distemper symptoms in lions (hereafter “clinical”) will differ from sequences collected during infection peaks with no observed distemper symptoms in lions (hereafter “subclinical”) at functional domains. The East African sequence data were divided into two groups: clinical and subclinical. The clinical group comprised all CDV WGS collected during the outbreak in the Serengeti Ecological Region between December 1993 and December 1994, including domestic dogs and wild carnivores (*n* = 21). During this outbreak, CDV sequences collected from canid hosts (bat-eared fox and domestic dog) differed from sequences from non-canid hosts (African lion and spotted hyena) by 13 SNPs [38]. It is hypothesized that some of these differences could have a functional role in host tropism; however, it is unknown whether the “canid” haplotype would cause distemper symptoms in the non-canid group. Thus, both canid and non-canid sequences were considered clinical here. The subclinical group comprised samples from African wild dogs, black-backed jackals, golden jackals, unidentified jackal species, and domestic dogs collected from 2000–2011. Notably, the subclinical group contained only canid species because no non-canid species were observed with clinical signs during this period. Consensus genotypes of the groups were compared in a multiple sequence alignment performed using the Multiple Sequence Comparison by Log-Expectation, MUSCLE, algorithm in Molecular Evolutionary Genetics Analysis version 7.0 (MEGA7) for bigger datasets [67]. If a mutation differentiated the two groups, that locus was considered a potential marker of pathogenicity in lions. 

### 5.4. Evolutionary Analyses

CDV WGS were screened for recombination using a suite of algorithms available in Recombination Detection Program (RDP4) software [68], including Recombination Detection Program (RDP) [69], GENECONV [70], BOOTSCAN [71], Max-Chi [72], CHIMAERA [73] and SISCAN [74]. All tests employed a cutoff of *p* ≤ 0.05 and a Bonferroni correction. WGS with significant evidence of recombination events in at least five of the seven methods were considered putative recombinants.

Pervasive positive and negative selection, and episodic diversifying selection were assessed on a site-by-site basis, i.e., at each codon, using a counting method, SLAC, a fixed effects likelihood method, FEL, [75], and a mixed effects model, MEME [76]. Tests were performed in the HyPhy software [77] implemented on the Datamonkey web server [78]. Putative recombinant sequences were not included in the selection analyses because they cannot be described by a single phylogenetic history and the selection analyses are tree-based. A *p*-value cutoff of *p* ≤ 0.10 was used to determine significance in all analyses. This cutoff was used because the null model used for comparison in these methods assumes neutral evolution, which is likely to be violated by RNA virus evolution, which is dominated by purifying selection. Selection acting on mutations at intergenic sites was not investigated because the selection detection methods used only evaluate protein-coding sequence.

### 5.5. Phylogenetic Analysis

High-quality DNA for whole-genome sequencing was not available from any big cats infected during the outbreak at the North American facility in 2013; however, a partial H gene sequence was obtained from a recovered tiger. In addition, several WGS were generated from CDV strains in circulation in wild and domestic carnivores in the vicinity in the same year. To determine if these contemporaneous WGS sequences could be used as a proxy for the CDV strain causing the outbreak in the big cat population, a phylogenetic analysis of partial H gene sequences, including the contemporaneously collected wild and domestic carnivores, the recovered tiger, and publicly available partial H gene sequences from across the global distribution of CDV, was performed. Tests for the best-fit model of nucleotide substitution were implemented in jModelTest [79] and the Maximum Likelihood method was used to reconstruct the phylogenetic relationships of the partial H gene sequence in MEGA [67].

To assess which candidate genetic markers were associated with discrete outbreaks of clinical distemper in lions and whether the strains causing these outbreaks were genetically independent, we performed phylogenetic analyses of CDV WGS using the methods above and mapped the mutations to the tips of the branches. The complete CDV WGS dataset was pruned to improve visualization by including only one sequence per species, per country of origin, per year. The frequency and distribution of mutations at candidate loci in host species and in space were visualized by mapping them to the tips of the phylogeny.

## Figures and Tables

**Table 1:** Mutations at 49 loci in the canine distemper virus (CDV) genome that differentiate clinical and subclinical strains of CDV in circulation in East Africa between 1993 and 2011.

Position (nt)	Gene	Amino Acid/Gene	Subclinical	Clinical	Synonymous or Nonsynonymous	AA-Subclinical	AA-Clinical
132	N	9	A	G	non	T	A
470	N	121	A	G	syn	-	-
1361	N	418	T	C	syn	-	-
1382	N	425	T	C	syn	-	-
1458	N	451	T	C	non	F	L
1505	N	466	C	A	syn	-	-
1914 *	P/V/C	38/38/31	A	C	non/non/non	Q/Q/K	H/H/T
2200 *	P/V/C	134/134/126	G	A	non/non/syn	G/G/-	S/S/-
2638	P/V	280/280	A	G	non	K/K	E/R
2832	P	344	A/G	C	syn	-	-
2934	P	378	G	A	syn	-	-
3292	P	498	T	C	non	Y	H
3410	P-M UTR	n.a.	A	T	n.a.	-	-
3457	M	9	A	G	non	Q	R
3461	M	10	T	C	syn	-	-
3821	M	130	G	A	syn	-	-
4436	M	335	C	T	syn	-	-
4900	M-F UTR	n.a.	A	G	n.a.	-	-
5001	FSP	23	C	T	non	H	Y
5226	F	98	G	A	non	A	T
5978	F	348	T	C	syn	-	-
6058	F	375	G	A	non	R	Q
6694	F	587	T	C	non	V	A
6710	F	592	T	C	syn	-	-
6905	F	657	C	A	syn	-	-
6974	F-H UTR	n.a.	T	C	n.a.	-	-
7088	H	4	T	C	non	Y	H
7167	H	30	A	G	non	Q	R
7870	H	264	A	T	syn	-	-
8320	H	414	T	C	syn	-	-
8634	H	519	G	T	non	R	I
8723	H	549	T	C	non	Y	H
9210	L	61	A	C	non	M	L
9290	L	87	C	T	syn	-	-
9308	L	93	C	T	non	L	F
9428	L	133	T	C	syn	-	-
10844	L	605	T/C	G	syn	-	-
11159	L	710	C	T	syn	-	-
12170	L	1047	T	C	syn	-	-
12986	L	1319	T	C	syn	-	-
13142	L	1371	A	G	syn	-	-
13233	L	1402	A	C	non	-	-
13524	L	1499	A	G	non	I	V
13886	L	1619	C	T	syn	-	-
14132	L	1701	A	G	syn	-	-
15056	L	2009	T	C	syn	-	-
15185	L	2052	C	T	syn	-	-
15203	L	2058	A	C	syn	-	-
15326	L	2099	T	A	syn	-	-

* Mutation occurs in overlapping open reading frames.

**Table 2:** Recombination events detected in the alignment of 122 whole CDV genome sequences using seven algorithms in the Recombination Detection Program (RDP).

Acc. No.	#	Species	Country	Year	Clade	RDP pval	GC pval	BS pval	MC pval	Ch pval	SS pval	3Seq pval
AB462810	1 ^	CFA	Japan	nd	Asia-2	9.95 × 10^−28^	4.86 × 10^−26^	4.93 × 10^−23^	4.67 × 10^−8^	8.28 × 10^−8^	0.000173	1.32 × 10^−11^
AB462810	2	CFA	Japan	nd	Asia-3	7.74 × 10^−28^	1.01 × 10^−26^	3.87 × 10^−21^	5.43 × 10^−7^	5.32 × 10^−7^	3.00 × 10^−8^	1.32 × 10^−11^
AB474397	1 ^	CFA	Japan	nd	Asia-4	9.95 × 10^−28^	4.86 × 10^−26^	4.93 × 10^−23^	4.67 × 10^−8^	8.28 × 10^−8^	0.000173	1.32 × 10^−11^
AB474397	2	CFA	Japan	nd	Asia-5	7.74 × 10^−28^	1.01 × 10^−26^	3.87 × 10^−21^	5.43 × 10^−7^	5.32 × 10^−7^	3.00 × 10^−8^	1.32 × 10^−11^
AY443350	1 ^	PLO	USA	2000	Amer-2	3.23 × 10^−12^	5.27 × 10^−18^	4.42 × 10^−16^	1.00 × 10^−11^	7.02 × 10^−13^	3.73 × 10^−16^	2.21 × 10^−16^
AY443350	2 ^	PLO	USA	2000	Amer-2	2.34 × 10^−12^	0.007073	2.08 × 10^−11^	3.85 × 10^−15^	0.000116	4.74 × 10^−7^	6.62 × 10^−12^
AY445077	1 ^	PLO	USA	1998	Amer-1 *	3.16 × 10^−19^	6.42 × 10^−13^	1.90 × 10^−16^	1.32 × 10^−9^	3.47 × 10^−9^	1.09 × 10^−10^	6.62 × 10^−12^
AY445077	2 ~	PLO	USA	1998	Amer-1 *	6.21 × 10^−8^	8.26 × 10^−8^	4.02 × 10^−5^	0.020799	0.005608	NS	1.04 × 10^−5^
AY466011	1	PLO	USA	1998	Amer-1 *	3.16 × 10^−19^	6.42 × 10^−13^	1.90 × 10^−16^	1.32 × 10^−9^	3.47 × 10^−9^	1.09 × 10^−10^	6.62 × 10^−12^
AY466011	2 ~	PLO	USA	1998	Amer-1 *	6.21 × 10^−8^	8.26 × 10^−8^	4.02 × 10^−5^	0.020799	0.005608	NS	1.04 × 10^−5^
AY542312	1 ^	PLO	USA	1998	Amer-1 *	3.16 × 10^−19^	6.42 × 10^−13^	1.90 × 10^−16^	1.32 × 10^−9^	3.47 × 10^−9^	1.09 × 10^−10^	6.62 × 10^−12^
AY542312	2 ~	PLO	USA	1998	Amer-1 *	6.21 × 10^−8^	8.26 × 10^−8^	4.02 × 10^−5^	0.020799	0.005608	NS	1.04 × 10^−5^
AY649446	1 ^	PLO	USA	2001	Amer-2	4.64 × 10^−11^	1.04 × 10^−14^	1.03 × 10^−8^	0.000218	0.000172	0.01701	9.42 × 10^−9^
AY649446	2 ^~	PLO	USA	2001	Amer-2	NS	0.003004	0.004893	9.18 × 10^−5^	0.000131	8.85 × 10^−16^	NS
HQ540293	1	FOX	China	2006	Asia-1	3.89 × 10^−71^	4.39 × 10^−69^	4.91 × 10^−63^	4.81 × 10^−18^	1.78 × 10^−17^	2.67 × 10^−17^	1.99 × 10^−11^
JX681125	1	FOX	China	2006	Asia-1	3.89 × 10^−71^	4.39 × 10^−69^	4.91 × 10^−63^	4.81 × 10^−18^	1.78 × 10^−17^	2.67 × 10^−17^	1.99 × 10^−11^
KJ123771	1 ~	CFA	USA	2004	Amer-2	NS	0.000126	2.35 × 10^−9^	2.23 × 10^−7^	2.14 × 10^−8^	1.41 × 10^−15^	2.58 × 10^−10^
KJ994343	1	NPR	Canada	2013	Asia-1	7.52 × 10^−33^	1.95 × 10^−31^	1.98 × 10^−26^	1.42 × 10^−5^	1.12 × 10^−5^	3.55 × 10^−6^	1.99 × 10^−11^
KJ994343	2 ^~	NPR	Canada	2013	Asia-1	1.06 × 10^−9^	1.69 × 10^−10^	2.39 × 10^−6^	5.91 × 10^−8^	1.80 × 10^−8^	2.53 × 10^−5^	9.47 × 10^−8^
KJ994343	3 ~	NPR	Canada	2013	Asia-1	1.31 × 10^−7^	0.000298	1.72 × 10^−5^	0.012219	0.040604	NS	6.46 × 10^−5^
KJ994343	4	NPR	Canada	2013	Asia-1	0.003985	NS	0.012217	2.17 × 10^−6^	2.50 × 10^−6^	NS	7.89 × 10^−8^
KJ994343	5 ~	NPR	Canada	2013	Asia-1	2.93 × 10^−5^	5.18 × 10^−5^	6.73 × 10^−7^	0.003271	0.001688	0.000122	0.039698

^ The recombinant sequence may have been misidentified (one of the identified parents might be the recombinant). ~ It is possible that this apparent recombination signal could have been caused by an evolutionary process other than recombination. * Lineage is conventionally determined by hemagglutinin (H) gene diversity. In this case, the H gene sequence was the recombinant portion of the genome and thus defined the genotype; however, the majority of genome is America-2. Abbreviations: Acc. no. = Accession number, # = recombination event per sequence, GC = GENECONV, BS = BootScan, MC = MaxChi, Ch = Chimaera, SS = SiScan, CFA = domestic dog, PLO = raccoon, FOX = red fox, NPR = raccoon dog.

**Table 3:** Candidate markers of CDV pathogenicity in lions. Shading indicates sites at which pervasive positive and/or episodic diversifying selection was detected in selection analysis of all protein coding regions of available unique CDV whole-genome sequences (*n* = 108), while all unshaded sites were found to experience negative, i.e., purifying selection.

WGS Position (nt)	Gene	AA	Sub-Clinical AA	Clinical AA	Functional Domains	Putative Function	Virus Process
1361	N	418 *	-	-	N-tail, box 1	Binds unidentified host receptor suppressing immune cell proliferation	Transcription & replication
1458	N	451	F	L	N-tail	Binds P recruiting RdRp complex to prevent slipping during transcription & replication	Transcription & replication
1505	N	466	-	-	N-tail	Binds P recruiting RdRp complex to prevent slipping during transcription & replication	Transcription & replication
2638 ^¥^	P/V	280 *	K	E/R	PNT/V-Zbd	P - binds L - essential co-factor of transcription and replication/V - interrupts host innate immune response, virulence factor	Transcription & replication
2832	P	344	-	-	PMD	L binding site - essential co-factor of transcription and replication	Transcription & replication
2934	P	378	-	-	PMD	L binding site - essential co-factor of transcription and replication	Transcription & replication
3457	M	9	Q	R	M - NTD	Matrix general - modulates fusion and budding, binds F, H, RNP, and cellular actin, assembles components at surface	Assembly & fusion
3461	M	10	-	-	M - NTD	Matrix general - modulates fusion and budding, binds F, H, RNP, and cellular actin, assembles components at surface	Assembly & fusion
3821	M	130	-	-	M - NTD	Matrix general - modulates fusion and budding, binds F, H, RNP, and cellular actin, assembles components at surface	Assembly & fusion
4436	M	335	-	-	M - CTD	Matrix general - modulates fusion and budding, binds F, H, RNP, and cellular actin, assembles components at surface	Assembly & fusion
5978	F	348	-	-	F1	Extracellular domain of glycoprotein, mediates fusion with host cell, interacts with H	Fusion
6905	F	657	-	-	F tail	Cytoplasmic tail interacts with M at cell surface during fusion and budding	Assembly & fusion
7870	H	264	-	-	no data	No data	Host receptor binding
8634	H	519	R	I	SLAM binding	Host immune cell recognition and entry	Host receptor binding
8723	H	549 *	Y	H	SLAM binding	Host immune cell recognition and entry	Host receptor binding
9290 *	L	87	-	-	LRI	No data	Transcription & replication
9308	L	93 †,∞	L	F	LRI	No data	Transcription & replication
9428	L	133	-	-	LRI	No data	Transcription & replication
10844	L	605	-	-	CRII	Polymerase activity	Transcription & replication
11159	L	710	-	-	CRIII	Catalytic center for polymerization, i.e., transcription and replication of vRNA	Transcription & replication
12170	L	1047 *	-	-	CRIV	mRNA capping, essential for translation and immune escape	Transcription & replication
12986	L	1319 *	-	-	CRV	Methylation of viral mRNA	Transcription & replication
13233	L	1402 †	N	H	Connector	spacing the catalytic domains, may interact with P to stabilize conformation of RdRp complex	Transcription & replication
13886	L	1619 ‡	-	-	Connector	Spacing the catalytic domains, may interact with P to stabilize conformation of RdRp complex	Transcription & replication
14132	L	1701	-	-	Linker	Separates regions of the L	Transcription & replication
15056	L	2009	-	-	Mtase	Methylation of viral mRNA	Transcription & replication
15203	L	2058 †	A	C	Mtase	Methylation of viral mRNA	Transcription & replication
15326	L	2099	-	-	Mtase	Methylation of viral mRNA	Transcription & replication

* Differentiates canids and non-canids in 1993–1994 Serengeti CDV outbreak. ^¥^ Occurs in overlapping open reading frame. † Differentiates canids and non-canids in 1994 with exception of PLE-658. ∞ Both pervasive purifying selection and episodic diversifying selection detected at this site. ‡ The clinical strain in East Africa agrees with the consensus residue at this site, and subclinical strains have the alternative allele. Thus, this is not considered a candidate marker of pathogenicity in lions.

## Supplementary Materials

The following are available online at https://www.mdpi.com/2076-0817/9/11/872/s1: Table S1. List of canine distemper virus near whole-genome sequences generated in this study (*n* = 21) with associated data. Table S2. Accession numbers of near whole-genome sequences used in this study (*n* = 101) with associated data. Table S3. Loci on canine distemper virus genome identified as experiencing pervasive positive or episodic diversifying selection (*n* = 133) in analysis of all available whole-genome sequence data from multiple host species. Table S4. Loci on canine distemper virus genome identified as experiencing negative or purifying selection by SLAC and FEL selection analyses of all available whole-genome sequence data implemented with the online data server Datamonkey (*n* = 1311).
