# Supplementary material for: Identifying Candidate Genetic Markers of CDV Cross-Species Pathogenicity in African Lions
Source: Pathogens. 2020 Oct 23;9(11):872. doi: 10.3390/pathogens9110872 (PMC7690837; doi:10.3390/pathogens9110872)
Supplement: Supplementary file 1 [file pathogens-09-00872-s001.zip › pathogens-934746-supplementary.docx]

Supplementary Materials to accompany

Identifying candidate genetic markers of CDV cross-species pathogenicity in African lions

Julie K. Weckworth ^1,7,^*, Brian W. Davis ^2^, Melody E. Roelke-Parker ^3^, Rebecca P. Wilkes ^4^, Craig Packer ^5^, Ernest Eblate ^6^, Michael K. Schwartz ^1,7^ and L. Scott Mills ^8,‡^

**Supplementary Materials**

**Table S1.** List of canine distemper virus near whole genome sequences generated in this study (*n* = 21) with associated data. Country abbreviations: USA = United States, KYA = Kenya, TZA = Tanzania.

| **Label** | **Origin** | **Date Sampled** | **Common Name** | **Accession No.** |
| --- | --- | --- | --- | --- |
| PLE806_USA_1992 | USA | 09/01/1992 | African lion | MT932504 |
| PLO004_USA_1992 | USA | 01/01/1992 | Raccoon | MT932505 |
| PLO005_USA_2013 | USA | 03/02/2013 | Raccoon | MT932506 |
| PLO006_USA_2013 | USA | 03/13/2013 | Raccoon | MT932508 |
| PLO007_USA_2013 | USA | 03/26/2013 | Raccoon | MT932509 |
| PLO008_USA_2013 | USA | 04/09/2013 | Raccoon | MT932510 |
| PPA202_USA_1992 | USA | 10/15/1992 | Common leopard | MT932511 |
| UCI001_USA_2013 | USA | 03/29/2013 | Gray fox | MT932507 |
| CFA203_USA_2013 | USA | 01/22/2013 | Domestic dog | MT932494 |
| CFA204_USA_2013 | USA | 06/24/2013 | Domestic dog | MT932495 |
| CFA205_USA_2013 | USA | 06/07/2013 | Domestic dog | MT932496 |
| CFA207_USA_2013 | USA | 11/12/2013 | Domestic dog | MT932497 |
| CFA209_USA_2013 | USA | 11/12/2013 | Domestic dog | MT932491 |
| CFA210_USA_2013 | USA | 11/12/2012 | Domestic dog | MT932498 |
| CFADD19_KYA_2000 | KYA | 02/28/2000 | Domestic dog | MT932499 |
| CFADD24_KYA_2000 | KYA | 02/28/2000 | Domestic dog | MT932500 |
| CME10_KYA_2000 | KYA | 03/06/2000 | Black-backed jackal | MT932493 |
| CME11_KYA_2000 | KYA | 03/03/2000 | Black-backed jackal | MT932501 |
| CME16_KYA_2000 | KYA | 03/17/2000 | Black-backed jackal | MT932502 |
| JSP1_TZA_2006 | TZA | 07/20/2006 | Jackal species | MT932492 |
| LPI1561_TZA_2007 | TZA | 10/03/2007 | African wild dog | MT932503 |

**Table S2.** Accession numbers of whole canine distemper virus genome sequences used in this study (*n* = 98) with associated data.

| **Accession No** | **Label** | **Species** | **Origin** | **Date** | **Year Sampled** |
| --- | --- | --- | --- | --- | --- |
| AB462810 | AB462810_CFA_JAP_nd | CFA | JAP | No data | No data |
| AB474397 | AB474397_CFA_JAP_nd | CFA | JAP | No data | No data |
| AB475097 | AB475097_CFA_JAP_nd | CFA | JAP | No data | No data |
| AB475099 | AB475099_CFA_JAP_nd | CFA | JAP | No data | No data |
| AB476401 | AB476401_CFA_JAP_nd | CFA | JAP | No data | No data |
| AB476402 | AB476402_CFA_JAP_nd | CFA | JAP | No data | No data |
| AB490670 | AB490670_CFA_JAP_nd | CFA | JAP | No data | No data |
| AB490672 | AB490672_CFA_JAP_nd | CFA | JAP | No data | No data |
| AB490674 | AB490674_CFA_JAP_nd | CFA | JAP | No data | No data |
| AB490676 | AB490676_CFA_JAP_nd | CFA | JAP | No data | No data |
| AB490678 | AB490678_CFA_JAP_nd | CFA | JAP | No data | No data |
| AB490679 | AB490679_CFA_JAP_nd | CFA | JAP | No data | No data |
| AB490680 | AB490680_CFA_JAP_nd | CFA | JAP | No data | No data |
| AB490681 | AB490681_CFA_JAP_nd | CFA | JAP | No data | No data |
| AB687720 | AB687720 _MKY_JAP_2008 | MKY | JAP | 7/1/08 | 2008 |
| AB687721 | AB687721_MKY_JAP_2008 | MKY | JAP | 7/1/08 | 2008 |
| AB753775 | AB753775_CFA_JAP_nd | CFA | No data | No data | No data |
| AB753776 | AB753776_CFA_nd_nd | CFA | No data | No data | No data |
| AB823706 | AB823706_CFA_nd_nd | CFA | No data | No data | No data |
| AB823707 | AB823707_CFA_nd_nd | CFA | No data | No data | No data |
| AF014953 | AF014953_VAC_nd_nd | VAC | No data | No data | No data |
| AF164967 | AF164967_CFA_nd_1975 | CFA | No data | No data | No data |
| AF305419 | AF305419_VAC_nd_1950_Onderstepoort | VAC | No data | No data | No data |
| AF378705 | AF378705_VAC_nd_nd_Onderstepoort(sm) | VAC | No data | No data | No data |
| AY386315 | AY386315_CFA_nd_nd | CFA | No data | No data | No data |
| AY386316 | AY386316_MPU_nd_nd | MPU | No data | No data | No data |
| AY443350 | AY443350_PLO_USA_2000 | PLO | USA | 1/1/00 | 2000 |
| AY445077 | AY445077_PLO_USA_1998 | PLO | USA | 1/1/98 | 1998 |
| AY466011 | AY466011_PLO_USA_1998 | PLO | USA | 1/1/98 | 1998 |
| AY542312 | AY542312_PLO_USA_1998 | PLO | USA | 1/1/98 | 1998 |
| AY649446 | AY649446_PLO_USA_2001 | PLO | USA | 1/1/01 | 2001 |
| EU716337 | EU716337_CFA_USA_2004 | CFA | USA | 9/4/04 | 2004 |
| EU726268 | EU726268_VAC_CHN_nd_CDV3 | MNK | CHN | No data | No data |
| GU138403 | GU138403_MPU_CH_1956_SnyderHill | MPU | No data | No data | No data |
| HM046486 | HM046486_PSI_KZN_2007 | PSI | KZN | 1/1/07 | 2007 |
| HM063009 | HM063009_MINK_KZN_1989 | MINK | KZN | 1/1/89 | 1989 |
| HM852904 | HM852904_MKY_CHN_2008 | MKY | CHN | 1/1/08 | 2008 |
| HQ540292 | HQ540292_CFA_CHN_2007 | CFA | CHN | No data | 2007 |
| HQ540293 | HQ540293_FOX_CHN_2006 | FOX | CHN | No data | 2006 |
| JN896331 | JN896331_CFA_CHN_2010 | CFA | CHN | 1/1/10 | 2010 |
| JN896987 | JN896987_Snyder_nd_nd | VAC | No data | No data | No data |
| JX681125 | JX681125_FOX_CHN_2006 | FOX | CHN | 7/1/06 | 2006 |
| KC427278 | KC427278_MINK_CHN_2008 | MNK | CHN | 7/6/08 | 2008 |
| KF640687 | KF640687_CFA_USA_nd | CFA | USA | No data | No data |
| KF856711 | KF856711_MKY_CHN_2006 | MKY | CHN | 1/1/06 | 2006 |
| KF914669 | KF914669_CFA_ITY_2013 | CFA | ITY | 5/15/13 | 2013 |
| KJ123771 | KJ123771_CFA_USA_2004 | CFA | USA | 1/1/04 | 2004 |
| KJ466106 | KJ466106_NPR_CHN_2012 | NPR | CHN | 1/1/12 | 2012 |
| KJ747371 | KJ747371_FOX_USA_2013 | FOX | USA | 1/1/13 | 2013 |
| KJ747372 | KJ747372_CFA_USA_2013 | CFA | USA | 1/1/13 | 2013 |
| KJ848781 | KJ848781_NPR_CAN_2014 | NPR | CAN | 4/1/14 | 2014 |
| KJ994343 | KJ994343_NPR_CAN_2013 | NPR | CAN | 10/1/13 | 2013 |
| KM280689 | KM280689_CFA_UGY_2012 | CFA | UGY | 1/1/12 | 2012 |
| KM926612 | KM926612_MPU_CHN_1992 | MPU | CHN | 1/1/92 | 1992 |
| KP677502 | KP677502_AME_CHN_2015 | AME | CHN | 1/4/15 | 2015 |
| KP738610 | KP738610_NPR_CHN_2014 | NPR | CAN | 10/1/14 | 2014 |
| KP765763 | KP765763_FOX_CHN_2014 | FOX | CAN | 7/3/14 | 2014 |
| KP765764 | KP765764_FOX_CHN_2005 | FOX | CHN | 7/2/05 | 2005 |
| KP793921 | KP793921_AME_CHN_2014 | AME | CHN | 7/6/05 | 2014 |
| KU578253 | KU578253_AWD_TZA_2007 | AWD | TZA | 1/1/07 | 2007 |
| KU578254 | KU578254_CAU_TZA_2011 | CAU | TZA | 1/1/11 | 2011 |
| KU666057 | KU666057_PLO_USA_2012 | PLO | USA | 11/24/12 | 2012 |
| KX024708 | KX024708_MME_ITY_2015 | MME | ITY | 9/28/15 | 2015 |
| KX024709 | KX024709_MME_ITY_2015 | MME | ITY | 10/5/15 | 2015 |
| KX347928 | KX347928_CFA_CHN_2015 | CFA | CHN | 10/10/15 | 2015 |
| KX499865 | KX499865_FOX_CHN_2015 | FOX | CHN | 7/7/05 | 2015 |
| KX709880 | KX709880_CFA_CHN_2012 | CFA | CHN | 7/18/12 | 2012 |
| KX774415 | KX774415_PTI_RSA_2004 | PTI | RSA | 1/1/04 | 2004 |
| KY971528 | KY971528_AWD_SAF_2016 | AWD | SAF | 5/1/16 | 2016 |
| KY971529 | KY971529_VAC_nd_nd_Bucharest | VAC | No data | No data | No data |
| KY971530 | KY971530_VAC_nd_nd_'Novi' | VAC | No data | No data | No data |
| KY971531 | KY971531_VAC_nd_nd_Ovi | VAC | No data | No data | No data |
| KY971532 | KY971532_CCR_SAF_2017 | CCR | SAF | 1/1/17 | 2017 |
| LC159587 | LC159587_CFA_VNM_2014 | CFA | VNM | 8/16/14 | 2014 |
| MF041963 | MF041963_CSI_EPA_2016 | CSI | EPA | 9/30/16 | 2016 |
| NC_001921 | NC_001921_VAC_nd_nd | VAC | No data | No data | No data |
| LC338064 | LC338064_CFA_JAP_1997 | CFA | JAP | No data | 1997 |
| MT136707.1 | CCR10_TZA_1994 | CCR | TZA | 06/20/1994 | 1994 |
| MT136708.1 | CCR11BH_TZA_1994 | CCR | TZA | 07/03/1994 | 1994 |
| MT136709.1 | CCR12_TZA_1994 | CCR | TZA | 07/09/1994 | 1994 |
| MT136710.1 | CCR282_TZA_1994 | CCR | TZA | 01/18/1994 | 1994 |
| MT136705.1 | CCR6_TZA_1993 | CCR | TZA | 12/20/1993 | 1994 |
| MT136706.1 | CCR7BR_TZA_1994 | CCR | TZA | 12/23/1993 | 1994 |
| MT136711.1 | CFA51_TZA_1994 | CFA | TZA | 09/09/1994 | 1994 |
| MT136712.1 | CFA52_TZA_1994 | CFA | TZA | 08/23/1994 | 1994 |
| MT136713.1 | CFA54_TZA_1994 | CFA | TZA | 11/18/1994 | 1994 |
| MT136714.1 | OME8SP_TZA_1994 | OME | TZA | 07/16/1994 | 1994 |
| MT136715.1 | OME9_TZA_1994 | OME | TZA | 07/25/1994 | 1994 |
| MT136716.1 | PLE589_TZA_1994 | PLE | TZA | 01/22/1994 | 1994 |
| MT136717.1 | PLE595_TZA_1994 | PLE | TZA | 02/03/1994 | 1994 |
| MT136718.1 | PLE635LN_TZA_1994 | PLE | TZA | 02/18/1994 | 1994 |
| MT136719.1 | PLE640LN_TZA_1994 | PLE | TZA | 02/07/1994 | 1994 |
| MT136720.1 | PLE641_TZA_1994 | PLE | TZA | 11/15/1994 | 1994 |
| MT136721.1 | PLE652_TZA_1994 | PLE | TZA | 07/30/1994 | 1994 |
| MT136722.1 | PLE653_TZA_1994 | PLE | TZA | 01/28/1994 | 1994 |
| MT136723.1 | PLE654_TZA_1994 | PLE | TZA | 01/31/1994 | 1994 |
| MT136724.1 | PLE656_TZA_1994 | PLE | TZA | 02/20/1994 | 1994 |
| MT136725.1 | PLE658_TZA_1994 | PLE | TZA | 05/21/1994 | 1994 |

Host abbreviations: AME = giant panda, AWD = African wild dog, CAU = golden jackal, CCR = spotted hyena, CFA = domestic dog, CSI = Ethiopian wolf, FOX = fox species, MKY = monkey species, MME = European badger, MNK = mink, MPU = ferret, NPR = raccoon dog, OME = bat eared fox, PLE = African lion, PLO = raccoon, PSI = seal, PTI = tiger, VAC = vaccine. Country abbreviations: CAN = Canada, CHN = China, EPA = Ethiopia, JAP = Japan, ITY = Italy, KZN = Kazakhstan, RSA = Russia, SAF = South Africa, TZA = Tanzania, USA = United States, VNM = Vietnam.

**Table S3.** Codons on canine distemper virus genome identified as experiencing pervasive positive and/or episodic diversifying selection (*n* = 118) in analysis of coding region sequence data from multiple host species. Shading indicate sites that differentiate clinical and subclinical strains in circulation in East Africa from 1992-2011.

| **Codon*** | **SLAC dN-dS** | **SLAC *p*-Value** | **FEL dN/dS** | **FEL *p*-Value** | **MEME β⁺** | **MEME *p*-Value** |
| --- | --- | --- | --- | --- | --- | --- |
| 134 | 4.770 | 0.133 | Infinity | 0.052 | 19.18 | 0.01 |
| 353 | 0.478 | 0.667 | 1.487 | 0.904 | 425.9 | 0 |
| 354 | -2.082 | 0.892 | 0.346 | 0.312 | 5553.55 | 0 |
| 383 | 0.944 | 0.699 | Infinity | 0.378 | 222.78 | 0.1 |
| 451 | 3.535 | 0.271 | Infinity | 0.087 | 14.66 | 0.08 |
| 456 | 6.233 | 0.096 | Infinity | 0.036 | 4.64 | 0.05 |
| 466 | -4.780 | 0.988 | 0.175 | 0.101 | 521.89 | 0.09 |
| 467 | 2.693 | 0.397 | Infinity | 0.196 | 67.45 | 0.04 |
| 588 | 1.892 | 0.488 | Infinity | 0.209 | 54.57 | 0.08 |
| 596 | 1.913 | 0.444 | Infinity | 0.177 | 32.05 | 0.09 |
| 613 | 4.776 | 0.132 | Infinity | 0.043 | 11.49 | 0.02 |
| 617 | 0.944 | 0.699 | Infinity | 0.377 | 10000 | 0.01 |
| 629 | 3.975 | 0.195 | Infinity | 0.1 | 1.42 | 0.13 |
| 646 | 0.945 | 0.675 | Infinity | 0.35 | 242.45 | 0.09 |
| 655 | 1.912 | 0.445 | Infinity | 0.241 | 189.06 | 0.03 |
| 671 | 3.809 | 0.201 | Infinity | 0.076 | 1.7 | 0.1 |
| 672 | 0.878 | 0.726 | Infinity | 0.472 | 578.85 | 0.06 |
| 677 | 0.878 | 0.726 | Infinity | 0.472 | 373.9 | 0.08 |
| 718 | 5.728 | 0.095 | Infinity | 0.029 | 2.17 | 0.04 |
| 744 | 3.817 | 0.206 | Infinity | 0.073 | 1.38 | 0.1 |
| 747 | 0.318 | 0.699 | 11.757 | 0.673 | 274.48 | 0 |
| 753 | 0.937 | 0.701 | Infinity | 0.375 | 461.38 | 0.07 |
| 758 | 3.850 | 0.194 | Infinity | 0.08 | 1.38 | 0.1 |
| 779 | 3.825 | 0.198 | Infinity | 0.062 | 1.51 | 0.08 |
| 792 | 3.825 | 0.198 | Infinity | 0.043 | 1.72 | 0.06 |
| 796 | 0.956 | 0.667 | Infinity | 0.371 | 171.36 | 0.07 |
| 803 | 2.821 | 0.343 | Infinity | 0.12 | 74.05 | 0.04 |
| 819 | 5.766 | 0.087 | Infinity | 0.03 | 2.06 | 0.04 |
| 839 | 1.362 | 0.647 | Infinity | 0.373 | 285.28 | 0.1 |
| 851 | -1.453 | 0.925 | 0.27 | 0.375 | 4660.48 | 0.03 |
| 903 | -1.426 | 0.924 | 0.376 | 0.5 | 10000 | 0.02 |
| 1014 | 0.878 | 0.726 | Infinity | 0.472 | 3030.01 | 0.02 |
| 1034 | -2.876 | 0.963 | 0.341 | 0.418 | 4039.3 | 0.01 |
| 1039 | 4.331 | 0.217 | Infinity | 0.066 | 1.82 | 0.09 |
| 1105 | -2.785 | 0.957 | 0.328 | 0.351 | 1304.18 | 0.05 |
| 1114 | -5.743 | 0.982 | 0.177 | 0.043 | 408.2 | 0.02 |
| 1184 | -5.292 | 0.990 | 0.105 | 0.034 | 924.97 | 0.08 |
| 1208 | -1.453 | 0.925 | 0.319 | 0.438 | 513.9 | 0.1 |
| 1231 | 1.830 | 0.516 | Infinity | 0.305 | 82.52 | 0.01 |
| 1255 | 0.342 | 0.692 | 0.999 | 1 | 62.21 | 0.09 |
| 1257 | 1.877 | 0.491 | Infinity | 0.208 | 129.76 | 0.05 |
| 1342 | 2.090 | 0.418 | Infinity | 0.192 | 239.87 | 0.02 |
| 1368 | 6.309 | 0.095 | Infinity | 0.023 | 23.53 | 0.01 |
| 1369 | 5.386 | 0.154 | Infinity | 0.057 | 2.54 | 0.08 |
| 1371 | 4.779 | 0.132 | Infinity | 0.039 | 5.29 | 0.04 |
| 1372 | 6.171 | 0.127 | Infinity | 0.049 | 2.82 | 0.07 |
| 1376 | 3.823 | 0.198 | Infinity | 0.066 | 3.35 | 0.09 |
| 1378 | 2.265 | 0.420 | Infinity | 0.264 | 7.83 | 0.09 |
| 1386 | 9.943 | 0.033 | Infinity | 0.01 | 4.73 | 0.02 |
| 1391 | 4.524 | 0.203 | Infinity | 0.083 | 2.03 | 0.11 |
| 1393 | 6.640 | 0.062 | Infinity | 0.016 | 2.62 | 0.03 |
| 1404 | 6.705 | 0.246 | Infinity | 0.084 | 3.03 | 0.11 |
| 1407 | 3.824 | 0.198 | Infinity | 0.1 | 3.41 | 0.12 |
| 1408 | 4.365 | 0.216 | Infinity | 0.066 | 4.08 | 0.09 |
| 1416 | 7.245 | 0.044 | Infinity | 0.019 | 16.26 | 0.02 |
| 1418 | 6.235 | 0.102 | Infinity | 0.009 | 9.73 | 0.01 |
| 1419 | -1.976 | 0.867 | 0.552 | 0.52 | 1244.1 | 0.04 |
| 1423 | 3.624 | 0.259 | Infinity | 0.077 | 1.71 | 0.1 |
| 1429 | 4.776 | 0.132 | Infinity | 0.037 | 1.92 | 0.05 |
| 1437 | 5.546 | 0.132 | Infinity | 0.028 | 2.75 | 0.04 |
| 1452 | 2.841 | 0.305 | Infinity | 0.066 | 1.56 | 0.09 |
| 1470 | 0.301 | 0.693 | 1.228 | 0.872 | 102.14 | 0 |
| 1471 | 5.027 | 0.249 | Infinity | 0.097 | 18.53 | 0.03 |
| 1481 | 4.733 | 0.179 | Infinity | 0.06 | 14.42 | 0.06 |
| 1502 | 0.692 | 0.922 | Infinity | 0.665 | 486.51 | 0.07 |
| 1529 | 2.943 | 0.358 | Infinity | 0.137 | 544.97 | 0.01 |
| 1560 | 1.913 | 0.445 | Infinity | 0.178 | 95.64 | 0.04 |
| 1676 | 0.956 | 0.667 | Infinity | 0.371 | 1357.91 | 0.02 |
| 1719 | -1.185 | 0.908 | 0.352 | 0.483 | 3540.79 | 0.02 |
| 1731 | 2.651 | 0.375 | Infinity | 0.2 | 7.07 | 0.07 |
| 1911 | 0.957 | 0.593 | 1.472 | 0.729 | 866.06 | 0.01 |
| 1942 | 0.882 | 0.724 | Infinity | 0.412 | 1505.53 | 0.04 |
| 1972 | -0.960 | 0.889 | 0.472 | 0.6 | 2415.55 | 0.03 |
| 1974 | -1.479 | 0.855 | 0.405 | 0.517 | 905.11 | 0.01 |
| 1976 | -10.670 | 1.000 | 0.055 | 0.002 | 1205.01 | 0.05 |
| 2001 | 1.908 | 0.447 | Infinity | 0.242 | 11.41 | 0.04 |
| 2002 | 3.750 | 0.215 | Infinity | 0.058 | 1.73 | 0.08 |
| 2004 | 3.768 | 0.257 | Infinity | 0.084 | 1.64 | 0.11 |
| 2008 | 0.957 | 0.667 | Infinity | 0.346 | 272.71 | 0.08 |
| 2019 | 0.958 | 0.667 | Infinity | 0.41 | 2880.71 | 0.02 |
| 2051 | 0.958 | 0.667 | Infinity | 0.341 | 226.82 | 0.09 |
| 2071 | 1.893 | 0.455 | Infinity | 0.186 | 569.86 | 0.01 |
| 2077 | 0.936 | 0.568 | 1.948 | 0.581 | 263.84 | 0.09 |
| 2205 | -1.917 | 0.889 | 0.466 | 0.454 | 126.52 | 0.1 |
| 2269 | -0.310 | 0.783 | 0.954 | 0.971 | 121.5 | 0.1 |
| 2303 | -0.769 | 0.757 | 0.82 | 0.824 | 10000 | 0 |
| 2339 | 1.364 | 0.647 | Infinity | 0.374 | 1371.18 | 0.05 |
| 2391 | 0.958 | 0.667 | Infinity | 0.407 | 162.38 | 0.08 |
| 2404 | 1.835 | 0.516 | Infinity | 0.312 | 612.11 | 0.01 |
| 2426 | -2.822 | 0.959 | 0.193 | 0.167 | 507.27 | 0.09 |
| 2442 | 3.836 | 0.197 | Infinity | 0.094 | 13.69 | 0.09 |
| 2487 | 3.828 | 0.198 | Infinity | 0.072 | 107.58 | 0 |
| 2498 | 0.743 | 0.647 | 1.467 | 0.754 | 3615.8 | 0 |
| 2537 | 2.997 | 0.205 | Infinity | 0.086 | 3.17 | 0.11 |
| 2546 | 0.721 | 0.637 | 0.987 | 0.988 | 163.54 | 0.09 |
| 2576 | 10.338 | 0.023 | Infinity | 0.002 | 5.56 | 0 |
| 2587 | 0.880 | 0.726 | Infinity | 0.473 | 1366.9 | 0.03 |
| 2647 | 0.946 | 0.699 | Infinity | 0.377 | 1154 | 0.04 |
| 2727 | -6.198 | 0.994 | 0.116 | 0.028 | 1042.47 | 0.06 |
| 2767 | 0.000 | 0.741 | 0.945 | 0.963 | 272.67 | 0 |
| 2780 | -0.959 | 0.889 | 0.486 | 0.617 | 339.88 | 0.1 |
| 2850 | -4.782 | 0.988 | 0.159 | 0.082 | 947.31 | 0.06 |
| 2928 | 0.892 | 0.737 | Infinity | 0.454 | 546.63 | 0.06 |
| 3014 | 2.862 | 0.299 | Infinity | 0.065 | 1.51 | 0.09 |
| 3245 | 4.747 | 0.149 | Infinity | 0.079 | 1.79 | 0.1 |
| 3263 | -0.957 | 0.889 | 0.39 | 0.521 | 395.83 | 0.1 |
| 3285 | 0.945 | 0.699 | Infinity | 0.376 | 250.09 | 0.09 |
| 3433 | -4.787 | 0.988 | 0.174 | 0.101 | 789.31 | 0.07 |
| 3520 | -5.973 | 0.993 | 0.151 | 0.076 | 1011.36 | 0.07 |
| 3702 | 0.946 | 0.675 | Infinity | 0.351 | 709.53 | 0.04 |
| 3991 | -7.658 | 0.993 | 0.172 | 0.028 | 10000 | 0.01 |
| 4023 | 0.957 | 0.667 | Infinity | 0.406 | 334.97 | 0.07 |
| 4036 | 0.882 | 0.724 | Infinity | 0.413 | 1674.83 | 0.03 |
| 4050 | 0.879 | 0.726 | Infinity | 0.501 | 195.12 | 0.1 |
| 4373 | 1.710 | 0.440 | Infinity | 0.22 | 34.25 | 0.02 |
| 4378 | 0.497 | 0.617 | 1.019 | 0.989 | 36.87 | 0.1 |
| 4511 | 0.635 | 0.595 | 1.35 | 0.841 | 34.13 | 0.06 |
| 4691 | -0.702 | 0.828 | 0.63 | 0.716 | 33.34 | 0.07 |

*Codon = position on the coding region of canine distemper virus genome without stop codons. * cutoff p ≤ 0.10. ^ codon = amino acid position in the coding region of the CDV genome without stop codons.

**Table S4.** Loci on coding region of canine distemper virus genome identified as experiencing negative or purifying selection by SLAC and FEL analyses (HYPHY) of all available coding region sequence data (*n* = 1405). Shading indicates sites that differentiate *clinical* and *subclinical* strains in circulation in East Africa between 1992–2011.

| **Codon*** | | **SLAC dN-dS** | | **SLAC *p*-Value** | | **FEL dN/dS** | | **FEL *p*-Value** |  |
| --- | --- | --- | --- | --- | --- | --- | --- | --- | --- |
| 2 | | -3.825 | | 0.111 | | 0 | | 0.024 |  |
| 3 | | -2.306 | | 0.276 | | 0 | | 0.094 |  |
| 5 | | -4.474 | | 0.127 | | 0.159 | | 0.084 |  |
| 6 | | -4.451 | | 0.087 | | 0 | | 0.031 |  |
| 7 | | -2.307 | | 0.276 | | 0 | | 0.093 |  |
| 10 | | -6.732 | | 0.04 | | 0 | | 0.007 |  |
| 14 | | -3.825 | | 0.111 | | 0 | | 0.024 |  |
| 21 | | -5.738 | | 0.037 | | 0 | | 0.008 |  |
| 22 | | -3.825 | | 0.122 | | 0 | | 0.049 |  |
| 25 | | -3.825 | | 0.118 | | 0 | | 0.025 |  |
| 28 | | -7.905 | | 0.081 | | 0 | | 0.029 |  |
| 30 | | -3.825 | | 0.112 | | 0 | | 0.033 |  |
| 33 | | -2.33 | | 0.274 | | 0 | | 0.09 |  |
| 39 | | -7.874 | | 0.011 | | 0 | | 0.003 |  |
| 40 | | -3.825 | | 0.111 | | 0 | | 0.033 |  |
| 42 | | -2.328 | | 0.274 | | 0 | | 0.087 |  |
| 43 | | -3.825 | | 0.125 | | 0 | | 0.039 |  |
| 48 | | -2.029 | | 0.321 | | 0 | | 0.084 |  |
| 51 | | -3.138 | | 0.227 | | 0 | | 0.074 |  |
| 53 | | -4.658 | | 0.075 | | 0 | | 0.014 |  |
| 57 | | -2.075 | | 0.316 | | 0 | | 0.097 |  |
| 59 | | -3.825 | | 0.111 | | 0 | | 0.04 |  |
| 66 | | -4.744 | | 0.119 | | 0.118 | | 0.049 |  |
| 68 | | -4.942 | | 0.071 | | 0 | | 0.015 |  |
| 75 | | -2.33 | | 0.274 | | 0 | | 0.073 |  |
| 80 | | -6.924 | | 0.021 | | 0 | | 0.006 |  |
| 82 | | -2.48 | | 0.266 | | 0 | | 0.092 |  |
| 85 | | -3.825 | | 0.118 | | 0 | | 0.025 |  |
| 86 | | -4.405 | | 0.16 | | 0 | | 0.061 |  |
| 87 | | -4.494 | | 0.116 | | 0 | | 0.028 |  |
| 89 | | -4.434 | | 0.158 | | 0 | | 0.057 |  |
| 98 | | -2.306 | | 0.276 | | 0 | | 0.091 |  |
| 104 | | -3.825 | | 0.111 | | 0 | | 0.029 |  |
| 105 | | -4.318 | | 0.087 | | 0 | | 0.031 |  |
| 112 | | -2.311 | | 0.289 | | 0 | | 0.096 |  |
| 114 | | -2.767 | | 0.27 | | 0 | | 0.019 |  |
| 122 | | -2.33 | | 0.274 | | 0 | | 0.072 |  |
| 124 | | -2.33 | | 0.274 | | 0 | | 0.08 |  |
| 132 | | -2.486 | | 0.264 | | 0 | | 0.092 |  |
| 133 | | -2.1 | | 0.304 | | 0 | | 0.099 |  |
| 135 | | -4.616 | | 0.076 | | 0 | | 0.022 |  |
| 141 | | -4.96 | | 0.126 | | 0 | | 0.033 |  |
| 142 | | -3.825 | | 0.118 | | 0 | | 0.026 |  |
| 145 | | -3.825 | | 0.111 | | 0 | | 0.031 |  |
| 147 | | -4.526 | | 0.114 | | 0 | | 0.025 |  |
| 148 | | -4.398 | | 0.09 | | 0 | | 0.031 |  |
| 149 | | -4.661 | | 0.075 | | 0 | | 0.015 |  |
| 150 | | -6.602 | | 0.027 | | 0 | | 0.009 |  |
| 152 | | -2.276 | | 0.28 | | 0 | | 0.092 |  |
| 153 | | -5.738 | | 0.037 | | 0 | | 0.008 |  |
| 155 | | -6.92 | | 0.092 | | 0 | | 0.029 |  |
| 157 | | -3.825 | | 0.111 | | 0 | | 0.029 |  |
| 165 | | -13.697 | | 0.009 | | 0 | | 0.002 |  |
| 168 | | -7.651 | | 0.012 | | 0 | | 0.001 |  |
| 170 | | -3.923 | | 0.106 | | 0 | | 0.04 |  |
| 171 | | -4.528 | | 0.114 | | 0 | | 0.026 |  |
| 172 | | -5.738 | | 0.037 | | 0 | | 0.006 |  |
| 173 | | -12.331 | | 0.006 | | 0 | | 0.001 |  |
| 178 | | -5.168 | | 0.051 | | 0 | | 0.016 |  |
| 181 | | -3.825 | | 0.111 | | 0 | | 0.024 |  |
| 182 | | -3.825 | | 0.111 | | 0 | | 0.036 |  |
| 184 | | -3.825 | | 0.111 | | 0 | | 0.024 |  |
| 186 | | -6.989 | | 0.021 | | 0 | | 0.002 |  |
| 191 | | -3.825 | | 0.122 | | 0 | | 0.051 |  |
| 195 | | -5.821 | | 0.036 | | 0 | | 0.006 |  |
| 197 | | -4.181 | | 0.093 | | 0 | | 0.022 |  |
| 202 | | -4.482 | | 0.155 | | 0 | | 0.055 |  |
| 203 | | -2.092 | | 0.314 | | 0 | | 0.095 |  |
| 207 | | -5.738 | | 0.038 | | 0 | | 0.009 |  |
| 209 | | -2.328 | | 0.274 | | 0 | | 0.098 |  |
| 210 | | -2.099 | | 0.313 | | 0 | | 0.094 |  |
| 214 | | -4.257 | | 0.09 | | 0 | | 0.033 |  |
| 220 | | -4.19 | | 0.098 | | 0 | | 0.017 |  |
| 222 | | -3.812 | | 0.113 | | 0 | | 0.028 |  |
| 226 | | -4.616 | | 0.076 | | 0 | | 0.021 |  |
| 227 | | -4.737 | | 0.104 | | 0 | | 0.018 |  |
| 228 | | -5.738 | | 0.037 | | 0 | | 0.007 |  |
| 230 | | -5.71 | | 0.038 | | 0 | | 0.007 |  |
| 231 | | -5.003 | | 0.076 | | 0 | | 0.021 |  |
| 234 | | -3.825 | | 0.111 | | 0 | | 0.043 |  |
| 236 | | -5.738 | | 0.037 | | 0 | | 0.012 |  |
| 237 | | -3.93 | | 0.105 | | 0 | | 0.034 |  |
| 239 | | -6.968 | | 0.021 | | 0 | | 0.003 |  |
| 242 | | -3.383 | | 0.254 | | 0 | | 0.068 |  |
| 246 | | -4.614 | | 0.076 | | 0 | | 0.023 |  |
| 249 | | -2.066 | | 0.323 | | 0 | | 0.099 |  |
| 252 | | -2.486 | | 0.265 | | 0 | | 0.091 |  |
| 256 | | -4.658 | | 0.075 | | 0 | | 0.012 |  |
| 257 | | -7.713 | | 0.083 | | 0 | | 0.029 |  |
| 262 | | -3.825 | | 0.111 | | 0 | | 0.042 |  |
| 263 | | -2.494 | | 0.265 | | 0 | | 0.09 |  |
| 265 | | -3.825 | | 0.118 | | 0 | | 0.023 |  |
| 267 | | -3.825 | | 0.111 | | 0 | | 0.021 |  |
| 268 | | -2.329 | | 0.274 | | 0 | | 0.074 |  |
| 271 | | -3.556 | | 0.183 | | 0 | | 0.042 |  |
| 274 | | -2.462 | | 0.267 | | 0 | | 0.096 |  |
| 282 | | -3.825 | | 0.111 | | 0 | | 0.036 |  |
| 283 | | -3.825 | | 0.111 | | 0 | | 0.024 |  |
| 284 | | -5.455 | | 0.043 | | 0 | | 0.009 |  |
| 288 | | -4.403 | | 0.09 | | 0 | | 0.032 |  |
| 289 | | -9.317 | | 0.006 | | 0 | | 0.001 |  |
| 290 | | -5.738 | | 0.037 | | 0 | | 0.012 |  |
| 291 | | -7.651 | | 0.014 | | 0 | | 0.001 |  |
| 292 | | -4.448 | | 0.088 | | 0 | | 0.03 |  |
| 297 | | -2.476 | | 0.267 | | 0 | | 0.093 |  |
| 302 | | -4.189 | | 0.138 | | 0 | | 0.036 |  |
| 303 | | -2.33 | | 0.291 | | 0 | | 0.099 |  |
| 308 | | -2.496 | | 0.264 | | 0 | | 0.089 |  |
| 311 | | -3.825 | | 0.111 | | 0 | | 0.036 |  |
| 312 | | -4.616 | | 0.086 | | 0 | | 0.035 |  |
| 315 | | -4.17 | | 0.094 | | 0 | | 0.021 |  |
| 318 | | -2.328 | | 0.274 | | 0 | | 0.09 |  |
| 323 | | -2.475 | | 0.266 | | 0 | | 0.093 |  |
| 327 | | -5.738 | | 0.04 | | 0 | | 0.006 |  |
| 328 | | -5.738 | | 0.037 | | 0 | | 0.012 |  |
| 336 | | -3.825 | | 0.111 | | 0 | | 0.024 |  |
| 340 | | -3.825 | | 0.111 | | 0 | | 0.021 |  |
| 348 | | -5.738 | | 0.041 | | 0 | | 0.006 |  |
| 349 | | -3.825 | | 0.111 | | 0 | | 0.032 |  |
| 352 | | -4.615 | | 0.076 | | 0 | | 0.027 |  |
| 355 | | -3.825 | | 0.111 | | 0 | | 0.04 |  |
| 356 | | -4.614 | | 0.086 | | 0 | | 0.035 |  |
| 358 | | -6.977 | | 0.021 | | 0 | | 0.003 |  |
| 359 | | -9.564 | | 0.004 | | 0 | | 0 |  |
| 360 | | -7.651 | | 0.012 | | 0 | | 0.001 |  |
| 362 | | -4.613 | | 0.076 | | 0 | | 0.026 |  |
| 363 | | -3.805 | | 0.113 | | 0 | | 0.029 |  |
| 369 | | -5.738 | | 0.037 | | 0 | | 0.008 |  |
| 370 | | -2.093 | | 0.314 | | 0 | | 0.087 |  |
| 371 | | -4.166 | | 0.101 | | 0 | | 0.018 |  |
| 373 | | -11.476 | | 0.001 | | 0 | | 0 |  |
| 374 | | -3.825 | | 0.111 | | 0 | | 0.031 |  |
| 375 | | -4.982 | | 0.07 | | 0 | | 0.015 |  |
| 376 | | -3.825 | | 0.111 | | 0 | | 0.03 |  |
| 378 | | -3.825 | | 0.111 | | 0 | | 0.027 |  |
| 381 | | -3.825 | | 0.111 | | 0 | | 0.033 |  |
| 382 | | -3.825 | | 0.111 | | 0 | | 0.033 |  |
| 384 | | -5.719 | | 0.037 | | 0 | | 0.008 |  |
| 387 | | -3.825 | | 0.111 | | 0 | | 0.035 |  |
| 389 | | -7.306 | | 0.02 | | 0 | | 0.003 |  |
| 390 | | -8.961 | | 0.008 | | 0 | | 0.002 |  |
| 393 | | -3.234 | | 0.207 | | 0 | | 0.06 |  |
| 394 | | -3.825 | | 0.111 | | 0 | | 0.041 |  |
| 396 | | -4.962 | | 0.071 | | 0 | | 0.016 |  |
| 403 | | -4.435 | | 0.089 | | 0 | | 0.03 |  |
| 406 | | -7.651 | | 0.012 | | 0 | | 0.003 |  |
| 412 | | -3.825 | | 0.111 | | 0 | | 0.034 |  |
| 414 | | -4.97 | | 0.126 | | 0 | | 0.031 |  |
| 415 | | -3.825 | | 0.111 | | 0 | | 0.031 |  |
| 417 | | -5.896 | | 0.034 | | 0 | | 0.01 |  |
| 418 | | -3.825 | | 0.111 | | 0 | | 0.022 |  |
| 419 | | -2.33 | | 0.274 | | 0 | | 0.087 |  |
| 422 | | -5.738 | | 0.044 | | 0 | | 0.01 |  |
| 428 | | -15.302 | | 0.001 | | 0.074 | | 0 |  |
| 429 | | -2.33 | | 0.274 | | 0 | | 0.081 |  |
| 430 | | -4.944 | | 0.127 | | 0 | | 0.031 |  |
| 438 | | -3.803 | | 0.113 | | 0 | | 0.028 |  |
| 439 | | -3.825 | | 0.111 | | 0 | | 0.041 |  |
| 440 | | -4.918 | | 0.072 | | 0 | | 0.017 |  |
| 449 | | -3.949 | | 0.104 | | 0 | | 0.036 |  |
| 453 | | -2.33 | | 0.274 | | 0 | | 0.077 |  |
| 454 | | -2.475 | | 0.267 | | 0 | | 0.091 |  |
| 455 | | -7.372 | | 0.034 | | 0.111 | | 0.018 |  |
| 459 | | -8.342 | | 0.028 | | 0.088 | | 0.015 |  |
| 462 | | -2.33 | | 0.274 | | 0 | | 0.083 |  |
| 470 | | -4.922 | | 0.072 | | 0 | | 0.017 |  |
| 474 | | -6.977 | | 0.021 | | 0 | | 0.003 |  |
| 477 | | -6.016 | | 0.09 | | 0.185 | | 0.05 |  |
| 484 | | -6.061 | | 0.067 | | 0.11 | | 0.038 |  |
| 486 | | -4.613 | | 0.076 | | 0 | | 0.022 |  |
| 487 | | -5.007 | | 0.058 | | 0 | | 0.018 |  |
| 488 | | -2.491 | | 0.264 | | 0 | | 0.092 |  |
| 492 | | -3.825 | | 0.111 | | 0 | | 0.024 |  |
| 497 | | -4.147 | | 0.1 | | 0 | | 0.017 |  |
| 499 | | -3.825 | | 0.111 | | 0 | | 0.03 |  |
| 500 | | -9.564 | | 0.004 | | 0 | | 0 |  |
| 509 | | -6.986 | | 0.021 | | 0 | | 0.002 |  |
| 514 | | -3.825 | | 0.111 | | 0 | | 0.025 |  |
| 515 | | -3.825 | | 0.111 | | 0 | | 0.038 |  |
| 518 | | -4.66 | | 0.075 | | 0 | | 0.014 |  |
| 526 | | -4.512 | | 0.086 | | 0 | | 0.028 |  |
| 527 | | -6.732 | | 0.025 | | 0 | | 0.007 |  |
| 530 | | -6.027 | | 0.077 | | 0.099 | | 0.025 |  |
| 533 | | -16.739 | | 0.001 | | 0.056 | | 0 |  |
| 566 | | -4.898 | | 0.106 | | 0.147 | | 0.065 |  |
| 567 | | -4.866 | | 0.131 | | 0 | | 0.016 |  |
| 575 | | -8.956 | | 0.024 | | 0 | | 0.007 |  |
| 576 | | -2.487 | | 0.265 | | 0 | | 0.091 |  |
| 577 | | -6.782 | | 0.025 | | 0 | | 0.006 |  |
| 602 | | -3.783 | | 0.184 | | 0.136 | | 0.099 |  |
| 619 | | -5.738 | | 0.1 | | 0.238 | | 0.089 |  |
| 639 | | -2.312 | | 0.276 | | 0 | | 0.088 |  |
| 673 | | -2.331 | | 0.274 | | 0 | | 0.084 |  |
| 684 | | -4.594 | | 0.083 | | 0 | | 0.024 |  |
| 696 | | -3.825 | | 0.111 | | 0 | | 0.03 |  |
| 698 | | -2.475 | | 0.265 | | 0 | | 0.092 |  |
| 699 | | -5.738 | | 0.037 | | 0 | | 0.005 |  |
| 701 | | -6.256 | | 0.032 | | 0 | | 0.003 |  |
| 702 | | -1.913 | | 0.333 | | 0 | | 0.097 |  |
| 703 | | -3.825 | | 0.111 | | 0 | | 0.024 |  |
| 706 | | -2.307 | | 0.276 | | 0 | | 0.09 |  |
| 712 | | -2.482 | | 0.266 | | 0 | | 0.091 |  |
| 720 | | -2.307 | | 0.276 | | 0 | | 0.093 |  |
| 733 | | -4.205 | | 0.136 | | 0 | | 0.049 |  |
| 737 | | -8.356 | | 0.023 | | 0.088 | | 0.013 |  |
| 739 | | -5.738 | | 0.037 | | 0 | | 0.009 |  |
| 746 | | -7.651 | | 0.012 | | 0 | | 0.003 |  |
| 784 | | -3.825 | | 0.118 | | 0 | | 0.025 |  |
| 790 | | -5.738 | | 0.037 | | 0 | | 0.012 |  |
| 826 | | -3.825 | | 0.111 | | 0 | | 0.027 |  |
| 828 | | -2.33 | | 0.274 | | 0 | | 0.074 |  |
| 830 | | -11.566 | | 0.002 | | 0 | | 0 |  |
| 831 | | -4.649 | | 0.075 | | 0 | | 0.016 |  |
| 837 | | -4.544 | | 0.084 | | 0 | | 0.027 |  |
| 840 | | -4.083 | | 0.173 | | 0.128 | | 0.088 |  |
| 841 | | -6.441 | | 0.026 | | 0 | | 0.004 |  |
| 842 | | -3.357 | | 0.268 | | 0 | | 0.074 |  |
| 844 | | -3.825 | | 0.111 | | 0 | | 0.03 |  |
| 847 | | -2.431 | | 0.27 | | 0 | | 0.086 |  |
| 854 | | -3.825 | | 0.111 | | 0 | | 0.024 |  |
| 858 | | -2.476 | | 0.265 | | 0 | | 0.094 |  |
| 860 | | -2.33 | | 0.274 | | 0 | | 0.085 |  |
| 862 | | -2.343 | | 0.327 | | 0 | | 0.098 |  |
| 863 | | -2.398 | | 0.318 | | 0 | | 0.093 |  |
| 867 | | -9.564 | | 0.005 | | 0 | | 0 |  |
| 878 | | -4.841 | | 0.074 | | 0 | | 0.019 |  |
| 880 | | -2.33 | | 0.274 | | 0 | | 0.087 |  |
| 881 | | -2.075 | | 0.307 | | 0 | | 0.064 |  |
| 882 | | -5.738 | | 0.037 | | 0 | | 0.005 |  |
| 884 | | -5.738 | | 0.037 | | 0 | | 0.012 |  |
| 885 | | -3.825 | | 0.111 | | 0 | | 0.038 |  |
| 889 | | -2.33 | | 0.274 | | 0 | | 0.089 |  |
| 891 | | -3.825 | | 0.125 | | 0 | | 0.038 |  |
| 893 | | -4.19 | | 0.093 | | 0 | | 0.022 |  |
| 901 | | -3.825 | | 0.112 | | 0 | | 0.033 |  |
| 904 | | -4.784 | | 0.111 | | 0.127 | | 0.052 |  |
| 906 | | -9.237 | | 0.006 | | 0 | | 0.001 |  |
| 907 | | -3.825 | | 0.111 | | 0 | | 0.049 |  |
| 908 | | -7.651 | | 0.012 | | 0 | | 0.003 |  |
| 916 | | -9.01 | | 0.007 | | 0 | | 0.002 |  |
| 920 | | -6.161 | | 0.103 | | 0 | | 0.036 |  |
| 921 | | -12.578 | | 0.01 | | 0 | | 0.003 |  |
| 923 | | -4.079 | | 0.102 | | 0 | | 0.018 |  |
| 924 | | -4.657 | | 0.075 | | 0 | | 0.013 |  |
| 925 | | -3.825 | | 0.125 | | 0 | | 0.038 |  |
| 926 | | -3.825 | | 0.119 | | 0 | | 0.024 |  |
| 927 | | -2.096 | | 0.313 | | 0 | | 0.089 |  |
| 930 | | -4.782 | | 0.111 | | 0.156 | | 0.08 |  |
| 932 | | -3.825 | | 0.111 | | 0 | | 0.026 |  |
| 936 | | -5.738 | | 0.037 | | 0 | | 0.01 |  |
| 937 | | -3.825 | | 0.111 | | 0 | | 0.023 |  |
| 941 | | -4.852 | | 0.108 | | 0.116 | | 0.042 |  |
| 950 | | -3.359 | | 0.2 | | 0 | | 0.058 |  |
| 951 | | -3.825 | | 0.111 | | 0 | | 0.03 |  |
| 953 | | -2.486 | | 0.264 | | 0 | | 0.092 |  |
| 955 | | -4.952 | | 0.127 | | 0 | | 0.033 |  |
| 956 | | -3.177 | | 0.222 | | 0 | | 0.068 |  |
| 960 | | -5.666 | | 0.038 | | 0 | | 0.014 |  |
| 963 | | -2.486 | | 0.264 | | 0 | | 0.092 |  |
| 964 | | -3.825 | | 0.111 | | 0 | | 0.025 |  |
| 966 | | -4.616 | | 0.076 | | 0 | | 0.02 |  |
| 970 | | -4.614 | | 0.076 | | 0 | | 0.016 |  |
| 971 | | -7.651 | | 0.015 | | 0 | | 0.006 |  |
| 974 | | -3.825 | | 0.118 | | 0 | | 0.022 |  |
| 977 | | -3.825 | | 0.111 | | 0 | | 0.035 |  |
| 979 | | -2.33 | | 0.274 | | 0 | | 0.081 |  |
| 980 | | -5.738 | | 0.037 | | 0 | | 0.009 |  |
| 982 | | -3.825 | | 0.111 | | 0 | | 0.024 |  |
| 983 | | -3.825 | | 0.111 | | 0 | | 0.037 |  |
| 991 | | -4.176 | | 0.093 | | 0 | | 0.02 |  |
| 993 | | -3.825 | | 0.125 | | 0 | | 0.036 |  |
| 999 | | -2.33 | | 0.274 | | 0 | | 0.074 |  |
| 1001 | | -2.496 | | 0.263 | | 0 | | 0.088 |  |
| 1002 | | -4.619 | | 0.076 | | 0 | | 0.024 |  |
| 1005 | | -6.317 | | 0.049 | | 0 | | 0.014 |  |
| 1006 | | -4.782 | | 0.111 | | 0.128 | | 0.054 |  |
| 1008 | | -11.388 | | 0.001 | | 0 | | 0 |  |
| 1010 | | -2.329 | | 0.274 | | 0 | | 0.088 |  |
| 1011 | | -3.96 | | 0.104 | | 0 | | 0.036 |  |
| 1012 | | -4.42 | | 0.088 | | 0 | | 0.031 |  |
| 1013 | | -3.825 | | 0.111 | | 0 | | 0.033 |  |
| 1016 | | -4.653 | | 0.075 | | 0 | | 0.016 |  |
| 1017 | | -3.141 | | 0.226 | | 0 | | 0.074 |  |
| 1025 | | -4.432 | | 0.088 | | 0 | | 0.028 |  |
| 1026 | | -2.33 | | 0.274 | | 0 | | 0.073 |  |
| 1028 | | -5.738 | | 0.037 | | 0 | | 0.005 |  |
| 1032 | | -7.658 | | 0.012 | | 0 | | 0.001 |  |
| 1038 | | -4.661 | | 0.075 | | 0 | | 0.013 |  |
| 1040 | | -7.658 | | 0.012 | | 0 | | 0.002 |  |
| 1041 | | -11.486 | | 0.002 | | 0 | | 0 |  |
| 1045 | | -4.958 | | 0.07 | | 0 | | 0.014 |  |
| 1046 | | -3.829 | | 0.111 | | 0 | | 0.029 |  |
| 1047 | | -4.673 | | 0.13 | | 0.172 | | 0.098 |  |
| 1048 | | -5.634 | | 0.067 | | 0 | | 0.012 |  |
| 1050 | | -7.658 | | 0.012 | | 0 | | 0.002 |  |
| 1052 | | -5.706 | | 0.065 | | 0 | | 0.012 |  |
| 1053 | | -3.829 | | 0.111 | | 0 | | 0.035 |  |
| 1055 | | -3.829 | | 0.111 | | 0 | | 0.028 |  |
| 1057 | | -9.572 | | 0.004 | | 0 | | 0.001 |  |
| 1063 | | -5.743 | | 0.037 | | 0 | | 0.01 |  |
| 1070 | | -5.743 | | 0.037 | | 0 | | 0.006 |  |
| 1075 | | -5.031 | | 0.127 | | 0 | | 0.027 |  |
| 1076 | | -2.484 | | 0.265 | | 0 | | 0.091 |  |
| 1077 | | -2.332 | | 0.274 | | 0 | | 0.082 |  |
| 1078 | | -2.498 | | 0.264 | | 0 | | 0.09 |  |
| 1079 | | -9.314 | | 0.006 | | 0 | | 0.001 |  |
| 1084 | | -4.665 | | 0.075 | | 0 | | 0.017 |  |
| 1085 | | -8.852 | | 0.016 | | 0 | | 0.002 |  |
| 1090 | | -2.495 | | 0.265 | | 0 | | 0.089 |  |
| 1092 | | -9.252 | | 0.006 | | 0 | | 0.001 |  |
| 1098 | | -11.486 | | 0.001 | | 0 | | 0 |  |
| 1101 | | -2.101 | | 0.313 | | 0 | | 0.094 |  |
| 1107 | | -3.829 | | 0.111 | | 0 | | 0.024 |  |
| 1108 | | -2.411 | | 0.317 | | 0 | | 0.095 |  |
| 1109 | | -5.743 | | 0.037 | | 0 | | 0.004 |  |
| 1114 | | -5.743 | | 0.1 | | 0.177 | | 0.043 |  |
| 1116 | | -4.186 | | 0.099 | | 0 | | 0.018 |  |
| 1118 | | -4.896 | | 0.073 | | 0 | | 0.018 |  |
| 1119 | | -2.465 | | 0.268 | | 0 | | 0.095 |  |
| 1120 | | -2.4 | | 0.318 | | 0 | | 0.098 |  |
| 1122 | | -6.727 | | 0.025 | | 0 | | 0.008 |  |
| 1125 | | -7.658 | | 0.012 | | 0 | | 0.002 |  |
| 1126 | | -4.25 | | 0.132 | | 0 | | 0.046 |  |
| 1128 | | -6.959 | | 0.021 | | 0 | | 0.003 |  |
| 1132 | | -3.771 | | 0.115 | | 0 | | 0.029 |  |
| 1133 | | -3.352 | | 0.273 | | 0 | | 0.075 |  |
| 1142 | | -3.829 | | 0.111 | | 0 | | 0.03 |  |
| 1149 | | -4.532 | | 0.114 | | 0 | | 0.027 |  |
| 1156 | | -6.69 | | 0.026 | | 0 | | 0.008 |  |
| 1160 | | -5.743 | | 0.037 | | 0 | | 0.011 |  |
| 1161 | | -5.239 | | 0.13 | | 0.137 | | 0.035 |  |
| 1162 | | -7.658 | | 0.013 | | 0 | | 0.002 |  |
| 1163 | | -6.993 | | 0.021 | | 0 | | 0.002 |  |
| 1164 | | -3.829 | | 0.111 | | 0 | | 0.044 |  |
| 1165 | | -4.618 | | 0.076 | | 0 | | 0.027 |  |
| 1166 | | -4.664 | | 0.075 | | 0 | | 0.01 |  |
| 1167 | | -3.829 | | 0.111 | | 0 | | 0.022 |  |
| 1168 | | -4.664 | | 0.075 | | 0 | | 0.011 |  |
| 1169 | | -7.397 | | 0.046 | | 0 | | 0.009 |  |
| 1176 | | -3.562 | | 0.183 | | 0 | | 0.045 |  |
| 1178 | | -3.829 | | 0.111 | | 0 | | 0.026 |  |
| 1179 | | -4.754 | | 0.103 | | 0 | | 0.017 |  |
| 1180 | | -4.62 | | 0.076 | | 0 | | 0.021 |  |
| 1184 | | -5.292 | | 0.095 | | 0.105 | | 0.034 |  |
| 1185 | | -6.995 | | 0.021 | | 0 | | 0.003 |  |
| 1186 | | -5.66 | | 0.039 | | 0 | | 0.007 |  |
| 1188 | | -3.829 | | 0.111 | | 0 | | 0.028 |  |
| 1189 | | -4.662 | | 0.085 | | 0 | | 0.024 |  |
| 1191 | | -6.927 | | 0.021 | | 0 | | 0.003 |  |
| 1196 | | -3.829 | | 0.125 | | 0 | | 0.038 |  |
| 1197 | | -4.617 | | 0.076 | | 0 | | 0.022 |  |
| 1199 | | -3.829 | | 0.119 | | 0 | | 0.024 |  |
| 1200 | | -2.333 | | 0.274 | | 0 | | 0.073 |  |
| 1204 | | -5.743 | | 0.037 | | 0 | | 0.006 |  |
| 1205 | | -3.801 | | 0.116 | | 0 | | 0.035 |  |
| 1209 | | -2.498 | | 0.265 | | 0 | | 0.09 |  |
| 1212 | | -9.572 | | 0.004 | | 0 | | 0.001 |  |
| 1224 | | -4.209 | | 0.092 | | 0 | | 0.021 |  |
| 1226 | | -5.743 | | 0.037 | | 0 | | 0.007 |  |
| 1229 | | -2.331 | | 0.274 | | 0 | | 0.085 |  |
| 1235 | | -5.743 | | 0.037 | | 0 | | 0.005 |  |
| 1238 | | -9.781 | | 0.02 | | 0.107 | | 0.006 |  |
| 1243 | | -4.619 | | 0.076 | | 0 | | 0.024 |  |
| 1249 | | -6.703 | | 0.045 | | 0.123 | | 0.034 |  |
| 1254 | | -4.619 | | 0.076 | | 0 | | 0.026 |  |
| 1256 | | -3.828 | | 0.111 | | 0 | | 0.021 |  |
| 1260 | | -4.992 | | 0.125 | | 0 | | 0.03 |  |
| 1264 | | -3.829 | | 0.111 | | 0 | | 0.023 |  |
| 1278 | | -2.333 | | 0.274 | | 0 | | 0.094 |  |
| 1284 | | -7.658 | | 0.013 | | 0 | | 0.002 |  |
| 1287 | | -6.111 | | 0.065 | | 0.078 | | 0.017 |  |
| 1290 | | -7.904 | | 0.081 | | 0 | | 0.026 |  |
| 1292 | | -2.331 | | 0.283 | | 0 | | 0.084 |  |
| 1299 | | -5.743 | | 0.037 | | 0 | | 0.006 |  |
| 1300 | | -4.506 | | 0.115 | | 0 | | 0.028 |  |
| 1302 | | -7.658 | | 0.012 | | 0 | | 0.002 |  |
| 1305 | | -3.829 | | 0.113 | | 0 | | 0.029 |  |
| 1306 | | -4.62 | | 0.076 | | 0 | | 0.026 |  |
| 1314 | | -5.696 | | 0.038 | | 0 | | 0.013 |  |
| 1318 | | -4.651 | | 0.075 | | 0 | | 0.016 |  |
| 1320 | | -2.33 | | 0.274 | | 0 | | 0.078 |  |
| 1321 | | -4.506 | | 0.115 | | 0 | | 0.027 |  |
| 1325 | | -3.171 | | 0.223 | | 0 | | 0.072 |  |
| 1327 | | -2.099 | | 0.313 | | 0 | | 0.094 |  |
| 1334 | | -4.171 | | 0.099 | | 0 | | 0.016 |  |
| 1337 | | -3.825 | | 0.111 | | 0 | | 0.023 |  |
| 1338 | | -3.825 | | 0.111 | | 0 | | 0.041 |  |
| 1339 | | -3.38 | | 0.198 | | 0 | | 0.05 |  |
| 1340 | | -4.984 | | 0.125 | | 0 | | 0.031 |  |
| 1341 | | -7.651 | | 0.012 | | 0 | | 0.001 |  |
| 1343 | | -3.825 | | 0.111 | | 0 | | 0.041 |  |
| 1345 | | -4.979 | | 0.125 | | 0 | | 0.032 |  |
| 1348 | | -2.088 | | 0.314 | | 0 | | 0.095 |  |
| 1354 | | -3.926 | | 0.106 | | 0 | | 0.038 |  |
| 1356 | | -6.932 | | 0.021 | | 0 | | 0.003 |  |
| 1357 | | -2.33 | | 0.274 | | 0 | | 0.085 |  |
| 1361 | | -3.688 | | 0.12 | | 0 | | 0.03 |  |
| 1362 | | -4.613 | | 0.076 | | 0 | | 0.027 |  |
| 1363 | | -2.475 | | 0.266 | | 0 | | 0.093 |  |
| 1365 | | -3.825 | | 0.111 | | 0 | | 0.045 |  |
| 1405 | | -3.825 | | 0.111 | | 0 | | 0.023 |  |
| 1426 | | -13.343 | | 0.044 | | 0.01 | | 0.026 |  |
| 1431 | | -9.564 | | 0.004 | | 0 | | 0 |  |
| 1435 | | -7.651 | | 0.045 | | 0.148 | | 0.017 |  |
| 1442 | | -3.921 | | 0.106 | | 0 | | 0.038 |  |
| 1448 | | -4.614 | | 0.076 | | 0 | | 0.024 |  |
| 1468 | | -6.28 | | 0.066 | | 0.108 | | 0.038 |  |
| 1484 | | -2.396 | | 0.319 | | 0 | | 0.097 |  |
| 1489 | | -5.048 | | 0.099 | | 0.159 | | 0.086 |  |
| 1494 | | -3.825 | | 0.111 | | 0 | | 0.045 |  |
| 1498 | | -9.564 | | 0.004 | | 0 | | 0.001 |  |
| 1503 | | -6.984 | | 0.021 | | 0 | | 0.003 |  |
| 1512 | | -8.366 | | 0.009 | | 0 | | 0.001 |  |
| 1513 | | -4.061 | | 0.099 | | 0 | | 0.034 |  |
| 1514 | | -3.825 | | 0.113 | | 0 | | 0.031 |  |
| 1515 | | -3.825 | | 0.111 | | 0 | | 0.025 |  |
| 1517 | | -2.331 | | 0.274 | | 0 | | 0.073 |  |
| 1525 | | -3.807 | | 0.113 | | 0 | | 0.029 |  |
| 1527 | | -2.329 | | 0.274 | | 0 | | 0.074 |  |
| 1530 | | -4.615 | | 0.086 | | 0 | | 0.036 |  |
| 1533 | | -7.846 | | 0.081 | | 0 | | 0.028 |  |
| 1535 | | -5.389 | | 0.072 | | 0 | | 0.016 |  |
| 1544 | | -4.659 | | 0.075 | | 0 | | 0.015 |  |
| 1545 | | -2.33 | | 0.282 | | 0 | | 0.08 |  |
| 1546 | | -3.825 | | 0.111 | | 0 | | 0.035 |  |
| 1547 | | -2.486 | | 0.264 | | 0 | | 0.092 |  |
| 1556 | | -2.403 | | 0.318 | | 0 | | 0.097 |  |
| 1557 | | -3.582 | | 0.181 | | 0 | | 0.044 |  |
| 1561 | | -6.435 | | 0.025 | | 0 | | 0.007 |  |
| 1565 | | -6.925 | | 0.021 | | 0 | | 0.005 |  |
| 1567 | | -4.782 | | 0.111 | | 0.125 | | 0.049 |  |
| 1574 | | -2.33 | | 0.274 | | 0 | | 0.078 |  |
| 1578 | | -4.236 | | 0.134 | | 0 | | 0.036 |  |
| 1583 | | -3.825 | | 0.125 | | 0 | | 0.039 |  |
| 1586 | | -2.097 | | 0.313 | | 0 | | 0.092 |  |
| 1587 | | -7.39 | | 0.046 | | 0 | | 0.008 |  |
| 1594 | | -7.651 | | 0.012 | | 0 | | 0.004 |  |
| 1595 | | -4.671 | | 0.117 | | 0.151 | | 0.076 |  |
| 1611 | | -3.825 | | 0.112 | | 0 | | 0.032 |  |
| 1615 | | -2.33 | | 0.274 | | 0 | | 0.089 |  |
| 1616 | | -7.188 | | 0.05 | | 0 | | 0.006 |  |
| 1618 | | -6.922 | | 0.021 | | 0 | | 0.005 |  |
| 1621 | | -3.825 | | 0.111 | | 0 | | 0.022 |  |
| 1623 | | -5.738 | | 0.037 | | 0 | | 0.005 |  |
| 1625 | | -4.939 | | 0.127 | | 0 | | 0.033 |  |
| 1632 | | -4.98 | | 0.07 | | 0 | | 0.016 |  |
| 1638 | | -7.568 | | 0.084 | | 0 | | 0.027 |  |
| 1639 | | -2.487 | | 0.265 | | 0 | | 0.091 |  |
| 1644 | | -3.825 | | 0.111 | | 0 | | 0.012 |  |
| 1646 | | -4.464 | | 0.156 | | 0 | | 0.058 |  |
| 1651 | | -5.738 | | 0.037 | | 0 | | 0.008 |  |
| 1654 | | -3.825 | | 0.119 | | 0 | | 0.024 |  |
| 1657 | | -4.657 | | 0.075 | | 0 | | 0.012 |  |
| 1659 | | -3.825 | | 0.111 | | 0 | | 0.038 |  |
| 1661 | | -4.656 | | 0.075 | | 0 | | 0.016 |  |
| 1663 | | -13.3 | | 0 | | 0 | | 0 |  |
| 1665 | | -3.825 | | 0.111 | | 0 | | 0.025 |  |
| 1671 | | -5.738 | | 0.043 | | 0 | | 0.016 |  |
| 1673 | | -2.442 | | 0.27 | | 0 | | 0.096 |  |
| 1681 | | -3.61 | | 0.178 | | 0 | | 0.04 |  |
| 1687 | | -4.647 | | 0.085 | | 0 | | 0.017 |  |
| 1693 | | -8.062 | | 0.079 | | 0 | | 0.028 |  |
| 1695 | | -9.564 | | 0.004 | | 0 | | 0.001 |  |
| 1696 | | -5.738 | | 0.037 | | 0 | | 0.011 |  |
| 1697 | | -2.331 | | 0.274 | | 0 | | 0.072 |  |
| 1698 | | -3.584 | | 0.181 | | 0 | | 0.043 |  |
| 1700 | | -4.621 | | 0.076 | | 0 | | 0.021 |  |
| 1703 | | -3.825 | | 0.125 | | 0 | | 0.037 |  |
| 1706 | | -8.006 | | 0.08 | | 0 | | 0.027 |  |
| 1709 | | -4.931 | | 0.128 | | 0 | | 0.034 |  |
| 1711 | | -3.145 | | 0.226 | | 0 | | 0.091 |  |
| 1713 | | -4.66 | | 0.085 | | 0 | | 0.023 |  |
| 1715 | | -5.738 | | 0.037 | | 0 | | 0.009 |  |
| 1716 | | -5.738 | | 0.038 | | 0 | | 0.008 |  |
| 1721 | | -4.412 | | 0.089 | | 0 | | 0.032 |  |
| 1725 | | -4.418 | | 0.088 | | 0 | | 0.032 |  |
| 1727 | | -9.564 | | 0.005 | | 0 | | 0 |  |
| 1728 | | -4.66 | | 0.085 | | 0 | | 0.02 |  |
| 1732 | | -2.33 | | 0.274 | | 0 | | 0.085 |  |
| 1736 | | -4.171 | | 0.093 | | 0 | | 0.023 |  |
| 1741 | | -5.738 | | 0.037 | | 0 | | 0.009 |  |
| 1743 | | -2.474 | | 0.266 | | 0 | | 0.093 |  |
| 1747 | | -7.651 | | 0.012 | | 0 | | 0.002 |  |
| 1750 | | -2.33 | | 0.274 | | 0 | | 0.085 |  |
| 1751 | | -9.6 | | 0.019 | | 0.169 | | 0.02 |  |
| 1754 | | -2.476 | | 0.265 | | 0 | | 0.094 |  |
| 1759 | | -2.329 | | 0.274 | | 0 | | 0.074 |  |
| 1763 | | -5.738 | | 0.037 | | 0 | | 0.006 |  |
| 1764 | | -3.825 | | 0.111 | | 0 | | 0.024 |  |
| 1767 | | -4.983 | | 0.07 | | 0 | | 0.014 |  |
| 1768 | | -5.738 | | 0.037 | | 0 | | 0.012 |  |
| 1769 | | -6.656 | | 0.026 | | 0 | | 0.008 |  |
| 1773 | | -3.825 | | 0.111 | | 0 | | 0.04 |  |
| 1775 | | -8.245 | | 0.01 | | 0 | | 0 |  |
| 1777 | | -7.447 | | 0.019 | | 0 | | 0.003 |  |
| 1778 | | -3.825 | | 0.111 | | 0 | | 0.024 |  |
| 1784 | | -5.738 | | 0.038 | | 0 | | 0.009 |  |
| 1786 | | -4.642 | | 0.144 | | 0 | | 0.029 |  |
| 1792 | | -3.825 | | 0.111 | | 0 | | 0.04 |  |
| 1800 | | -5.738 | | 0.037 | | 0 | | 0.005 |  |
| 1804 | | -3.825 | | 0.111 | | 0 | | 0.029 |  |
| 1805 | | -4.65 | | 0.075 | | 0 | | 0.014 |  |
| 1806 | | -4.658 | | 0.075 | | 0 | | 0.017 |  |
| 1807 | | -2.33 | | 0.274 | | 0 | | 0.084 |  |
| 1810 | | -4.782 | | 0.111 | | 0.166 | | 0.09 |  |
| 1811 | | -4.659 | | 0.08 | | 0 | | 0.014 |  |
| 1812 | | -3.825 | | 0.111 | | 0 | | 0.029 |  |
| 1813 | | -8.327 | | 0.023 | | 0.092 | | 0.015 |  |
| 1819 | | -2.15 | | 0.297 | | 0 | | 0.099 |  |
| 1820 | | -2.331 | | 0.282 | | 0 | | 0.083 |  |
| 1821 | | -2.307 | | 0.276 | | 0 | | 0.093 |  |
| 1825 | | -4.216 | | 0.136 | | 0 | | 0.039 |  |
| 1829 | | -4.615 | | 0.076 | | 0 | | 0.016 |  |
| 1830 | | -9.564 | | 0.004 | | 0 | | 0.001 |  |
| 1833 | | -4.982 | | 0.125 | | 0 | | 0.032 |  |
| 1838 | | -3.825 | | 0.111 | | 0 | | 0.031 |  |
| 1842 | | -5.738 | | 0.037 | | 0 | | 0.007 |  |
| 1843 | | -2.33 | | 0.282 | | 0 | | 0.084 |  |
| 1844 | | -3.825 | | 0.111 | | 0 | | 0.023 |  |
| 1848 | | -7.651 | | 0.012 | | 0 | | 0.004 |  |
| 1850 | | -5.738 | | 0.037 | | 0 | | 0.008 |  |
| 1851 | | -5.738 | | 0.037 | | 0 | | 0.006 |  |
| 1855 | | -4.653 | | 0.08 | | 0 | | 0.014 |  |
| 1856 | | -2.33 | | 0.274 | | 0 | | 0.095 |  |
| 1860 | | -4.982 | | 0.07 | | 0 | | 0.016 |  |
| 1862 | | -2.33 | | 0.274 | | 0 | | 0.086 |  |
| 1864 | | -3.825 | | 0.111 | | 0 | | 0.037 |  |
| 1866 | | -2.33 | | 0.274 | | 0 | | 0.088 |  |
| 1868 | | -3.825 | | 0.111 | | 0 | | 0.023 |  |
| 1871 | | -7.503 | | 0.027 | | 0 | | 0.005 |  |
| 1876 | | -2.306 | | 0.276 | | 0 | | 0.093 |  |
| 1881 | | -3.948 | | 0.104 | | 0 | | 0.037 |  |
| 1886 | | -2.33 | | 0.274 | | 0 | | 0.085 |  |
| 1888 | | -4.527 | | 0.114 | | 0 | | 0.026 |  |
| 1891 | | -4.775 | | 0.075 | | 0 | | 0.016 |  |
| 1894 | | -5.738 | | 0.037 | | 0 | | 0.012 |  |
| 1895 | | -2.331 | | 0.274 | | 0 | | 0.08 |  |
| 1897 | | -4.614 | | 0.084 | | 0 | | 0.023 |  |
| 1898 | | -3.829 | | 0.111 | | 0 | | 0.025 |  |
| 1901 | | -4.97 | | 0.071 | | 0 | | 0.016 |  |
| 1906 | | -3.829 | | 0.111 | | 0 | | 0.025 |  |
| 1907 | | -4.956 | | 0.103 | | 0.17 | | 0.097 |  |
| 1908 | | -4.62 | | 0.146 | | 0 | | 0.033 |  |
| 1910 | | -3.829 | | 0.118 | | 0 | | 0.024 |  |
| 1913 | | -4.975 | | 0.126 | | 0 | | 0.033 |  |
| 1919 | | -6.933 | | 0.025 | | 0 | | 0.008 |  |
| 1924 | | -3.829 | | 0.111 | | 0 | | 0.033 |  |
| 1925 | | -3.477 | | 0.191 | | 0 | | 0.068 |  |
| 1937 | | -3.829 | | 0.111 | | 0 | | 0.021 |  |
| 1938 | | -3.829 | | 0.111 | | 0 | | 0.024 |  |
| 1939 | | -4.664 | | 0.075 | | 0 | | 0.014 |  |
| 1946 | | -2.496 | | 0.264 | | 0 | | 0.086 |  |
| 1950 | | -3.829 | | 0.111 | | 0 | | 0.024 |  |
| 1951 | | -4.52 | | 0.085 | | 0 | | 0.024 |  |
| 1960 | | -3.931 | | 0.105 | | 0 | | 0.04 |  |
| 1963 | | -3.829 | | 0.111 | | 0 | | 0.035 |  |
| 1968 | | -3.829 | | 0.111 | | 0 | | 0.04 |  |
| 1976 | | -10.67 | | 0.007 | | 0.055 | | 0.002 |  |
| 1978 | | -8.615 | | 0.018 | | 0.082 | | 0.006 |  |
| 1981 | | -7.474 | | 0.032 | | 0.102 | | 0.02 |  |
| 1984 | | -4.786 | | 0.111 | | 0.155 | | 0.079 |  |
| 1988 | | -5.646 | | 0.067 | | 0 | | 0.012 |  |
| 1990 | | -5.262 | | 0.083 | | 0 | | 0.026 |  |
| 1993 | | -4.622 | | 0.084 | | 0 | | 0.021 |  |
| 1998 | | -4.62 | | 0.086 | | 0 | | 0.035 |  |
| 2010 | | -4.707 | | 0.115 | | 0.168 | | 0.093 |  |
| 2016 | | -4.396 | | 0.123 | | 0 | | 0.032 |  |
| 2017 | | -5.743 | | 0.037 | | 0 | | 0.005 |  |
| 2018 | | -3.829 | | 0.118 | | 0 | | 0.025 |  |
| 2022 | | -13.41 | | 0.001 | | 0 | | 0 |  |
| 2024 | | -3.831 | | 0.111 | | 0 | | 0.03 |  |
| 2026 | | -3.831 | | 0.125 | | 0 | | 0.034 |  |
| 2030 | | -6.705 | | 0.045 | | 0.126 | | 0.036 |  |
| 2041 | | -8.45 | | 0.022 | | 0.059 | | 0.004 |  |
| 2046 | | -2.334 | | 0.274 | | 0 | | 0.086 |  |
| 2069 | | -3.782 | | 0.114 | | 0 | | 0.033 |  |
| 2079 | | -4.527 | | 0.114 | | 0 | | 0.025 |  |
| 2086 | | -5.025 | | 0.142 | | 0 | | 0.028 |  |
| 2094 | | -2.333 | | 0.274 | | 0 | | 0.088 |  |
| 2102 | | -7.393 | | 0.019 | | 0 | | 0.003 |  |
| 2107 | | -4.989 | | 0.07 | | 0 | | 0.014 |  |
| 2108 | | -5.747 | | 0.044 | | 0 | | 0.011 |  |
| 2110 | | -5.747 | | 0.037 | | 0 | | 0.009 |  |
| 2118 | | -3.831 | | 0.111 | | 0 | | 0.041 |  |
| 2119 | | -3.396 | | 0.196 | | 0 | | 0.054 |  |
| 2121 | | -3.831 | | 0.111 | | 0 | | 0.038 |  |
| 2124 | | -4.93 | | 0.071 | | 0 | | 0.018 |  |
| 2131 | | -3.825 | | 0.111 | | 0 | | 0.034 |  |
| 2137 | | -2.462 | | 0.267 | | 0 | | 0.096 |  |
| 2138 | | -3.14 | | 0.226 | | 0 | | 0.074 |  |
| 2142 | | -2.493 | | 0.264 | | 0 | | 0.088 |  |
| 2144 | | -2.33 | | 0.274 | | 0 | | 0.095 |  |
| 2145 | | -3.938 | | 0.105 | | 0 | | 0.037 |  |
| 2147 | | -7.431 | | 0.045 | | 0 | | 0.006 |  |
| 2152 | | -6.924 | | 0.021 | | 0 | | 0.006 |  |
| 2153 | | -2.331 | | 0.274 | | 0 | | 0.085 |  |
| 2156 | | -7.689 | | 0.012 | | 0 | | 0.002 |  |
| 2157 | | -4.9 | | 0.073 | | 0 | | 0.018 |  |
| 2159 | | -4.614 | | 0.076 | | 0 | | 0.02 |  |
| 2161 | | -5.683 | | 0.04 | | 0 | | 0.009 |  |
| 2166 | | -6.922 | | 0.024 | | 0 | | 0.005 |  |
| 2168 | | -6.928 | | 0.021 | | 0 | | 0.004 |  |
| 2169 | | -5.738 | | 0.037 | | 0 | | 0.006 |  |
| 2171 | | -2.325 | | 0.274 | | 0 | | 0.078 |  |
| 2174 | | -8.938 | | 0.007 | | 0 | | 0.002 |  |
| 2178 | | -5.738 | | 0.037 | | 0 | | 0.006 |  |
| 2181 | | -4.638 | | 0.082 | | 0 | | 0.017 |  |
| 2188 | | -2.495 | | 0.263 | | 0 | | 0.081 |  |
| 2196 | | -7.662 | | 0.012 | | 0 | | 0.002 |  |
| 2203 | | -5.747 | | 0.037 | | 0 | | 0.012 |  |
| 2204 | | -14.339 | | 0.001 | | 0.041 | | 0 |  |
| 2206 | | -3.23 | | 0.234 | | 0.139 | | 0.099 |  |
| 2229 | | -5.747 | | 0.042 | | 0 | | 0.009 |  |
| 2230 | | -7.662 | | 0.012 | | 0 | | 0.002 |  |
| 2232 | | -4.704 | | 0.106 | | 0 | | 0.021 |  |
| 2235 | | -3.831 | | 0.111 | | 0 | | 0.028 |  |
| 2241 | | -4.707 | | 0.128 | | 0.156 | | 0.081 |  |
| 2249 | | -4.789 | | 0.111 | | 0.125 | | 0.05 |  |
| 2253 | | -7.662 | | 0.014 | | 0 | | 0.001 |  |
| 2256 | | -3.831 | | 0.111 | | 0 | | 0.023 |  |
| 2259 | | -4.662 | | 0.085 | | 0 | | 0.025 |  |
| 2260 | | -3.412 | | 0.195 | | 0 | | 0.055 |  |
| 2272 | | -10.519 | | 0.007 | | 0.08 | | 0.004 |  |
| 2280 | | -7.897 | | 0.081 | | 0 | | 0.027 |  |
| 2285 | | -3.406 | | 0.141 | | 0 | | 0.053 |  |
| 2288 | | -6.999 | | 0.021 | | 0 | | 0.003 |  |
| 2291 | | -5.747 | | 0.037 | | 0 | | 0.005 |  |
| 2292 | | -2.412 | | 0.317 | | 0 | | 0.094 |  |
| 2296 | | -3.831 | | 0.111 | | 0 | | 0.035 |  |
| 2302 | | -5.742 | | 0.101 | | 0.231 | | 0.08 |  |
| 2305 | | -7.662 | | 0.012 | | 0 | | 0.004 |  |
| 2307 | | -4.789 | | 0.111 | | 0.154 | | 0.078 |  |
| 2312 | | -6.881 | | 0.093 | | 0 | | 0.029 |  |
| 2313 | | -3.831 | | 0.111 | | 0 | | 0.022 |  |
| 2315 | | -7.662 | | 0.012 | | 0 | | 0.002 |  |
| 2323 | | -2.334 | | 0.282 | | 0 | | 0.082 |  |
| 2324 | | -3.831 | | 0.111 | | 0 | | 0.029 |  |
| 2328 | | -3.831 | | 0.111 | | 0 | | 0.033 |  |
| 2331 | | -3.604 | | 0.179 | | 0 | | 0.041 |  |
| 2343 | | -3.143 | | 0.227 | | 0 | | 0.073 |  |
| 2346 | | -5.747 | | 0.037 | | 0 | | 0.007 |  |
| 2347 | | -5.337 | | 0.08 | | 0 | | 0.025 |  |
| 2348 | | -5.747 | | 0.04 | | 0 | | 0.006 |  |
| 2350 | | -2.333 | | 0.274 | | 0 | | 0.095 |  |
| 2359 | | -4.923 | | 0.072 | | 0 | | 0.016 |  |
| 2360 | | -6.712 | | 0.026 | | 0 | | 0.007 |  |
| 2369 | | -6.084 | | 0.097 | | 0.108 | | 0.032 |  |
| 2370 | | -8.64 | | 0.023 | | 0.08 | | 0.01 |  |
| 2374 | | -7.434 | | 0.086 | | 0 | | 0.03 |  |
| 2376 | | -4.662 | | 0.075 | | 0 | | 0.016 |  |
| 2378 | | -7.656 | | 0.012 | | 0 | | 0.001 |  |
| 2379 | | -3.831 | | 0.111 | | 0 | | 0.029 |  |
| 2380 | | -10.628 | | 0.008 | | 0.072 | | 0.005 |  |
| 2381 | | -7.185 | | 0.089 | | 0 | | 0.032 |  |
| 2383 | | -2.332 | | 0.274 | | 0 | | 0.086 |  |
| 2393 | | -3.381 | | 0.198 | | 0 | | 0.058 |  |
| 2395 | | -3.831 | | 0.111 | | 0 | | 0.029 |  |
| 2399 | | -6.732 | | 0.026 | | 0 | | 0.008 |  |
| 2407 | | -7.662 | | 0.015 | | 0 | | 0.004 |  |
| 2415 | | -3.831 | | 0.111 | | 0 | | 0.027 |  |
| 2425 | | -6.99 | | 0.021 | | 0 | | 0.004 |  |
| 2427 | | -5.747 | | 0.041 | | 0 | | 0.005 |  |
| 2431 | | -3.831 | | 0.111 | | 0 | | 0.024 |  |
| 2433 | | -4.663 | | 0.085 | | 0 | | 0.023 |  |
| 2447 | | -4.987 | | 0.125 | | 0 | | 0.032 |  |
| 2450 | | -6.094 | | 0.105 | | 0 | | 0.038 |  |
| 2451 | | -9.578 | | 0.005 | | 0 | | 0.001 |  |
| 2452 | | -2.332 | | 0.274 | | 0 | | 0.098 |  |
| 2454 | | -4.633 | | 0.086 | | 0 | | 0.031 |  |
| 2460 | | -2.328 | | 0.274 | | 0 | | 0.093 |  |
| 2470 | | -3.831 | | 0.111 | | 0 | | 0.023 |  |
| 2477 | | -3.801 | | 0.113 | | 0 | | 0.032 |  |
| 2480 | | -7.662 | | 0.012 | | 0 | | 0.001 |  |
| 2481 | | -7.662 | | 0.012 | | 0 | | 0.002 |  |
| 2501 | | -7.658 | | 0.012 | | 0 | | 0.002 |  |
| 2505 | | -3.829 | | 0.111 | | 0 | | 0.041 |  |
| 2509 | | -5.741 | | 0.1 | | 0.236 | | 0.091 |  |
| 2513 | | -3.829 | | 0.125 | | 0 | | 0.038 |  |
| 2515 | | -5.744 | | 0.041 | | 0 | | 0.006 |  |
| 2519 | | -5.936 | | 0.059 | | 0 | | 0.006 |  |
| 2521 | | -4.209 | | 0.092 | | 0 | | 0.021 |  |
| 2532 | | -3.807 | | 0.113 | | 0 | | 0.033 |  |
| 2535 | | -3.836 | | 0.111 | | 0 | | 0.04 |  |
| 2536 | | -2.337 | | 0.274 | | 0 | | 0.086 |  |
| 2553 | | -4.672 | | 0.075 | | 0 | | 0.012 |  |
| 2555 | | -5.754 | | 0.038 | | 0 | | 0.012 |  |
| 2556 | | -5.02 | | 0.062 | | 0 | | 0.018 |  |
| 2560 | | -5.754 | | 0.037 | | 0 | | 0.008 |  |
| 2563 | | -4.672 | | 0.085 | | 0 | | 0.023 |  |
| 2578 | | -2.336 | | 0.274 | | 0 | | 0.095 |  |
| 2579 | | -4.202 | | 0.098 | | 0 | | 0.017 |  |
| 2581 | | -3.836 | | 0.111 | | 0 | | 0.025 |  |
| 2583 | | -6.689 | | 0.026 | | 0 | | 0.008 |  |
| 2585 | | -4.203 | | 0.098 | | 0 | | 0.017 |  |
| 2589 | | -5.276 | | 0.083 | | 0 | | 0.02 |  |
| 2590 | | -5.693 | | 0.038 | | 0 | | 0.008 |  |
| 2592 | | -2.494 | | 0.265 | | 0 | | 0.091 |  |
| 2602 | | -4.672 | | 0.08 | | 0 | | 0.013 |  |
| 2603 | | -4.633 | | 0.076 | | 0 | | 0.022 |  |
| 2605 | | -7.003 | | 0.021 | | 0 | | 0.004 |  |
| 2608 | | -6.059 | | 0.067 | | 0.133 | | 0.058 |  |
| 2609 | | -4.435 | | 0.089 | | 0 | | 0.031 |  |
| 2615 | | -3.836 | | 0.111 | | 0 | | 0.029 |  |
| 2619 | | -3.836 | | 0.111 | | 0 | | 0.03 |  |
| 2630 | | -6.941 | | 0.021 | | 0 | | 0.005 |  |
| 2634 | | -5.95 | | 0.037 | | 0 | | 0.006 |  |
| 2639 | | -5.754 | | 0.044 | | 0 | | 0.01 |  |
| 2640 | | -3.836 | | 0.111 | | 0 | | 0.043 |  |
| 2641 | | -9.264 | | 0.006 | | 0 | | 0.001 |  |
| 2649 | | -2.337 | | 0.274 | | 0 | | 0.088 |  |
| 2651 | | -2.337 | | 0.274 | | 0 | | 0.08 |  |
| 2652 | | -6.941 | | 0.021 | | 0 | | 0.003 |  |
| 2655 | | -5.754 | | 0.037 | | 0 | | 0.008 |  |
| 2660 | | -3.836 | | 0.111 | | 0 | | 0.032 |  |
| 2662 | | -2.107 | | 0.303 | | 0 | | 0.098 |  |
| 2664 | | -11.268 | | 0.002 | | 0 | | 0 |  |
| 2665 | | -4.665 | | 0.085 | | 0 | | 0.024 |  |
| 2667 | | -3.36 | | 0.272 | | 0 | | 0.075 |  |
| 2670 | | -2.335 | | 0.274 | | 0 | | 0.09 |  |
| 2676 | | -2.329 | | 0.274 | | 0 | | 0.068 |  |
| 2678 | | -9.589 | | 0.004 | | 0 | | 0.001 |  |
| 2679 | | -2.405 | | 0.318 | | 0 | | 0.096 |  |
| 2686 | | -4.175 | | 0.099 | | 0 | | 0.018 |  |
| 2689 | | -4.933 | | 0.072 | | 0 | | 0.016 |  |
| 2694 | | -4.426 | | 0.159 | | 0 | | 0.059 |  |
| 2697 | | -4.197 | | 0.093 | | 0 | | 0.022 |  |
| 2698 | | -9.263 | | 0.006 | | 0 | | 0.001 |  |
| 2701 | | -4.986 | | 0.07 | | 0 | | 0.015 |  |
| 2703 | | -3.836 | | 0.111 | | 0 | | 0.034 |  |
| 2704 | | -4.626 | | 0.076 | | 0 | | 0.017 |  |
| 2709 | | -3.836 | | 0.111 | | 0 | | 0.035 |  |
| 2714 | | -3.836 | | 0.111 | | 0 | | 0.036 |  |
| 2717 | | -6.065 | | 0.067 | | 0.123 | | 0.049 |  |
| 2721 | | -4.668 | | 0.085 | | 0 | | 0.024 |  |
| 2726 | | -4.958 | | 0.071 | | 0 | | 0.016 |  |
| 2727 | | -6.198 | | 0.057 | | 0.116 | | 0.028 |  |
| 2728 | | -4.21 | | 0.137 | | 0 | | 0.047 |  |
| 2729 | | -7.006 | | 0.021 | | 0 | | 0.003 |  |
| 2732 | | -6.625 | | 0.064 | | 0 | | 0.02 |  |
| 2734 | | -5.017 | | 0.132 | | 0 | | 0.027 |  |
| 2737 | | -3.836 | | 0.111 | | 0 | | 0.029 |  |
| 2739 | | -3.788 | | 0.114 | | 0 | | 0.028 |  |
| 2741 | | -2.103 | | 0.313 | | 0 | | 0.094 |  |
| 2742 | | -2.336 | | 0.274 | | 0 | | 0.088 |  |
| 2747 | | -5.754 | | 0.041 | | 0 | | 0.006 |  |
| 2748 | | -4.641 | | 0.076 | | 0 | | 0.021 |  |
| 2750 | | -4.026 | | 0.101 | | 0 | | 0.037 |  |
| 2752 | | -4.666 | | 0.075 | | 0 | | 0.012 |  |
| 2759 | | -4.982 | | 0.07 | | 0 | | 0.016 |  |
| 2762 | | -2.399 | | 0.319 | | 0 | | 0.099 |  |
| 2763 | | -4.628 | | 0.076 | | 0 | | 0.023 |  |
| 2766 | | -3.792 | | 0.184 | | 0.137 | | 0.098 |  |
| 2770 | | -3.836 | | 0.111 | | 0 | | 0.022 |  |
| 2784 | | -2.336 | | 0.274 | | 0 | | 0.085 |  |
| 2798 | | -3.825 | | 0.111 | | 0 | | 0.025 |  |
| 2804 | | -3.825 | | 0.111 | | 0 | | 0.025 |  |
| 2813 | | -6.365 | | 0.027 | | 0 | | 0.004 |  |
| 2822 | | -4.913 | | 0.072 | | 0 | | 0.018 |  |
| 2823 | | -3.335 | | 0.257 | | 0 | | 0.073 |  |
| 2824 | | -3.775 | | 0.115 | | 0 | | 0.03 |  |
| 2826 | | -6.107 | | 0.065 | | 0.087 | | 0.022 |  |
| 2830 | | -7.651 | | 0.012 | | 0 | | 0.002 |  |
| 2836 | | -2.331 | | 0.274 | | 0 | | 0.086 |  |
| 2838 | | -3.597 | | 0.18 | | 0 | | 0.039 |  |
| 2839 | | -3.825 | | 0.111 | | 0 | | 0.039 |  |
| 2840 | | -5.88 | | 0.037 | | 0 | | 0.008 |  |
| 2842 | | -6.957 | | 0.021 | | 0 | | 0.001 |  |
| 2845 | | -2.33 | | 0.282 | | 0 | | 0.084 |  |
| 2850 | | -4.782 | | 0.111 | | 0.159 | | 0.082 |  |
| 2855 | | -4.659 | | 0.085 | | 0 | | 0.023 |  |
| 2859 | | -4.661 | | 0.075 | | 0 | | 0.017 |  |
| 2860 | | -2.496 | | 0.264 | | 0 | | 0.09 |  |
| 2872 | | -8.26 | | 0.01 | | 0 | | 0.001 |  |
| 2873 | | -3.312 | | 0.201 | | 0 | | 0.06 |  |
| 2876 | | -2.329 | | 0.274 | | 0 | | 0.085 |  |
| 2877 | | -5.738 | | 0.037 | | 0 | | 0.006 |  |
| 2888 | | -4.565 | | 0.112 | | 0 | | 0.025 |  |
| 2889 | | -2.33 | | 0.274 | | 0 | | 0.088 |  |
| 2897 | | -3.778 | | 0.184 | | 0.138 | | 0.1 |  |
| 2900 | | -6.935 | | 0.021 | | 0 | | 0.004 |  |
| 2901 | | -3.825 | | 0.119 | | 0 | | 0.025 |  |
| 2902 | | -6.986 | | 0.021 | | 0 | | 0.003 |  |
| 2906 | | -3.551 | | 0.184 | | 0 | | 0.047 |  |
| 2908 | | -2.33 | | 0.274 | | 0 | | 0.088 |  |
| 2915 | | -3.825 | | 0.111 | | 0 | | 0.023 |  |
| 2916 | | -3.15 | | 0.225 | | 0 | | 0.08 |  |
| 2920 | | -3.825 | | 0.111 | | 0 | | 0.033 |  |
| 2934 | | -5.65 | | 0.039 | | 0 | | 0.011 |  |
| 2941 | | -6.924 | | 0.021 | | 0 | | 0.005 |  |
| 2944 | | -7.651 | | 0.012 | | 0 | | 0.002 |  |
| 2945 | | -2.494 | | 0.265 | | 0 | | 0.09 |  |
| 2946 | | -4.111 | | 0.096 | | 0 | | 0.017 |  |
| 2948 | | -4.399 | | 0.09 | | 0 | | 0.032 |  |
| 2958 | | -2.476 | | 0.267 | | 0 | | 0.093 |  |
| 2960 | | -5.738 | | 0.037 | | 0 | | 0.009 |  |
| 2965 | | -3.825 | | 0.111 | | 0 | | 0.034 |  |
| 2967 | | -3.825 | | 0.111 | | 0 | | 0.022 |  |
| 2968 | | -3.193 | | 0.215 | | 0 | | 0.069 |  |
| 2970 | | -9.234 | | 0.006 | | 0 | | 0.001 |  |
| 2975 | | -4.427 | | 0.089 | | 0 | | 0.031 |  |
| 2982 | | -2.431 | | 0.272 | | 0 | | 0.085 |  |
| 2986 | | -2.328 | | 0.274 | | 0 | | 0.098 |  |
| 2987 | | -4.66 | | 0.075 | | 0 | | 0.017 |  |
| 2991 | | -3.825 | | 0.111 | | 0 | | 0.019 |  |
| 2998 | | -8.086 | | 0.079 | | 0 | | 0.028 |  |
| 3002 | | -6.889 | | 0.024 | | 0 | | 0.005 |  |
| 3003 | | -6.982 | | 0.021 | | 0 | | 0.003 |  |
| 3005 | | -3.361 | | 0.145 | | 0 | | 0.056 |  |
| 3009 | | -6.944 | | 0.021 | | 0 | | 0.004 |  |
| 3011 | | -4.782 | | 0.111 | | 0.159 | | 0.082 |  |
| 3012 | | -2.486 | | 0.264 | | 0 | | 0.092 |  |
| 3018 | | -3.825 | | 0.111 | | 0 | | 0.034 |  |
| 3021 | | -8.856 | | 0.008 | | 0 | | 0.002 |  |
| 3023 | | -4.614 | | 0.076 | | 0 | | 0.022 |  |
| 3025 | | -9.56 | | 0.018 | | 0 | | 0.004 |  |
| 3030 | | -3.932 | | 0.105 | | 0 | | 0.038 |  |
| 3039 | | -3.825 | | 0.117 | | 0 | | 0.026 |  |
| 3040 | | -5.738 | | 0.041 | | 0 | | 0.004 |  |
| 3041 | | -5.738 | | 0.037 | | 0 | | 0.009 |  |
| 3044 | | -3.825 | | 0.111 | | 0 | | 0.026 |  |
| 3049 | | -3.825 | | 0.111 | | 0 | | 0.04 |  |
| 3053 | | -3.825 | | 0.111 | | 0 | | 0.023 |  |
| 3056 | | -5.729 | | 0.038 | | 0 | | 0.007 |  |
| 3058 | | -3.825 | | 0.111 | | 0 | | 0.024 |  |
| 3062 | | -5.738 | | 0.04 | | 0 | | 0.006 |  |
| 3064 | | -3.825 | | 0.118 | | 0 | | 0.025 |  |
| 3066 | | -7.651 | | 0.012 | | 0 | | 0.002 |  |
| 3067 | | -4.653 | | 0.085 | | 0 | | 0.025 |  |
| 3068 | | -2.33 | | 0.274 | | 0 | | 0.073 |  |
| 3070 | | -2.33 | | 0.282 | | 0 | | 0.085 |  |
| 3073 | | -4.625 | | 0.076 | | 0 | | 0.019 |  |
| 3077 | | -2.33 | | 0.274 | | 0 | | 0.086 |  |
| 3082 | | -4.659 | | 0.075 | | 0 | | 0.016 |  |
| 3083 | | -2.487 | | 0.264 | | 0 | | 0.092 |  |
| 3085 | | -2.331 | | 0.274 | | 0 | | 0.092 |  |
| 3087 | | -7.651 | | 0.012 | | 0 | | 0.002 |  |
| 3088 | | -3.825 | | 0.111 | | 0 | | 0.034 |  |
| 3091 | | -6.929 | | 0.021 | | 0 | | 0.005 |  |
| 3092 | | -2.331 | | 0.274 | | 0 | | 0.073 |  |
| 3094 | | -3.343 | | 0.202 | | 0 | | 0.061 |  |
| 3097 | | -2.32 | | 0.292 | | 0 | | 0.093 |  |
| 3098 | | -3.3 | | 0.207 | | 0 | | 0.068 |  |
| 3099 | | -2.493 | | 0.264 | | 0 | | 0.087 |  |
| 3100 | | -2.33 | | 0.274 | | 0 | | 0.085 |  |
| 3103 | | -3.44 | | 0.192 | | 0 | | 0.05 |  |
| 3107 | | -3.983 | | 0.178 | | 0.137 | | 0.097 |  |
| 3111 | | -2.328 | | 0.274 | | 0 | | 0.087 |  |
| 3115 | | -5.738 | | 0.037 | | 0 | | 0.006 |  |
| 3118 | | -4.613 | | 0.076 | | 0 | | 0.026 |  |
| 3120 | | -3.779 | | 0.114 | | 0 | | 0.029 |  |
| 3122 | | -6.929 | | 0.021 | | 0 | | 0.005 |  |
| 3128 | | -4.486 | | 0.087 | | 0 | | 0.027 |  |
| 3130 | | -5.025 | | 0.055 | | 0 | | 0.018 |  |
| 3131 | | -4.184 | | 0.098 | | 0 | | 0.018 |  |
| 3135 | | -3.825 | | 0.111 | | 0 | | 0.04 |  |
| 3141 | | -6.677 | | 0.062 | | 0 | | 0.02 |  |
| 3142 | | -2.33 | | 0.274 | | 0 | | 0.095 |  |
| 3143 | | -4.614 | | 0.076 | | 0 | | 0.021 |  |
| 3150 | | -4.614 | | 0.086 | | 0 | | 0.035 |  |
| 3151 | | -3.825 | | 0.111 | | 0 | | 0.029 |  |
| 3154 | | -3.825 | | 0.112 | | 0 | | 0.022 |  |
| 3161 | | -4.619 | | 0.076 | | 0 | | 0.022 |  |
| 3163 | | -4.617 | | 0.076 | | 0 | | 0.023 |  |
| 3166 | | -4.613 | | 0.086 | | 0 | | 0.034 |  |
| 3167 | | -2.329 | | 0.274 | | 0 | | 0.073 |  |
| 3169 | | -4.538 | | 0.084 | | 0 | | 0.027 |  |
| 3170 | | -4.406 | | 0.09 | | 0 | | 0.028 |  |
| 3174 | | -4.978 | | 0.07 | | 0 | | 0.016 |  |
| 3175 | | -4.397 | | 0.09 | | 0 | | 0.032 |  |
| 3176 | | -3.825 | | 0.111 | | 0 | | 0.043 |  |
| 3178 | | -3.801 | | 0.113 | | 0 | | 0.026 |  |
| 3179 | | -4.363 | | 0.126 | | 0 | | 0.06 |  |
| 3181 | | -3.825 | | 0.111 | | 0 | | 0.023 |  |
| 3188 | | -5.068 | | 0.133 | | 0 | | 0.026 |  |
| 3190 | | -2.329 | | 0.283 | | 0 | | 0.084 |  |
| 3191 | | -4.926 | | 0.128 | | 0 | | 0.035 |  |
| 3195 | | -2.5 | | 0.264 | | 0 | | 0.09 |  |
| 3196 | | -4.619 | | 0.076 | | 0 | | 0.022 |  |
| 3197 | | -4.571 | | 0.112 | | 0 | | 0.024 |  |
| 3198 | | -8.137 | | 0.078 | | 0 | | 0.027 |  |
| 3199 | | -3.83 | | 0.111 | | 0 | | 0.029 |  |
| 3200 | | -9.314 | | 0.006 | | 0 | | 0 |  |
| 3201 | | -5.745 | | 0.041 | | 0 | | 0.006 |  |
| 3202 | | -4.202 | | 0.092 | | 0 | | 0.021 |  |
| 3210 | | -3.83 | | 0.111 | | 0 | | 0.033 |  |
| 3218 | | -5.745 | | 0.037 | | 0 | | 0.013 |  |
| 3226 | | -3.83 | | 0.111 | | 0 | | 0.023 |  |
| 3227 | | -9.574 | | 0.004 | | 0 | | 0.001 |  |
| 3228 | | -3.83 | | 0.111 | | 0 | | 0.039 |  |
| 3230 | | -5.745 | | 0.037 | | 0 | | 0.006 |  |
| 3231 | | -11.489 | | 0.001 | | 0 | | 0 |  |
| 3233 | | -4.62 | | 0.076 | | 0 | | 0.022 |  |
| 3235 | | -4.979 | | 0.07 | | 0 | | 0.016 |  |
| 3239 | | -5.734 | | 0.037 | | 0 | | 0.006 |  |
| 3244 | | -4.405 | | 0.16 | | 0 | | 0.061 |  |
| 3246 | | -7.152 | | 0.064 | | 0.204 | | 0.062 |  |
| 3249 | | -5.581 | | 0.123 | | 0.183 | | 0.07 |  |
| 3254 | | -3.339 | | 0.267 | | 0 | | 0.074 |  |
| 3258 | | -4.621 | | 0.076 | | 0 | | 0.023 |  |
| 3259 | | -5.745 | | 0.037 | | 0 | | 0.012 |  |
| 3261 | | -5.745 | | 0.037 | | 0 | | 0.008 |  |
| 3262 | | -7.659 | | 0.012 | | 0 | | 0.002 |  |
| 3264 | | -4.665 | | 0.075 | | 0 | | 0.01 |  |
| 3267 | | -5.07 | | 0.152 | | 0.147 | | 0.024 |  |
| 3281 | | -4.622 | | 0.076 | | 0 | | 0.022 |  |
| 3288 | | -4.86 | | 0.074 | | 0 | | 0.018 |  |
| 3289 | | -7.659 | | 0.012 | | 0 | | 0.002 |  |
| 3291 | | -2.332 | | 0.274 | | 0 | | 0.073 |  |
| 3296 | | -5.745 | | 0.037 | | 0 | | 0.005 |  |
| 3299 | | -2.496 | | 0.264 | | 0 | | 0.088 |  |
| 3303 | | -3.144 | | 0.226 | | 0 | | 0.092 |  |
| 3308 | | -4.408 | | 0.09 | | 0 | | 0.032 |  |
| 3309 | | -3.83 | | 0.111 | | 0 | | 0.034 |  |
| 3311 | | -11.599 | | 0.002 | | 0 | | 0 |  |
| 3313 | | -2.333 | | 0.274 | | 0 | | 0.094 |  |
| 3319 | | -14.774 | | 0 | | 0 | | 0 |  |
| 3326 | | -2.333 | | 0.274 | | 0 | | 0.094 |  |
| 3327 | | -13.933 | | 0 | | 0 | | 0 |  |
| 3336 | | -4.446 | | 0.157 | | 0 | | 0.059 |  |
| 3339 | | -3.197 | | 0.22 | | 0 | | 0.071 |  |
| 3344 | | -3.83 | | 0.111 | | 0 | | 0.035 |  |
| 3347 | | -3.83 | | 0.111 | | 0 | | 0.025 |  |
| 3350 | | -5.745 | | 0.037 | | 0 | | 0.013 |  |
| 3353 | | -4.665 | | 0.075 | | 0 | | 0.015 |  |
| 3357 | | -6.053 | | 0.067 | | 0.114 | | 0.041 |  |
| 3371 | | -5.745 | | 0.041 | | 0 | | 0.006 |  |
| 3372 | | -3.83 | | 0.118 | | 0 | | 0.026 |  |
| 3373 | | -3.83 | | 0.111 | | 0 | | 0.041 |  |
| 3374 | | -4.442 | | 0.089 | | 0 | | 0.03 |  |
| 3375 | | -3.83 | | 0.118 | | 0 | | 0.025 |  |
| 3376 | | -4.626 | | 0.086 | | 0 | | 0.035 |  |
| 3378 | | -6.723 | | 0.061 | | 0 | | 0.016 |  |
| 3385 | | -9.574 | | 0.004 | | 0 | | 0.001 |  |
| 3386 | | -7.882 | | 0.081 | | 0 | | 0.026 |  |
| 3390 | | -4.621 | | 0.086 | | 0 | | 0.036 |  |
| 3391 | | -4.539 | | 0.114 | | 0 | | 0.026 |  |
| 3392 | | -5.745 | | 0.037 | | 0 | | 0.006 |  |
| 3396 | | -6.935 | | 0.021 | | 0 | | 0.003 |  |
| 3399 | | -2.085 | | 0.315 | | 0 | | 0.096 |  |
| 3404 | | -7.659 | | 0.012 | | 0 | | 0.002 |  |
| 3405 | | -4.982 | | 0.125 | | 0 | | 0.03 |  |
| 3407 | | -9.319 | | 0.006 | | 0 | | 0 |  |
| 3408 | | -6.932 | | 0.021 | | 0 | | 0.005 |  |
| 3412 | | -5.745 | | 0.037 | | 0 | | 0.005 |  |
| 3415 | | -7.453 | | 0.019 | | 0 | | 0.002 |  |
| 3417 | | -3.83 | | 0.111 | | 0 | | 0.025 |  |
| 3418 | | -5.745 | | 0.037 | | 0 | | 0.006 |  |
| 3427 | | -4.462 | | 0.087 | | 0 | | 0.03 |  |
| 3429 | | -4.985 | | 0.07 | | 0 | | 0.014 |  |
| 3431 | | -4.666 | | 0.075 | | 0 | | 0.011 |  |
| 3432 | | -3.339 | | 0.277 | | 0 | | 0.076 |  |
| 3441 | | -3.541 | | 0.191 | | 0 | | 0.049 |  |
| 3444 | | -6.169 | | 0.032 | | 0 | | 0.004 |  |
| 3445 | | -2.4 | | 0.318 | | 0 | | 0.099 |  |
| 3448 | | -3.83 | | 0.111 | | 0 | | 0.04 |  |
| 3450 | | -2.333 | | 0.274 | | 0 | | 0.089 |  |
| 3451 | | -2.333 | | 0.274 | | 0 | | 0.089 |  |
| 3456 | | -7.43 | | 0.019 | | 0 | | 0.003 |  |
| 3459 | | -6.166 | | 0.104 | | 0 | | 0.036 |  |
| 3461 | | -3.83 | | 0.111 | | 0 | | 0.041 |  |
| 3462 | | -9.24 | | 0.006 | | 0 | | 0.001 |  |
| 3468 | | -2.496 | | 0.264 | | 0 | | 0.087 |  |
| 3472 | | -4.662 | | 0.085 | | 0 | | 0.023 |  |
| 3473 | | -6.996 | | 0.021 | | 0 | | 0.002 |  |
| 3474 | | -7.659 | | 0.014 | | 0 | | 0.001 |  |
| 3476 | | -3.597 | | 0.18 | | 0 | | 0.043 |  |
| 3477 | | -6.329 | | 0.028 | | 0 | | 0.004 |  |
| 3478 | | -5.745 | | 0.044 | | 0 | | 0.01 |  |
| 3481 | | -3.198 | | 0.219 | | 0 | | 0.082 |  |
| 3482 | | -2.465 | | 0.267 | | 0 | | 0.096 |  |
| 3485 | | -3.83 | | 0.111 | | 0 | | 0.024 |  |
| 3486 | | -5.663 | | 0.039 | | 0 | | 0.008 |  |
| 3487 | | -11.583 | | 0.002 | | 0 | | 0 |  |
| 3496 | | -2.332 | | 0.274 | | 0 | | 0.085 |  |
| 3500 | | -5.738 | | 0.037 | | 0 | | 0.003 |  |
| 3501 | | -7.651 | | 0.012 | | 0 | | 0.001 |  |
| 3503 | | -9.226 | | 0.006 | | 0 | | 0.001 |  |
| 3505 | | -2.183 | | 0.292 | | 0 | | 0.098 |  |
| 3506 | | -5.738 | | 0.044 | | 0 | | 0.01 |  |
| 3509 | | -5.639 | | 0.067 | | 0 | | 0.013 |  |
| 3510 | | -3.825 | | 0.111 | | 0 | | 0.025 |  |
| 3511 | | -4.979 | | 0.07 | | 0 | | 0.014 |  |
| 3515 | | -4.929 | | 0.071 | | 0 | | 0.017 |  |
| 3518 | | -6.927 | | 0.021 | | 0 | | 0.005 |  |
| 3519 | | -6.721 | | 0.024 | | 0 | | 0.006 |  |
| 3520 | | -5.973 | | 0.078 | | 0.151 | | 0.076 |  |
| 3521 | | -2.403 | | 0.318 | | 0 | | 0.097 |  |
| 3522 | | -3.825 | | 0.111 | | 0 | | 0.033 |  |
| 3523 | | -4.656 | | 0.085 | | 0 | | 0.023 |  |
| 3524 | | -4.782 | | 0.111 | | 0.161 | | 0.085 |  |
| 3526 | | -6.978 | | 0.021 | | 0 | | 0.003 |  |
| 3529 | | -4.574 | | 0.083 | | 0 | | 0.026 |  |
| 3531 | | -2.098 | | 0.304 | | 0 | | 0.1 |  |
| 3535 | | -2.41 | | 0.317 | | 0 | | 0.096 |  |
| 3538 | | -2.404 | | 0.317 | | 0 | | 0.097 |  |
| 3539 | | -3.825 | | 0.119 | | 0 | | 0.019 |  |
| 3542 | | -5.894 | | 0.034 | | 0 | | 0.011 |  |
| 3543 | | -2.33 | | 0.274 | | 0 | | 0.087 |  |
| 3545 | | -8.607 | | 0.018 | | 0.065 | | 0.003 |  |
| 3547 | | -3.825 | | 0.111 | | 0 | | 0.026 |  |
| 3549 | | -4.656 | | 0.075 | | 0 | | 0.014 |  |
| 3553 | | -3.829 | | 0.111 | | 0 | | 0.035 |  |
| 3554 | | -3.829 | | 0.111 | | 0 | | 0.033 |  |
| 3556 | | -7.441 | | 0.045 | | 0 | | 0.009 |  |
| 3557 | | -2.333 | | 0.274 | | 0 | | 0.084 |  |
| 3562 | | -5.743 | | 0.037 | | 0 | | 0.009 |  |
| 3565 | | -3.829 | | 0.111 | | 0 | | 0.021 |  |
| 3566 | | -4.203 | | 0.092 | | 0 | | 0.018 |  |
| 3567 | | -6.707 | | 0.045 | | 0.114 | | 0.027 |  |
| 3568 | | -3.829 | | 0.111 | | 0 | | 0.032 |  |
| 3571 | | -12.178 | | 0.011 | | 0 | | 0.002 |  |
| 3573 | | -3.829 | | 0.111 | | 0 | | 0.022 |  |
| 3577 | | -3.829 | | 0.111 | | 0 | | 0.043 |  |
| 3578 | | -2.333 | | 0.274 | | 0 | | 0.087 |  |
| 3581 | | -7.539 | | 0.013 | | 0 | | 0.002 |  |
| 3582 | | -7.658 | | 0.012 | | 0 | | 0.003 |  |
| 3583 | | -2.332 | | 0.274 | | 0 | | 0.094 |  |
| 3585 | | -7.567 | | 0.013 | | 0 | | 0.002 |  |
| 3589 | | -6.991 | | 0.021 | | 0 | | 0.003 |  |
| 3590 | | -7.658 | | 0.012 | | 0 | | 0.003 |  |
| 3596 | | -7.658 | | 0.012 | | 0 | | 0.001 |  |
| 3607 | | -5.711 | | 0.038 | | 0 | | 0.013 |  |
| 3608 | | -3.829 | | 0.119 | | 0 | | 0.025 |  |
| 3613 | | -2.332 | | 0.274 | | 0 | | 0.086 |  |
| 3617 | | -3.829 | | 0.111 | | 0 | | 0.033 |  |
| 3620 | | -9.572 | | 0.004 | | 0 | | 0 |  |
| 3623 | | -3.829 | | 0.111 | | 0 | | 0.029 |  |
| 3624 | | -5.743 | | 0.042 | | 0 | | 0.014 |  |
| 3625 | | -9.297 | | 0.007 | | 0 | | 0.001 |  |
| 3630 | | -2.332 | | 0.274 | | 0 | | 0.072 |  |
| 3631 | | -6.934 | | 0.021 | | 0 | | 0.004 |  |
| 3633 | | -4.663 | | 0.085 | | 0 | | 0.023 |  |
| 3634 | | -3.829 | | 0.112 | | 0 | | 0.03 |  |
| 3635 | | -5.743 | | 0.037 | | 0 | | 0.008 |  |
| 3639 | | -2.331 | | 0.283 | | 0 | | 0.084 |  |
| 3646 | | -3.829 | | 0.111 | | 0 | | 0.044 |  |
| 3647 | | -3.829 | | 0.111 | | 0 | | 0.031 |  |
| 3650 | | -3.978 | | 0.103 | | 0 | | 0.036 |  |
| 3651 | | -5.743 | | 0.037 | | 0 | | 0.009 |  |
| 3652 | | -3.829 | | 0.111 | | 0 | | 0.031 |  |
| 3661 | | -2.331 | | 0.274 | | 0 | | 0.088 |  |
| 3662 | | -9.572 | | 0.004 | | 0 | | 0 |  |
| 3668 | | -4.618 | | 0.076 | | 0 | | 0.026 |  |
| 3671 | | -4.945 | | 0.071 | | 0 | | 0.015 |  |
| 3672 | | -4.661 | | 0.075 | | 0 | | 0.012 |  |
| 3680 | | -13.401 | | 0 | | 0 | | 0 |  |
| 3681 | | -3.829 | | 0.111 | | 0 | | 0.023 |  |
| 3686 | | -3.766 | | 0.115 | | 0 | | 0.03 |  |
| 3691 | | -5.743 | | 0.037 | | 0 | | 0.006 |  |
| 3692 | | -4.15 | | 0.1 | | 0 | | 0.019 |  |
| 3695 | | -2.332 | | 0.274 | | 0 | | 0.088 |  |
| 3698 | | -3.214 | | 0.218 | | 0 | | 0.067 |  |
| 3699 | | -2.333 | | 0.274 | | 0 | | 0.084 |  |
| 3701 | | -7.658 | | 0.012 | | 0 | | 0.003 |  |
| 3703 | | -15.315 | | 0 | | 0 | | 0 |  |
| 3705 | | -5.743 | | 0.037 | | 0 | | 0.009 |  |
| 3709 | | -2.1 | | 0.304 | | 0 | | 0.098 |  |
| 3714 | | -4.627 | | 0.076 | | 0 | | 0.02 |  |
| 3718 | | -3.829 | | 0.111 | | 0 | | 0.034 |  |
| 3720 | | -8.087 | | 0.079 | | 0 | | 0.028 |  |
| 3721 | | -4.104 | | 0.108 | | 0 | | 0.019 |  |
| 3723 | | -2.309 | | 0.276 | | 0 | | 0.092 |  |
| 3725 | | -2.494 | | 0.264 | | 0 | | 0.084 |  |
| 3728 | | -5.743 | | 0.037 | | 0 | | 0.005 |  |
| 3732 | | -2.085 | | 0.315 | | 0 | | 0.096 |  |
| 3737 | | -3.381 | | 0.198 | | 0 | | 0.054 |  |
| 3741 | | -4.663 | | 0.075 | | 0 | | 0.014 |  |
| 3743 | | -6.612 | | 0.027 | | 0 | | 0.008 |  |
| 3745 | | -2.331 | | 0.274 | | 0 | | 0.09 |  |
| 3750 | | -4.172 | | 0.099 | | 0 | | 0.018 |  |
| 3755 | | -6.661 | | 0.026 | | 0 | | 0.008 |  |
| 3756 | | -3.829 | | 0.113 | | 0 | | 0.029 |  |
| 3758 | | -2.329 | | 0.274 | | 0 | | 0.087 |  |
| 3762 | | -6.996 | | 0.02 | | 0 | | 0.002 |  |
| 3766 | | -9.308 | | 0.006 | | 0 | | 0 |  |
| 3767 | | -5.743 | | 0.037 | | 0 | | 0.01 |  |
| 3770 | | -2.398 | | 0.319 | | 0 | | 0.092 |  |
| 3772 | | -6.273 | | 0.031 | | 0 | | 0.004 |  |
| 3774 | | -2.404 | | 0.318 | | 0 | | 0.095 |  |
| 3776 | | -9.246 | | 0.006 | | 0 | | 0.001 |  |
| 3780 | | -3.829 | | 0.111 | | 0 | | 0.024 |  |
| 3783 | | -3.829 | | 0.111 | | 0 | | 0.035 |  |
| 3790 | | -3.829 | | 0.111 | | 0 | | 0.021 |  |
| 3793 | | -3.829 | | 0.111 | | 0 | | 0.04 |  |
| 3795 | | -4.665 | | 0.075 | | 0 | | 0.013 |  |
| 3799 | | -3.829 | | 0.125 | | 0 | | 0.039 |  |
| 3802 | | -3.829 | | 0.111 | | 0 | | 0.023 |  |
| 3805 | | -12.349 | | 0.011 | | 0 | | 0.003 |  |
| 3809 | | -4.459 | | 0.088 | | 0 | | 0.03 |  |
| 3810 | | -5.743 | | 0.037 | | 0 | | 0.008 |  |
| 3811 | | -2.333 | | 0.282 | | 0 | | 0.078 |  |
| 3813 | | -5.743 | | 0.037 | | 0 | | 0.008 |  |
| 3814 | | -4.617 | | 0.084 | | 0 | | 0.023 |  |
| 3817 | | -3.829 | | 0.111 | | 0 | | 0.03 |  |
| 3820 | | -4.617 | | 0.076 | | 0 | | 0.023 |  |
| 3824 | | -2.332 | | 0.274 | | 0 | | 0.094 |  |
| 3827 | | -5.743 | | 0.037 | | 0 | | 0.011 |  |
| 3830 | | -6.927 | | 0.024 | | 0 | | 0.005 |  |
| 3832 | | -3.578 | | 0.181 | | 0 | | 0.044 |  |
| 3835 | | -4.241 | | 0.091 | | 0 | | 0.02 |  |
| 3838 | | -3.829 | | 0.118 | | 0 | | 0.025 |  |
| 3847 | | -8.584 | | 0.008 | | 0 | | 0.002 |  |
| 3849 | | -3.825 | | 0.111 | | 0 | | 0.04 |  |
| 3850 | | -7.651 | | 0.012 | | 0 | | 0.001 |  |
| 3858 | | -4.973 | | 0.07 | | 0 | | 0.015 |  |
| 3863 | | -2.495 | | 0.263 | | 0 | | 0.081 |  |
| 3865 | | -3.825 | | 0.111 | | 0 | | 0.024 |  |
| 3867 | | -3.749 | | 0.116 | | 0 | | 0.031 |  |
| 3869 | | -3.163 | | 0.221 | | 0 | | 0.07 |  |
| 3870 | | -4.946 | | 0.071 | | 0 | | 0.017 |  |
| 3872 | | -3.825 | | 0.111 | | 0 | | 0.022 |  |
| 3877 | | -3.825 | | 0.111 | | 0 | | 0.024 |  |
| 3886 | | -2.331 | | 0.274 | | 0 | | 0.084 |  |
| 3887 | | -4.616 | | 0.076 | | 0 | | 0.022 |  |
| 3895 | | -6.697 | | 0.045 | | 0.094 | | 0.016 |  |
| 3896 | | -3.402 | | 0.196 | | 0 | | 0.052 |  |
| 3906 | | -2.463 | | 0.268 | | 0 | | 0.095 |  |
| 3907 | | -4.966 | | 0.071 | | 0 | | 0.016 |  |
| 3908 | | -2.409 | | 0.317 | | 0 | | 0.097 |  |
| 3909 | | -5.013 | | 0.056 | | 0 | | 0.019 |  |
| 3917 | | -5.741 | | 0.037 | | 0 | | 0.007 |  |
| 3918 | | -5.741 | | 0.037 | | 0 | | 0.006 |  |
| 3923 | | -6.674 | | 0.066 | | 0 | | 0.027 |  |
| 3924 | | -5.006 | | 0.068 | | 0 | | 0.017 |  |
| 3925 | | -4.112 | | 0.101 | | 0 | | 0.016 |  |
| 3938 | | -7.659 | | 0.012 | | 0 | | 0.003 |  |
| 3943 | | -3.829 | | 0.111 | | 0 | | 0.022 |  |
| 3946 | | -3.829 | | 0.111 | | 0 | | 0.023 |  |
| 3948 | | -3.829 | | 0.123 | | 0 | | 0.054 |  |
| 3950 | | -2.333 | | 0.274 | | 0 | | 0.08 |  |
| 3951 | | -2.332 | | 0.274 | | 0 | | 0.088 |  |
| 3953 | | -5.744 | | 0.037 | | 0 | | 0.005 |  |
| 3954 | | -2.333 | | 0.274 | | 0 | | 0.095 |  |
| 3959 | | -4.429 | | 0.088 | | 0 | | 0.032 |  |
| 3960 | | -4.973 | | 0.07 | | 0 | | 0.015 |  |
| 3963 | | -7.659 | | 0.012 | | 0 | | 0.002 |  |
| 3964 | | -6.935 | | 0.021 | | 0 | | 0.005 |  |
| 3967 | | -6.994 | | 0.025 | | 0 | | 0.005 |  |
| 3968 | | -4.452 | | 0.157 | | 0 | | 0.058 |  |
| 3969 | | -7.459 | | 0.045 | | 0 | | 0.008 |  |
| 3970 | | -5.744 | | 0.037 | | 0 | | 0.005 |  |
| 3974 | | -11.488 | | 0.001 | | 0 | | 0 |  |
| 3980 | | -4.62 | | 0.076 | | 0 | | 0.024 |  |
| 3981 | | -5.944 | | 0.059 | | 0 | | 0.019 |  |
| 3984 | | -4.328 | | 0.123 | | 0 | | 0.042 |  |
| 3985 | | -3.829 | | 0.111 | | 0 | | 0.029 |  |
| 3988 | | -7.659 | | 0.012 | | 0 | | 0.002 |  |
| 3991 | | -7.658 | | 0.045 | | 0.172 | | 0.028 |  |
| 3993 | | -5.744 | | 0.037 | | 0 | | 0.009 |  |
| 3995 | | -4.242 | | 0.134 | | 0 | | 0.048 |  |
| 3998 | | -2.332 | | 0.274 | | 0 | | 0.089 |  |
| 4002 | | -2.332 | | 0.274 | | 0 | | 0.085 |  |
| 4003 | | -2.333 | | 0.282 | | 0 | | 0.084 |  |
| 4004 | | -4.619 | | 0.084 | | 0 | | 0.023 |  |
| 4010 | | -6.934 | | 0.021 | | 0 | | 0.005 |  |
| 4011 | | -2.331 | | 0.274 | | 0 | | 0.09 |  |
| 4015 | | -3.829 | | 0.111 | | 0 | | 0.026 |  |
| 4016 | | -5.744 | | 0.037 | | 0 | | 0.009 |  |
| 4018 | | -2.087 | | 0.315 | | 0 | | 0.096 |  |
| 4021 | | -5.744 | | 0.037 | | 0 | | 0.007 |  |
| 4027 | | -2.333 | | 0.282 | | 0 | | 0.083 |  |
| 4029 | | -2.332 | | 0.274 | | 0 | | 0.088 |  |
| 4030 | | -3.829 | | 0.111 | | 0 | | 0.021 |  |
| 4033 | | -4.66 | | 0.085 | | 0 | | 0.024 |  |
| 4034 | | -4.619 | | 0.076 | | 0 | | 0.022 |  |
| 4038 | | -4.203 | | 0.092 | | 0 | | 0.021 |  |
| 4039 | | -2.212 | | 0.289 | | 0 | | 0.098 |  |
| 4042 | | -2.332 | | 0.274 | | 0 | | 0.085 |  |
| 4045 | | -4.064 | | 0.103 | | 0 | | 0.014 |  |
| 4052 | | -3.804 | | 0.113 | | 0 | | 0.029 |  |
| 4053 | | -4.421 | | 0.089 | | 0 | | 0.032 |  |
| 4059 | | -5.744 | | 0.037 | | 0 | | 0.011 |  |
| 4063 | | -11.488 | | 0.001 | | 0 | | 0 |  |
| 4066 | | -3.829 | | 0.111 | | 0 | | 0.032 |  |
| 4068 | | -9.324 | | 0.006 | | 0 | | 0 |  |
| 4069 | | -3.829 | | 0.111 | | 0 | | 0.03 |  |
| 4072 | | -2.49 | | 0.264 | | 0 | | 0.083 |  |
| 4075 | | -3.829 | | 0.111 | | 0 | | 0.023 |  |
| 4080 | | -4.419 | | 0.089 | | 0 | | 0.03 |  |
| 4086 | | -2.489 | | 0.265 | | 0 | | 0.091 |  |
| 4087 | | -4.888 | | 0.072 | | 0 | | 0.019 |  |
| 4091 | | -2.333 | | 0.274 | | 0 | | 0.087 |  |
| 4092 | | -2.483 | | 0.266 | | 0 | | 0.092 |  |
| 4093 | | -3.829 | | 0.111 | | 0 | | 0.04 |  |
| 4094 | | -4.787 | | 0.111 | | 0.174 | | 0.1 |  |
| 4095 | | -9.573 | | 0.004 | | 0 | | 0.001 |  |
| 4098 | | -3.829 | | 0.111 | | 0 | | 0.031 |  |
| 4099 | | -2.333 | | 0.274 | | 0 | | 0.076 |  |
| 4100 | | -6.982 | | 0.021 | | 0 | | 0.015 |  |
| 4103 | | -2.333 | | 0.274 | | 0 | | 0.086 |  |
| 4104 | | -4.622 | | 0.076 | | 0 | | 0.017 |  |
| 4105 | | -2.331 | | 0.274 | | 0 | | 0.094 |  |
| 4106 | | -3.957 | | 0.104 | | 0 | | 0.037 |  |
| 4109 | | -4.665 | | 0.075 | | 0 | | 0.017 |  |
| 4111 | | -3.498 | | 0.164 | | 0 | | 0.052 |  |
| 4114 | | -7.659 | | 0.012 | | 0 | | 0.001 |  |
| 4117 | | -2.333 | | 0.274 | | 0 | | 0.093 |  |
| 4119 | | -3.829 | | 0.111 | | 0 | | 0.043 |  |
| 4124 | | -2.331 | | 0.282 | | 0 | | 0.085 |  |
| 4134 | | -4.662 | | 0.075 | | 0 | | 0.015 |  |
| 4140 | | -3.829 | | 0.119 | | 0 | | 0.024 |  |
| 4142 | | -4.628 | | 0.086 | | 0 | | 0.033 |  |
| 4146 | | -2.465 | | 0.268 | | 0 | | 0.095 |  |
| 4149 | | -6.99 | | 0.021 | | 0 | | 0.003 |  |
| 4152 | | -4.492 | | 0.116 | | 0 | | 0.028 |  |
| 4156 | | -2.332 | | 0.274 | | 0 | | 0.073 |  |
| 4157 | | -2.495 | | 0.264 | | 0 | | 0.087 |  |
| 4158 | | -3.829 | | 0.118 | | 0 | | 0.022 |  |
| 4161 | | -2.499 | | 0.263 | | 0 | | 0.09 |  |
| 4165 | | -2.333 | | 0.274 | | 0 | | 0.087 |  |
| 4168 | | -2.33 | | 0.274 | | 0 | | 0.075 |  |
| 4169 | | -6.985 | | 0.021 | | 0 | | 0.003 |  |
| 4172 | | -3.829 | | 0.111 | | 0 | | 0.029 |  |
| 4174 | | -2.068 | | 0.323 | | 0 | | 0.099 |  |
| 4176 | | -2.332 | | 0.274 | | 0 | | 0.095 |  |
| 4179 | | -4.665 | | 0.075 | | 0 | | 0.005 |  |
| 4183 | | -2.497 | | 0.265 | | 0 | | 0.09 |  |
| 4186 | | -6.927 | | 0.021 | | 0 | | 0.005 |  |
| 4187 | | -3.825 | | 0.119 | | 0 | | 0.022 |  |
| 4191 | | -6.929 | | 0.021 | | 0 | | 0.004 |  |
| 4192 | | -3.825 | | 0.112 | | 0 | | 0.041 |  |
| 4193 | | -4.474 | | 0.155 | | 0 | | 0.057 |  |
| 4194 | | -4.614 | | 0.076 | | 0 | | 0.024 |  |
| 4195 | | -3.137 | | 0.227 | | 0 | | 0.071 |  |
| 4204 | | -4.656 | | 0.085 | | 0 | | 0.023 |  |
| 4207 | | -4.613 | | 0.086 | | 0 | | 0.036 |  |
| 4212 | | -6.92 | | 0.021 | | 0 | | 0.005 |  |
| 4213 | | -5.738 | | 0.037 | | 0 | | 0.009 |  |
| 4222 | | -4.614 | | 0.076 | | 0 | | 0.027 |  |
| 4223 | | -3.825 | | 0.125 | | 0 | | 0.037 |  |
| 4226 | | -4.793 | | 0.102 | | 0 | | 0.011 |  |
| 4227 | | -6.925 | | 0.024 | | 0 | | 0.005 |  |
| 4228 | | -2.496 | | 0.264 | | 0 | | 0.09 |  |
| 4230 | | -2.328 | | 0.274 | | 0 | | 0.087 |  |
| 4232 | | -2.329 | | 0.274 | | 0 | | 0.085 |  |
| 4236 | | -4.982 | | 0.071 | | 0 | | 0.015 |  |
| 4237 | | -2.102 | | 0.314 | | 0 | | 0.095 |  |
| 4245 | | -4.63 | | 0.076 | | 0 | | 0.024 |  |
| 4246 | | -4.253 | | 0.134 | | 0 | | 0.036 |  |
| 4249 | | -4.283 | | 0.131 | | 0 | | 0.032 |  |
| 4253 | | -4.675 | | 0.085 | | 0 | | 0.023 |  |
| 4258 | | -2.338 | | 0.274 | | 0 | | 0.085 |  |
| 4263 | | -3.821 | | 0.114 | | 0 | | 0.022 |  |
| 4264 | | -5.758 | | 0.037 | | 0 | | 0.009 |  |
| 4267 | | -5.758 | | 0.037 | | 0 | | 0.006 |  |
| 4273 | | -3.839 | | 0.111 | | 0 | | 0.042 |  |
| 4274 | | -2.393 | | 0.32 | | 0 | | 0.094 |  |
| 4279 | | -4.948 | | 0.071 | | 0 | | 0.014 |  |
| 4282 | | -9.597 | | 0.004 | | 0 | | 0 |  |
| 4284 | | -4.472 | | 0.088 | | 0 | | 0.03 |  |
| 4286 | | -2.338 | | 0.274 | | 0 | | 0.085 |  |
| 4287 | | -3.839 | | 0.111 | | 0 | | 0.027 |  |
| 4288 | | -3.839 | | 0.111 | | 0 | | 0.022 |  |
| 4299 | | -2.338 | | 0.274 | | 0 | | 0.085 |  |
| 4300 | | -4.464 | | 0.157 | | 0 | | 0.059 |  |
| 4305 | | -4.231 | | 0.134 | | 0 | | 0.05 |  |
| 4308 | | -3.302 | | 0.196 | | 0 | | 0.058 |  |
| 4309 | | -4.18 | | 0.099 | | 0 | | 0.018 |  |
| 4310 | | -2.102 | | 0.313 | | 0 | | 0.094 |  |
| 4311 | | -5.758 | | 0.037 | | 0 | | 0.008 |  |
| 4317 | | -2.091 | | 0.315 | | 0 | | 0.095 |  |
| 4321 | | -6.952 | | 0.021 | | 0 | | 0.004 |  |
| 4323 | | -9.597 | | 0.004 | | 0 | | 0.001 |  |
| 4328 | | -5.758 | | 0.037 | | 0 | | 0.005 |  |
| 4331 | | -6.949 | | 0.024 | | 0 | | 0.005 |  |
| 4332 | | -3.616 | | 0.179 | | 0 | | 0.042 |  |
| 4335 | | -5.695 | | 0.176 | | 0.256 | | 0.082 |  |
| 4343 | | -3.839 | | 0.111 | | 0 | | 0.028 |  |
| 4355 | | -5.758 | | 0.037 | | 0 | | 0.011 |  |
| 4356 | | -2.496 | | 0.264 | | 0 | | 0.092 |  |
| 4357 | | -2.338 | | 0.274 | | 0 | | 0.081 |  |
| 4359 | | -6.324 | | 0.051 | | 0 | | 0.015 |  |
| 4362 | | -7.677 | | 0.012 | | 0 | | 0.004 |  |
| 4364 | | -9.341 | | 0.006 | | 0 | | 0 |  |
| 4371 | | -9.274 | | 0.006 | | 0 | | 0.001 |  |
| 4374 | | -3.839 | | 0.111 | | 0 | | 0.023 |  |
| 4379 | | -3.839 | | 0.118 | | 0 | | 0.025 |  |
| 4381 | | -3.347 | | 0.172 | | 0 | | 0.06 |  |
| 4385 | | -3.839 | | 0.111 | | 0 | | 0.028 |  |
| 4386 | | -4.63 | | 0.076 | | 0 | | 0.024 |  |
| 4389 | | -2.104 | | 0.313 | | 0 | | 0.093 |  |
| 4390 | | -4.197 | | 0.099 | | 0 | | 0.018 |  |
| 4394 | | -4.633 | | 0.076 | | 0 | | 0.023 |  |
| 4395 | | -5.758 | | 0.039 | | 0 | | 0.028 |  |
| 4396 | | -5.758 | | 0.037 | | 0 | | 0.027 |  |
| 4401 | | -3.839 | | 0.111 | | 0 | | 0.022 |  |
| 4403 | | -2.504 | | 0.265 | | 0 | | 0.08 |  |
| 4404 | | -14.71 | | 0.008 | | 0 | | 0.002 |  |
| 4406 | | -3.839 | | 0.111 | | 0 | | 0.029 |  |
| 4411 | | -2.483 | | 0.267 | | 0 | | 0.092 |  |
| 4412 | | -2.333 | | 0.274 | | 0 | | 0.1 |  |
| 4415 | | -2.496 | | 0.265 | | 0 | | 0.091 |  |
| 4417 | | -3.791 | | 0.185 | | 0.13 | | 0.09 |  |
| 4423 | | -4.468 | | 0.088 | | 0 | | 0.03 |  |
| 4429 | | -3.177 | | 0.223 | | 0 | | 0.088 |  |
| 4436 | | -5.66 | | 0.067 | | 0 | | 0.014 |  |
| 4438 | | -4.558 | | 0.113 | | 0 | | 0.026 |  |
| 4445 | | -2.339 | | 0.274 | | 0 | | 0.073 |  |
| 4448 | | -17.274 | | 0 | | 0 | | 0 |  |
| 4452 | | -6.306 | | 0.031 | | 0 | | 0.003 |  |
| 4453 | | -3.839 | | 0.111 | | 0 | | 0.022 |  |
| 4462 | | -3.839 | | 0.111 | | 0 | | 0.024 |  |
| 4466 | | -2.331 | | 0.274 | | 0 | | 0.058 |  |
| 4472 | | -3.611 | | 0.18 | | 0 | | 0.04 |  |
| 4476 | | -4.984 | | 0.07 | | 0 | | 0.015 |  |
| 4480 | | -3.839 | | 0.111 | | 0 | | 0.044 |  |
| 4481 | | -3.685 | | 0.121 | | 0 | | 0.035 |  |
| 4483 | | -2.336 | | 0.274 | | 0 | | 0.09 |  |
| 4484 | | -5.758 | | 0.037 | | 0 | | 0.008 |  |
| 4486 | | -3.839 | | 0.111 | | 0 | | 0.025 |  |
| 4487 | | -6.76 | | 0.025 | | 0 | | 0.007 |  |
| 4499 | | -4.632 | | 0.086 | | 0 | | 0.035 |  |
| 4502 | | -4.642 | | 0.076 | | 0 | | 0.015 |  |
| 4504 | | -6.177 | | 0.104 | | 0 | | 0.036 |  |
| 4506 | | -3.839 | | 0.111 | | 0 | | 0.023 |  |
| 4510 | | -3.839 | | 0.118 | | 0 | | 0.025 |  |
| 4515 | | -4.674 | | 0.075 | | 0 | | 0.014 |  |
| 4516 | | -4.272 | | 0.09 | | 0 | | 0.031 |  |
| 4520 | | -3.839 | | 0.111 | | 0 | | 0.023 |  |
| 4521 | | -3.76 | | 0.186 | | 0.125 | | 0.085 |  |
| 4522 | | -7.476 | | 0.019 | | 0 | | 0.003 |  |
| 4526 | | -2.496 | | 0.265 | | 0 | | 0.091 |  |
| 4527 | | -4.936 | | 0.071 | | 0 | | 0.018 |  |
| 4529 | | -4.991 | | 0.07 | | 0 | | 0.013 |  |
| 4530 | | -4.59 | | 0.083 | | 0 | | 0.026 |  |
| 4533 | | -5.758 | | 0.037 | | 0 | | 0.008 |  |
| 4534 | | -7.977 | | 0.08 | | 0 | | 0.028 |  |
| 4535 | | -3.612 | | 0.179 | | 0 | | 0.042 |  |
| 4536 | | -5.758 | | 0.043 | | 0 | | 0.016 |  |
| 4541 | | -3.57 | | 0.183 | | 0 | | 0.044 |  |
| 4550 | | -10.17 | | 0.003 | | 0 | | 0.001 |  |
| 4551 | | -4.425 | | 0.089 | | 0 | | 0.032 |  |
| 4554 | | -3.839 | | 0.111 | | 0 | | 0.033 |  |
| 4556 | | -2.338 | | 0.274 | | 0 | | 0.07 |  |
| 4563 | | -2.331 | | 0.274 | | 0 | | 0.093 |  |
| 4565 | | -5.609 | | 0.067 | | 0 | | 0.013 |  |
| 4575 | | -10.648 | | 0.008 | | 0.078 | | 0.006 |  |
| 4578 | | -6.926 | | 0.025 | | 0 | | 0.01 |  |
| 4581 | | -6.928 | | 0.025 | | 0 | | 0.009 |  |
| 4589 | | -3.825 | | 0.111 | | 0 | | 0.033 |  |
| 4591 | | -5.731 | | 0.037 | | 0 | | 0.013 |  |
| 4593 | | -4.656 | | 0.075 | | 0 | | 0.018 |  |
| 4594 | | -5.738 | | 0.037 | | 0 | | 0.005 |  |
| 4595 | | -3.825 | | 0.111 | | 0 | | 0.021 |  |
| 4597 | | -4.19 | | 0.098 | | 0 | | 0.016 |  |
| 4600 | | -2.097 | | 0.313 | | 0 | | 0.094 |  |
| 4604 | | -13.389 | | 0 | | 0 | | 0 |  |
| 4605 | | -8.897 | | 0.008 | | 0 | | 0.002 |  |
| 4613 | | -3.342 | | 0.271 | | 0 | | 0.067 |  |
| 4615 | | -3.825 | | 0.111 | | 0 | | 0.021 |  |
| 4616 | | -4.179 | | 0.093 | | 0 | | 0.022 |  |
| 4620 | | -5.738 | | 0.037 | | 0 | | 0.01 |  |
| 4621 | | -5.738 | | 0.037 | | 0 | | 0.009 |  |
| 4630 | | -4.415 | | 0.089 | | 0 | | 0.032 |  |
| 4639 | | -6.986 | | 0.021 | | 0 | | 0.002 |  |
| 4643 | | -2.33 | | 0.274 | | 0 | | 0.095 |  |
| 4647 | | -3.825 | | 0.111 | | 0 | | 0.026 |  |
| 4648 | | -2.328 | | 0.274 | | 0 | | 0.098 |  |
| 4649 | | -2.329 | | 0.274 | | 0 | | 0.089 |  |
| 4665 | | -2.329 | | 0.283 | | 0 | | 0.085 |  |
| 4669 | | -2.21 | | 0.289 | | 0 | | 0.098 |  |
| 4670 | | -8.364 | | 0.023 | | 0.087 | | 0.013 |  |
| 4672 | | -3.825 | | 0.111 | | 0 | | 0.032 |  |
| 4676 | | -4.904 | | 0.072 | | 0 | | 0.018 |  |
| 4688 | | -2.493 | | 0.265 | | 0 | | 0.087 |  |
| 4689 | | -3.825 | | 0.112 | | 0 | | 0.033 |  |
| 4690 | | -6.613 | | 0.038 | | 0 | | 0.011 |  |
| 4692 | | -3.825 | | 0.119 | | 0 | | 0.023 |  |
| 4698 | | -6.026 | | 0.077 | | 0.125 | | 0.052 |  |
| 4699 | | -4.626 | | 0.119 | | 0.152 | | 0.078 |  |
| 4703 | | -6.767 | | 0.02 | | 0 | | 0.006 |  |
| 4709 | | -8.481 | | 0.058 | | 0.1 | | 0.021 |  |
| 4712 | | -6.114 | | 0.066 | | 0.088 | | 0.023 |  |
| 4719 | | -3.825 | | 0.111 | | 0 | | 0.022 |  |
| 4720 | | -5.738 | | 0.037 | | 0 | | 0.012 |  |
| 4722 | | -3.945 | | 0.105 | | 0 | | 0.037 |  |
| 4725 | | -4.407 | | 0.16 | | 0 | | 0.059 |  |
| 4726 | | -2.495 | | 0.265 | | 0 | | 0.08 |  |
| 4729 | | -3.825 | | 0.111 | | 0 | | 0.044 |  |
| 4730 | | -3.825 | | 0.111 | | 0 | | 0.028 |  |
| 4733 | | -4.228 | | 0.091 | | 0 | | 0.021 |  |
| 4739 | | -5.738 | | 0.037 | | 0 | | 0.007 |  |
| 4741 | | -6.268 | | 0.028 | | 0 | | 0.005 |  |
| 4743 | | -3.804 | | 0.112 | | 0 | | 0.032 |  |
| 4754 | | -11.345 | | 0.031 | | 0.05 | | 0.016 |  |
| 4755 | | -5.738 | | 0.037 | | 0 | | 0.008 |  |
| 4758 | | -3.141 | | 0.226 | | 0 | | 0.089 |  |
| 4759 | | -2.486 | | 0.264 | | 0 | | 0.092 |  |
| 4764 | | -4.181 | | 0.093 | | 0 | | 0.022 |  |
| 4765 | | -9.254 | | 0.006 | | 0 | | 0.001 |  |
| 4767 | | -3.549 | | 0.184 | | 0 | | 0.042 |  |
| 4770 | | -4.66 | | 0.075 | | 0 | | 0.013 |  |
| 4772 | | -2.33 | | 0.274 | | 0 | | 0.086 |  |
| 4775 | | -4.617 | | 0.076 | | 0 | | 0.023 |  |
| 4777 | | -3.825 | | 0.112 | | 0 | | 0.041 |  |
| 4781 | | -5.074 | | 0.054 | | 0 | | 0.018 |  |
| 4786 | | -3.825 | | 0.111 | | 0 | | 0.018 |  |
| 4789 | | -5.738 | | 0.037 | | 0 | | 0.008 |  |
| 4795 | | -2.329 | | 0.274 | | 0 | | 0.095 |  |
| 4799 | | -5.738 | | 0.037 | | 0 | | 0.006 |  |
| 4803 | | -2.462 | | 0.267 | | 0 | | 0.096 |  |
| 4808 | | -4.243 | | 0.134 | | 0 | | 0.044 |  |
| 4809 | | -4.733 | | 0.104 | | 0 | | 0.019 |  |
| 4810 | | -3.825 | | 0.111 | | 0 | | 0.03 |  |
| 4817 | | -6.05 | | 0.067 | | 0.12 | | 0.047 |  |
| **Codon** | | **SLAC dN-dS** | | **SLAC *p*-Value** | | **FEL dN-dS** | | **FEL *p*-Value** |  |
| 1 | | -38.886 | | 0 | | -1.798 | | | 1 |

*Codon = position on the coding region of canine distemper virus genome without stop codons
